# The Impact of Plant-Based Coatings in “ROCHA” Pear Preservation during Cold Storage: A Metabolomic Approach

**DOI:** 10.3390/foods9091299

**Published:** 2020-09-15

**Authors:** Alexandre M. A. Fonseca, Cindy Dias, Ana L. Amaro, Nélson Isidoro, Manuela Pintado, Armando J. D. Silvestre, Sílvia M. Rocha

**Affiliations:** 1 LAQV-REQUIMTE, Department of Chemistry, University of Aveiro, 3810-193 Aveiro, Portugal; alexandrefonseca@ua.pt; 2 CICECO, Department of Chemistry, University of Aveiro, 3810-193 Aveiro, Portugal; armsil@ua.pt; 3 Escola Superior de Biotecnologia, CBQF-Centro de Biotecnologia e Química Fina–Laboratório Associado, Universidade Católica Portuguesa, Rua de Diogo Botelho, 1327, 4169-005 Porto, Portugal; cdias@porto.ucp.pt (C.D.); aamaro@porto.ucp.pt (A.L.A.); mpintado@porto.ucp.pt (M.P.); 4 Cooperativa Agrícola dos Fruticultores do Cadaval, CRL (COOPVAL), EN 115, Km 26 2550-108 Cadaval, Portugal; nelson.isidoro@coopval.com

**Keywords:** “Rocha” pear, cold storage, plant-based coatings, GC×GC-ToFMS, conjugated trienols

## Abstract

Although new storage technologies have been emerging in recent years, preservation of pear (*Pyrus communis* L.) remains a challenge for suppliers. Maintenance of desired organoleptic properties throughout cold storage using non-chemical strategies has been investigated and the use of edible coatings has shown potential to delay fruit quality deterioration during cold storage. Thus, the objective of this study is to evaluate the impact of pectin coatings including plant extracts, in “Rocha” pear (*Pyrus communis* L. cv. Rocha) preservation. A four-month pilot scale assay was performed in both dynamic controlled atmosphere (DCA) (−0.5 °C, 0.5% O_2_, and 0.4% CO_2_) and normal atmospheric (NA) conditions (2 °C). For each storage condition, the following three coatings were tested: pectin (3% *w*/*v*) (PCT), pectin (3% *w*/*v*) + strawberry tree leaves extract (9.5 mg/mL) (CT1), and pectin (3% *w*/*v*) + apple pomace extract (16 mg/mL) (CT2). Volatile compounds, potentially related to aroma or ripening status of “Rocha” pear, were monitored alongside with conjugated trienols (CTs) and maturity parameters. The combination of DCA conditions and the application of pectin coatings were able to reduce the release of Rocha pear volatiles associated with ripening status, (particularly esters and sesquiterpenes), as well as reduce CTs, which could contribute to the preservation of Rocha pear for longer periods.

## 1. Introduction

“Rocha” pear (*Pyrus communis* L. cv. Rocha), a DOP (denomination of protected origin) cultivar from the western region of Portugal, is the fourth pear cultivar in Europe and the main cultivar in Portugal, with an average production of 173,000 tons/year, contributing to significant revenues for the Portuguese economy (120–130 million euros per year) [1,2]. Although “Rocha” pear is harvested in August, the aim is to have it available to the consumers for as long as possible. To achieve this, postharvest quality of pears must be maintained across storage through the reduction of fruit metabolic processes without compromising some desired changes such as the development of aroma and taste. In addition to traditional cold storage at around 0 °C, the use of 1-methylcyclopropene (1-MCP) and methodologies that modify storage atmospheric conditions by applying static (controlled atmosphere, CA) or dynamic (dynamic controlled atmosphere, DCA) partial pressures of oxygen (*p*O_2_) and carbon dioxide (*p*CO_2_) have been used to extend the storage life of pears [3]. However, an affordable, consistently reliable, and easily applied technology that preserves the organoleptic characteristics of pears for a prolonged period has yet to be achieved.

The main disadvantage of prolonged cold exposure is the development of postharvest disorders that are responsible for fruit losses [4]. In “Rocha” pears, scald symptoms usually appear after four months of cold storage without atmosphere control, and scald occurrence is highly dependent on preharvest factors [5]. Compounds such as the sesquiterpene α-farnesene, conjugated trienes, and 6-methyl-5-hepten-2-one (MHO) have been extensively associated with superficial scald (SC) development (a cold-derived disorder) [6]. As ethylene production is reduced, α-farnesene metabolism is reduced, accumulating at high levels in the external wax layer, due to its lipophilic characteristics [7,8]. Further autoxidation of α-farnesene to conjugated trienes and trienols is thought to be the main cause of SC [4,8,9]. Several authors have demonstrated the relationship between α-farnesene and conjugated trienes in pears along with storage in which the accumulation of these oxidation products is associated with the decrease of α-farnesene content [5,7]. Production of MHO, resulting from autoxidation of conjugated trienes and trienols, is thought to be an important aspect of SC development, although its role is not yet clear [8].

In recent years, the use of edible coatings, in particular, polysaccharide-based coatings (starch, chitosan, xanthan gum, pectin, cellulose derivatives, and alginate), has shown great potential to delay fruit quality deterioration during cold storage due to their potential to reduce respiration rates and create a gas barrier on the fruit surface [10,11,12,13]. In addition, coatings can be supplemented with food compatible (and ideally natural) antioxidants to delay and reduce oxidative-related disorders [14]. Such natural antioxidants have been researched by several sources [15] and can possibly scavenge oxidation-linked damages by binding free radicals, thus protecting fruits from oxidative process [16]. Sharma and Rao [17] reported that a xanthan gum-based edible coating incorporated with cinnamic acid led to a significant reduction of the oxidative browning process, which ultimately preserved the organoleptic characteristics of fresh-cut pears. Recently, Dias et al. [18] selected, amongst fifteen natural-based extracts with antioxidant properties, strawberry tree (leaves and branches), as well as apple pomace methanolic extracts, as the most promising mitigators of fruit browning in fresh-cut pears. Total phenolic content of strawberry tree extracts (leaves and branches) were 207.9 mg and 104.1 mg GAE/g extract, respectively, and 6.8 mg GAE/g extract for apple pomace. These plant-based extracts promoted the reduction in the IC_50_ against polyphenoloxidase and peroxidase activities. This study highlights the opportunity of by-products and agricultural waste extracts as novel anti-browning agents.

Pear aroma, which is one of the most important attributes to consumer acceptance, has been shown to be influenced by storage conditions and postharvest treatments [19,20,21,22]. The volatile organic compounds (VOCs) that contribute to the fruit aroma are generated through metabolic pathways during ripening, harvest, postharvest, and storage and vary according to the cultivar [23]. “Rocha” pear aroma is mainly composed of esters, aldehydes, and alcohols that originate from amino acid and fatty acid metabolism during ripening. During maturation, the enzyme alcohol acyl-CoA transferase activity increases, combining alcohols and CoA derivatives of short to medium chain length fatty acids to form aldehydes, then alcohols and finally esters [23,24]. A previous study that analyzed the volatile composition of ripe Rocha pears identified a total of 25 compounds, belonging mainly to the mentioned families and showed that the flavor-important aliphatic esters, butyl acetate and hexyl acetate, were the main compounds, accounting for more than 60% of the total volatile production [25]. Gomes et al. [26] further showed that the levels of butyl acetate in “Rocha” pears were not affected by storage temperature (0–15 °C), while hexyl acetate was significantly higher at 0 °C, but could be severely affected by hypoxic atmospheres, which led to an increase in the ethanol and ethyl acetate levels.

During fruit postharvest storage, significant compositional changes occur that are species and cultivar dependent. Some of those alterations reflect the reconfiguration of fruit metabolism as a consequence of the abiotic and biotic stresses encountered during storage conditions. Metabolomic platforms, which are able to provide a profile of small molecules in a biological system that reflects its biological status, offer the possibility to improve our knowledge about the molecular mechanisms underlying fruit senescence which are affected under commercial storage conditions and to optimize molecular mechanisms according to a specific species or cultivar [27].

Gas chromatography-mass spectrometry (GC-MS) has been used to study the composition of fresh fruits VOCs, and to study physiological aspects of fruit maturation [23]. However, with this technique, only a small part of VOCs has been identified [28]. Comprehensive two-dimensional gas chromatography (GC×GC) is one of the most powerful separation techniques for VOCs, for the simultaneous determination of both major and trace components. This technique includes an interface, i.e., modulator, that physically connects a primary and secondary column and operates by preserving the separation obtained in the first dimension (first column), while achieving additional separation in the second dimension [29]. Such configuration provides significant signal enhancement, achieves highly effective fingerprinting, and has the potential to provide information encrypted in complex patterns of volatiles regarding sample origin, technological signature, and aroma [5,28,30]. Hence, with a single analysis, this analytical method provides different molecular information, allowing a comprehensive investigation of the impact of several factors (cultivar, environment, cold storage, postharvest strategies applied to prolong fruit quality, among others), as well as quality control in fruits.

Accordingly, this work was undertaken as a pilot study to evaluate the impact and potential of pectin coatings in the preservation of “Rocha” pears during cold storage. Since physiological disorders and fruit senescence of pears during cold storage are more prevalent after four months of storage [5], this work focused only on the storage period when pears were more susceptible to be affected. The coatings tested consisted of pectin alone or pectin enriched with either strawberry tree leaves or apple pomace extracts and were applied after six months of storage. A pilot scale assay was performed in the subsequent four months of storage in both DCA (−0.5 °C, 0.5% O_2_, and 0.4% CO_2_) and normal atmosphere (NA) conditions (2 °C) and a metabolomic based approach was performed using an advance gas chromatographic technique (GC×GC-ToFMS, comprehensive two-dimensional gas chromatography combined with time of flight mass spectrometry). Effects on the accumulation of conjugated trienols (CTs) and maturity parameters were also evaluated.

## 2. Materials and Methods

### 2.1. Samples, Materials, and Reagents

Pear fruit (*Pyrus communis* L. cv. Rocha) were harvested at an optimal maturation stage (firmness = 4.56 ± 0.56 N and total soluble solids (TSS) = 12.97 ± 0.27) from a commercial orchard in Cadaval (N 39° 25’, W 8° 54’, 120 m), Portugal. Pears were transported immediately after harvest to a commercial packinghouse and hand-sorted to select undamaged fruit of uniform size.

Then, the fruit were stored in DCA conditions (−0.5 °C, 0.5% O_2_, and 0.4% CO_2_) until use. Fresh strawberry tree (*Arbutus unedo* L.) leaves were separated from branches and stored at −20 °C. Before extraction, leaves were freeze-dried and ground into powder (IKA A10 analytical grinder). Apple pomace was stored at −80 °C upon arrival and used directly for extraction after thawing.

HPLC-grade ethanol and *n*-hexane (99%) were supplied by Carlo Erba Reagents S.A.S (Val-de-Reuil Cedex, France). Food grade pectin from citrus peel was purchased from Sigma-Aldrich. Quartz glass microplate with 96 wells (300 µL) was purchased from Hëllma Analytics. The retention index probe (a series of C8 to C20 straight-chain alkanes, in *n*-hexane) was supplied by Fluka (Buchs, Switzerland). The solid-phase microextraction (SPME) holder for manual sampling and the fiber coating used were purchased from Supelco (Bellefonte, PA). The SPME device included a 1 cm StableFlex™ fused silica fiber, coated with partially cross-linked 50/30 μm divinylbenzene/Carboxen™/poly(dimethylsiloxane) (DVB/CAR/PDMS). The fiber presents a wide range capacity for adsorbing and absorbing compounds with different physicochemical properties, with molecular weights ranging from 40 to 275. According to the manufacturer’s recommendations, the SPME fiber was initially conditioned once at 270 °C for 60 min in the GC injector, and daily for 10 min at 250 °C.

### 2.2. Plant-Based Coating

#### 2.2.1. Extracts Preparation

Extraction procedure was performed according to Dias et al. [18] with modifications. Each plant material (strawberry tree leaves or apple pomace) was consecutively extracted (*m*/*v*, 1:20) three times (with renewal of solvent between extractions) with an ethanol/water mixture, 70/30 (*v*/*v*) for 1 h, at 25 °C, under constant stirring. Then, the suspensions were vacuum filtered, and the EtOH removed in a rotary evaporator (Büchi rotavapor R-114). Finally, extracts were freeze-dried. Extraction yields obtained for strawberry tree leaves and apple pomace were 13.7 and 4.7 g extract/100 g of fresh weight, respectively.

#### 2.2.2. Coating Formulation

After extraction, the optimal concentration, considering solubility and color, was determined for each extract. For further analysis, concentration of 9.53 and 16 mg/mL were used for strawberry tree leaves and apple pomace, respectively. Pectin coating was prepared according to a method previously described, with modifications [31]. Coatings were prepared by dissolving pectin (3% *w*/*v*) in distilled water and heated at 60 °C while stirring until the solution became clear. After cooling down to room temperature, plant extracts were dissolved at the concentration previously optimized. The coatings prepared were the following: PCT (pectin at 3% *w*/*v*), CT1 (pectin at 3% *w/v* + strawberry tree (*Arbutus unedo* L.) leaves extract at 9.53 mg/mL), and CT2 (pectin at 3% *w/v* + apple pomace extract at 16 mg/mL).

### 2.3. Pilot-Scale Storage Assay

**Figure 1:**
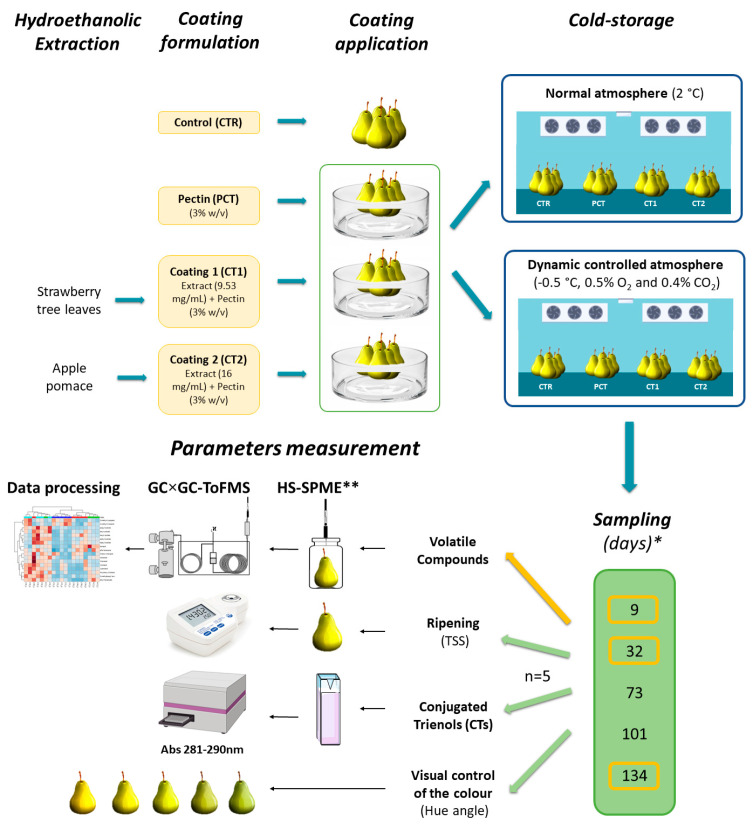
Schematic representation of the experimental design of the 4-month pilot-scale assay. Volatile compound analysis was performed for 9, 32, and 134 days, while conjugated trienols (CTs) determination, hue angle and total soluble solids (TSS) were performed for all available sampling times. * Days after coating application; ** after overnight pre-equilibrium.

The impact of the application of the developed plant-based coatings was evaluated in a pilot scale storage assay with a duration of 134 days, according to the experimental procedure shown in Figure 1. A total of 750 pears, which had been submitted to a prior six-month storage period, was randomly selected and used as sample for the pilot scale storage assay. Adding to a control condition of uncoated pears (CTR), samples were coated with PCT, CT1, and CT2 by submersion in coating solutions for 1 min. Afterwards, fruits were suspended to drain the excess solution on the their surface and left to dry at room temperature (23 °C), for 2 h. Pears of the 4 conditions were stored in both DCA (−0.5 °C, 0.5% O_2_, and 0.4% CO_2_), and NA (2 °C) conditions during a 4-month period. Randomly selected pears of the 4 conditions of each kind of storage were sampled at 5 different times (9, 32, 73, 101, and 134 days). The analyzed parameters were comprised of pear headspace volatile compounds (at 9, 32, and 134 days), ripening indicators (total soluble solids (TSS) and hue angle), and CTs quantification for all sampling times.

### 2.4. Conjugated Trienols Analysis

CTs’ extraction and quantification were adapted from a previous publication [5]. For each pear, the peel was removed and immediately stored at −80 °C and only thawed before analysis. Peel disks (20) with a 5 mm diameter were excised from each sample and placed in 2 mL centrifuge tubes. Hexane (1.3 mL) was added to the peel disks and incubated at room temperature for 10 min. After incubation, the solvent was filtered through a sterile 0.20 μm cellulose acetate filter and transferred in triplicate into the quartz microplate. Absorbance at 291 and 280 nm was recorded using a BioTek Eon^TM^ Microplate reader. CTs’ concentrations were calculated using the molar extinction coefficients ε_281–290nm_ = 25,000 and expressed as nmol/cm^2^ [32]. Two-way analysis of variance (ANOVA) was performed to assess differences of CTs’ contents using condition and time of storage as factors. Fisher’s least significant difference (LSD) was conducted for mean comparisons. Differences with a probability value of <0.05 were considered to be significant and all data were reported as mean ± SD. ANOVA analyses were done using STATISTICA software (StatSoft, v.8.0, Tulsa, OK, USA).

### 2.5. Assessment of Fruit Maturity

TSS, expressed as °Brix, were measured in the fruit juice by a digital refractometer PR1 ATAGO Co., Ltd. (Tokyo, Japan). Two-way ANOVA was performed to assess differences in the content of TSS using condition and time of storage as factors. Fisher’s LSD was conducted for mean comparisons. Differences with a probability value of <0.05 were considered to be significant and all data were reported as mean ± SD (standard deviation). ANOVA analyses were done using STATISTICA software (StatSoft, v.8.0, Tulsa, OK, USA).

### 2.6. Surface Color Determination

Fruit surface color was measured with a CR-400 colorimeter (Konica Minolta, Osaka, Japan) using the D65 illuminant and the CIE (Commission Internationale de l’Eclairage) parameters (L*, a*, b*). Hue was calculated as hue angle (h° = arctan(b*/a*)). Two measurements were performed on opposite sides of the widest part of each fruit. Two-way ANOVA was performed to assess differences of the pilot-scale assay using treatment and days of storage as factors. Fisher’s LSD was conducted for mean comparisons. Differences with a probability value of <0.05 were considered significant and all data were reported as mean ± SD. ANOVA analyses were done using STATISTICA software (StatSoft, v.8.0, Tulsa, OK, USA).

### 2.7. Volatile Profile Determination

#### 2.7.1. HS-SPME/GC×GC-ToFMS

GC×GC-ToFMS was employed to study in-depth the “Rocha” pear volatile profile variation across cold storage. Sampling, reporting of chemical analysis to data preprocessing, pretreatment, processing, and interpretation were performed according to the metabolomics standards initiative (MSI), as detailed below [33].

HS-SPME/GC×GC-ToFMS methodology was adapted from other studies [34,35] with optimization of the following parameters: time of pre-equilibrium and HS-SPME extraction, stationary phase of the columns, temperature ramp; modulation time, transfer line temperature, and drag gas flow. The effects of these parameters were evaluated based on visual analysis of the chromatograms through the chromatographic resolution, relative abundance of the principal peaks, and chromatogram overall structure.

Each analyzed pear was randomly selected and sealed in a 1 L airtight jar fitted with a rubber septum in the lid. Jars were closed and kept overnight at room temperature to reach equilibrium. Then, each jar was immersed in a water bath adjusted to 25.0 ± 0.1 °C and the DVB/CAR/PDMS SPME fiber was inserted in the headspace trough the septum, for 1 h. The volatiles adsorbed and absorbed on the SPME fiber coating were determined using a LECO Pegasus 4D GC×GC-ToFMS system (LECO, St. Joseph, MI, USA) consisting of an Agilent GC 7890A gas chromatograph (Agilent Technologies, Inc., Wilmington, DE, USA), with a dual stage jet cryogenic modulator (licensed from Zoex) and a secondary oven, and a mass spectrometer equipped with a ToF analyzer. After the extraction/concentration step, the SPME fiber was manually introduced into the port at 250 °C, for 3 min, for analytes desorption. The injection port was lined with a 0.75 mm I.D. glass liner. Splitless conditions (30 s) were used. An Equity-5 column (30 m × 0.32 mm I.D., 0.25 μm film thickness, Supelco, Bellefonte, PA, USA) was used as the first-dimension column (^1^D) and a DB-FFAP column (0.79 m × 0.25 mm I.D., 0.25 μm film thickness, J&W Scientific Inc., Folsom, CA, USA) was used as the second-dimension column (^2^D). The carrier gas was helium at a constant flow rate of 2.50 mL/min. The primary oven temperature was programmed from 40 °C (1 min) to 160 °C, at 3 °C/min, followed by a second ramp from 160 °C to 225 °C (2 min), at 15 °C/min. Secondary oven program was 5 °C offset above the primary one. The MS transfer line and MS source temperatures were both set at 250 °C. The modulation period was 5 s, keeping the modulator at 20 °C offset above primary oven, with hot and cold pulses of 0.90 and 1.60 s, respectively. The mass spectrometer ran in EI mode at 70 eV, using an *m*/*z* range of 35‒300.

Total ion chromatograms were processed using the automated data processing software ChromaTOF^®^ (LECO, St Joseph, MI, USA) at a signal-to-noise threshold of 100. Spectral deconvolution was computationally processed, being intended to reconstruct clean mass spectra for each component; whereas the GC×GC peak area was obtained by transforming the series of side-by-side second-dimension chromatograms into a two-dimensional chromatogram, the GC peak area being proportional to the generated signal intensity [36]. Contour plots were used to evaluate the general separation quality and for manual peak identification. For identification purposes, the mass spectrum of each detected metabolite was compared with mass spectral libraries, namely an in-house library of standards and two commercial databases (Wiley 275 and U.S. National Institute of Science and Technology (NIST) V. 2.0, Mainlib and Replib). A mass spectral match factor, similarity >700/1000, was used to decide whether a peak was correctly identified. Moreover, a manual analysis of mass spectra was performed, combining additional information such as the retention index (RI) value which was experimentally determined according to the van den Dool and Kratz equation [37]. A C8‒C20 *n*-alkanes series was used for RI determination, and these values were compared with those reported in the literature for chromatographic columns similar to the above mentioned ^1^D column. The calculated retention index (RI_calc_) only differed 0–5% as compared with the literature data (RI_lit_). Five independent samples of each condition were analyzed.

#### 2.7.2. Data Processing

A full data matrix consisting of 57 variables (metabolites) and 96 observations was constructed (Table S1, in Supplementary Materials). The 96 observations correspond to the 4 coating conditions of each type of storage at 3 times (9, 32, and 134 days), each one with 5 independent replicates. Using the MetaboAnalyst 3.0 (web interface) software (McGill University, Montreal, QC, Canada), autoscaling normalization of the data was applied and heatmap visualization was obtained on this matrix, using absolute GC peak area. Additionally, hierarchical clusters analysis (HCA) was also performed using the same software, to further examine the differences and similarities between each condition metabolite profiles. Ward’s minimum variance algorithm method and squared Euclidean distances were employed.

## 3. Results

### 3.1. Pear Chromatogram Contour Plot Analysis

**Figure 2:**
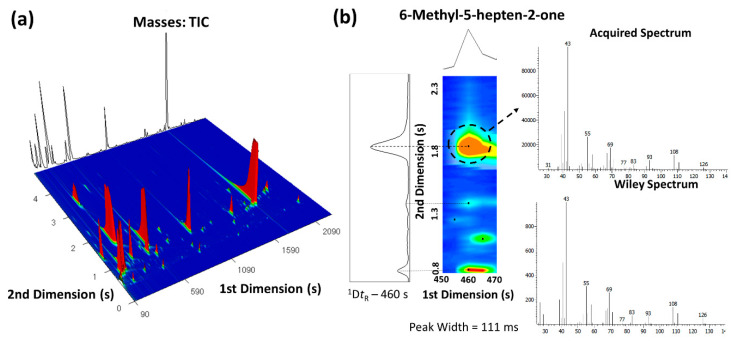
(**a**) Three-dimensional GC×GC total ion chromatogram plot of an uncoated pear at 9 days, used to illustrate the “Rocha” pear volatile profile; (**b**) Enlargement of a part of GC×GC chromatogram contour plot of the pear showing the separation of metabolites with the same retention time. The 111 milliseconds wide 6-methyl-5-hepten-2-one (MHO) GC×GC peak is easily defined and identified at a mass spectral acquisition of 125 spectra/s. Spectral quality at this high acquisition rate is maintained due to the ToFMS with continuous full-range mass spectral acquisition rate. As observed, the MHO mass spectrum is very similar (similarity value of 912/1000) as compared with the Wiley database.

A representative three-dimensional (3D) GC×G-ToFMS total ion chromatogram contour plot of “Rocha” pear is illustrated in Figure 2a. Here, it shows the pear headspace metabolite separation according to physicochemical characteristics, namely, through volatility (first dimension) and polarity (second dimension), when a non-polar and polar set of columns is used. In this way, a structured chromatogram is obtained, in which structurally related analytes occupy similar two-dimensional (2D) spaces. The GC×GC configuration also results in the resolution of many peaks that otherwise would be overlapped in a one-dimensional (1D) chromatogram.

An example of such advantage can be observed in Figure 2b. Analytes with similar volatility (^1^*t*_R_ – 460 s) such as MHO and β-myrcene can be effectively separated by the second column according to their polarity (^2^*t*_R_ of 1.894 and 0.850 s, respectively).

From each pear analyzed, a chromatogram, with approximate 550 instrumental features, was obtained. These features were constituted mostly by minor peaks and a few major ones. Major peaks detected on uncoated pears at nine days were 1-butanol, 1-hexanol, butyl acetate, pentyl acetate, hexyl acetate, butyl butanoate. and α- and β-farnesene (Table S1). From the total instrumental features obtained, a total of 57 compounds were identified and selected for further analysis. Selected metabolites are listed in Table 1 along with the respective odor descriptors obtained from literature. Selected compounds were comprised of metabolites belonging to several chemical families commonly identified in pear volatile profiles, namely, alcohols, aldehydes, esters, and mono- and sesquiterpenes [6]. Such chemical families are known to be the major volatiles emitted by pears and the main contributors to the pear aroma [6]. Among these, straight-chain esters, alcohols, and aldehydes, which are generally regarded to be a result of fatty acid metabolism, are produced in pears during fruit development and maturation [38]. From the set of compounds selected, esters were qualitatively the most representative chemical family. Other compounds associated with SC development such as α-farnesene and MHO were also identified [6].

For the selected compounds, at the first sampling time of the pilot scale assay (*t* = 9 days), uncoated pears’ headspace volatiles were mainly composed of sesquiterpenes (71.5%), followed by esters (23.0%), alcohols (3.7%), monoterpenes (0.8%), aldehydes (0.6%), and ketones (0.3%). A previous study on the headspace volatiles of ripe “Rocha” pears identified a total of 25 compounds with butyl acetate and hexyl acetate accounting for 66.5% of total volatiles peak area [25]. A total of 17 out of these 25 reported volatile compounds were successfully identified in the present study and included in the metabolites to be analyzed (Table 1).

### 3.2. Impact of Plant-Based Coatings on Previously Reported Volatile Compounds of “Rocha” Pear

**Figure 3:**
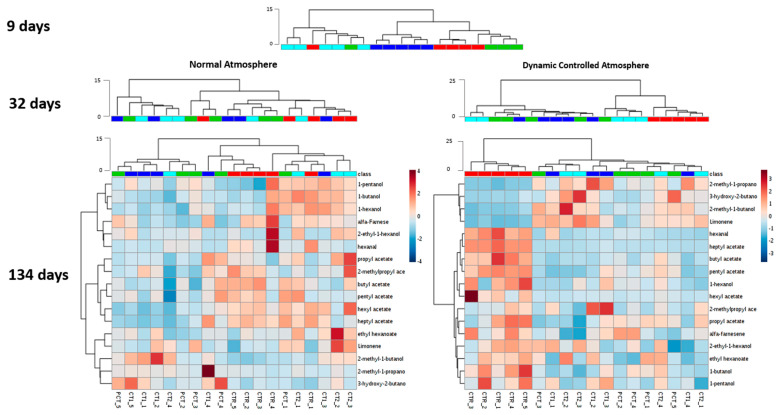
Dendrograms of the hierarchical clustering analysis (HCA) performed for previously reported volatile compounds of “Rocha” pear at 9, 32, and 134 days of the assay in both normal atmosphere (NA) and dynamic controlled atmosphere (DCA) storage conditions. Euclidean distances are included on the dendrogram *Y*-axis. Heatmap visualization at day 134 shows the relative content of each compound, illustrated through a chromatic scale (from low (dark blue) to high chromatographic area (dark red black)), which corresponds to its peak area normalized by autoscaling. 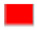 CTR (control); 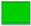 PCT (pectin); 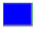 CT1 (coating 1); 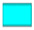 CT2 (coating 2).

In a first approach, the impact of the coatings on headspace volatile compounds previously identified in “Rocha” pear (Table 1) was assessed by cluster analysis. This analysis was performed at three sampling times (9, 32, and 134 days) for both storage conditions. The results obtained from the HCA analysis are presented in Figure 3 with a heatmap visualization of relative amounts of the analytes for the last sampling point (134 days). Heatmaps for all sampling points are provided in hte Supplementary Materials for NA (Figure S1) and DCA (Figure S2) conditions.

As seen in Figure 3, at nine days after coating application, when storage conditions have yet exerted little influence on pear physiology, an almost complete clustering of replicates according to coating condition is visible in the dendrogram. CT2 was shown to be the most distinctive condition because it was placed in a different branch with a higher Euclidean distance. The branch containing the remaining coating conditions was divided among CT1 samples and the two remaining conditions (CTR and PCT). CTR and PCT were found to be the most similar, which is probably because no plant extract was incorporated in them.

In subsequent times of analysis, a different clustering behavior was observed between the two storage conditions. In the case of NA, dendrograms obtained do not exhibit a sample clustering that can be associated with the coating conditions. In this case, the data suggest that the coating application and the kind of plant extract incorporated in the coating do not have a relevant impact on the release of the selected analytes after 32 days of storage. Differences observed in this case are only attributed to sample variability.

Regarding storage in DCA conditions, a different behavior is observed. In this case, coated samples become increasingly distinct over time relative to the control. This is particularly evident at the end of 134 days, when all control samples are clustered in one of the two main branches of the dendrogram. On the one hand, the other main branch contains samples of coated conditions (PCT, CT1, and CT2) without any clustering according to the type of coating applied. These samples exhibit a low Euclidean distance, which indicates a high similarity between them and suggests that plant extract incorporation on coatings does not have a relevant impact on the release of the analyzed compounds to the pear headspace. On the other hand, coating application by itself seems to have an impact on the release of such compounds since CTR samples are clustered at a higher distance from the remaining samples. Through the heatmap visualization, it is possible to verify that this difference is explained mainly by a reduction of the release of esters, such as butyl acetate and hexyl acetate, which are the most abundant compounds released by “Rocha” pears. In addition, 2-methyl-1-propanol, 2-methyl-1-butanol, 3-hydroxy-2-butanone, and limonene release are increased in coated pears.

### 3.3. Impact of Plant-Based Coatings on “Rocha” Pear Physiology during Storage

Adding to the evaluation of the variations on the release of the known “Rocha” pear volatile compounds caused by plant-based coatings, other parameters were monitored to assess possible impacts on fruit physiology of such coatings during fruit storage. Fruit maturity was evaluated by measuring the TSS content on pear juice and hue angle of pear surface, while the development of SC was assessed through CTs determination in fruit peel. To conclude, a broader metabolomic approach using the complete dataset of volatile compounds identified (Table 1), comprising both aroma and ripening related compounds, was used to evaluate metabolic alterations on fruits.

#### 3.3.1. Fruit Maturity

**Figure 4:**
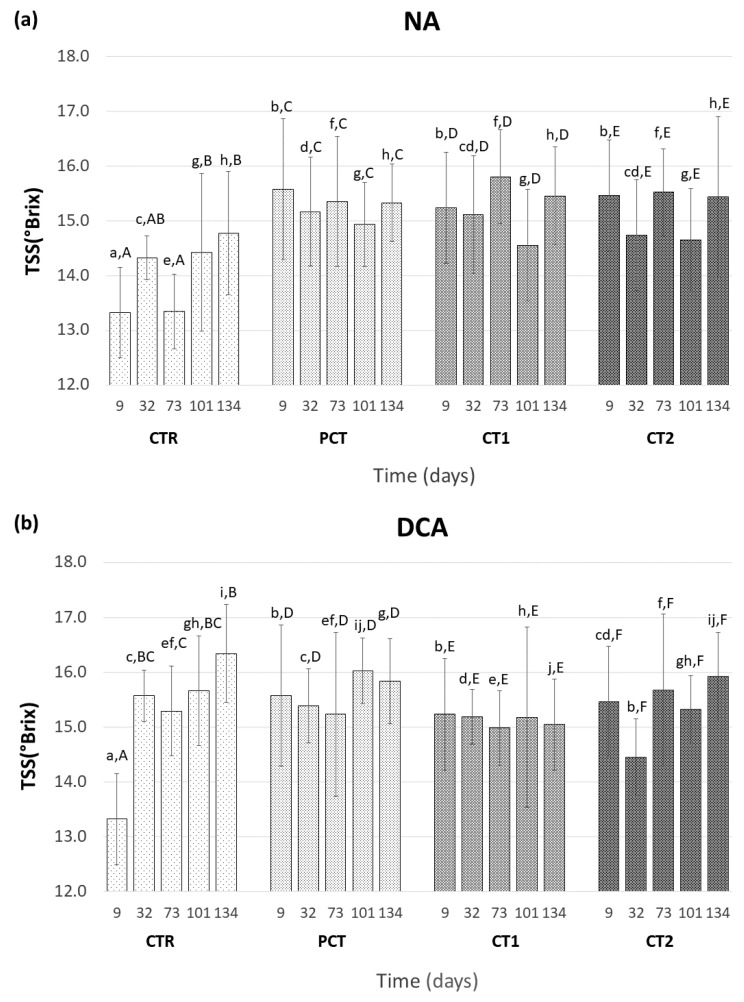
Evolution of total soluble solids (TSS) of “Rocha” pears at 9, 32, 73, 101, and 134 days. (**a**) NA (normal atmosphere); (**b**) DCA (dynamic controlled atmosphere) storage conditions. For each type of storage, four coating conditions are presented as follows: CTR (control), PCT (pectin), CT1 (coating 1), and CT2 (coating 2). Values are mean ± SD of ten replicates. The values followed by the same lowercase letter indicate no significant differences at the same sampling time between storage conditions, and by the same uppercase letter indicate no significant differences between sampling times within each coating condition (LSD test at *p* < 0.05).

TSS are a maturity indicator commonly used in fruits and are measured to evaluate an eventual impact exerted by coatings on fruit ripening. The TSS content in each sampling point of each coating condition is presented for both types of storage, NA (Figure 4a) and DCA (Figure 4b). TSS content observed between storage conditions behaved similarly, which suggests no impact caused by this factor. Within each type of storage, the type of coating (PCT, CT1, or CT2) also did not have an impact on fruit TSS content, because no significant differences between them were observed. In these conditions, is also possible to observe that TSS remained stable along the assay period ranging between 14.45 and 16.02 °Brix. CTR was the only condition where significant differences were observed, particularly in the beginning of the assay, with a lower TSS content as compared with coated conditions.

**Figure 5:**
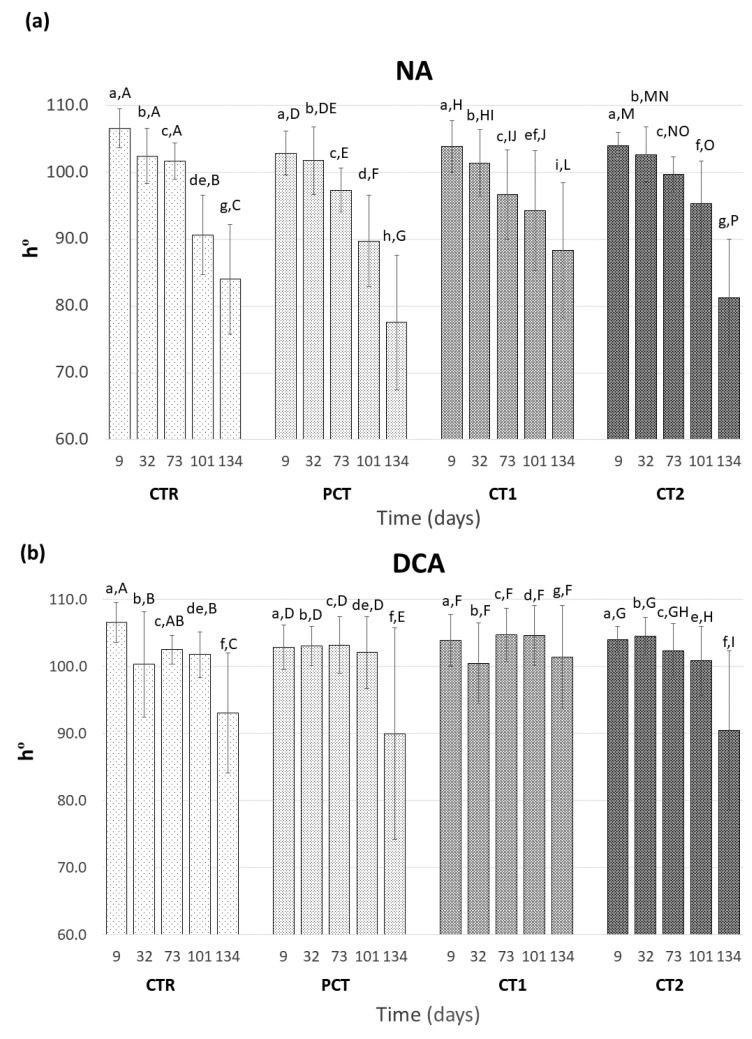
Evolution of the hue angle (h°) of “Rocha” pear at 9, 32, 73, 101, and 134 days. (**a**) NA (normal atmosphere); (**b**) DCA (dynamic controlled atmosphere) storage conditions. For each type of storage, four coating conditions are presented as follows: CTR (control), PCT (pectin), CT1 (coating 1), and CT2 (coating 2). Values are mean ± SD of ten replicates. The values followed by the same lowercase letter indicate no significant differences at the same sampling time between storage conditions, and by the same uppercase letter indicate no significant differences between sampling times within each coating condition (LSD test at *p* < 0.05).

Hue angle is considered to be a reliable measure of color changes in “Rocha” pears, since a decrease of this color coordinate reflects ripening-related yellowing of pear skin [46]. A hue of pure green is 180° and pure yellow corresponds to 90°. This parameter was measured throughout the assay for both NA (Figure 5a) and DCA (Figure 5b) storage conditions. In NA storage, the hue angle values ranged between 106.6° and 77.5° and a significant decrease was observed in all conditions indicating an increased yellowing of the fruits during storage. However, no significant differences between coating conditions were observed until 101 days of storage, which suggests little influence of the coatings in the yellowing of pear skin. A lower variation of hue angle was observed in DCA storage (maximum and minimum value of 106.6° and 89.9°, respectively), which indicates an increased retention of the green coloration on the pear skin. In fact, a significant decrease of hue angle was mainly observed after 134 days except for CT1 that remained stable throughout the entire assay.

#### 3.3.2. Accumulation of Conjugated Trienols (CTs)

**Figure 6:**
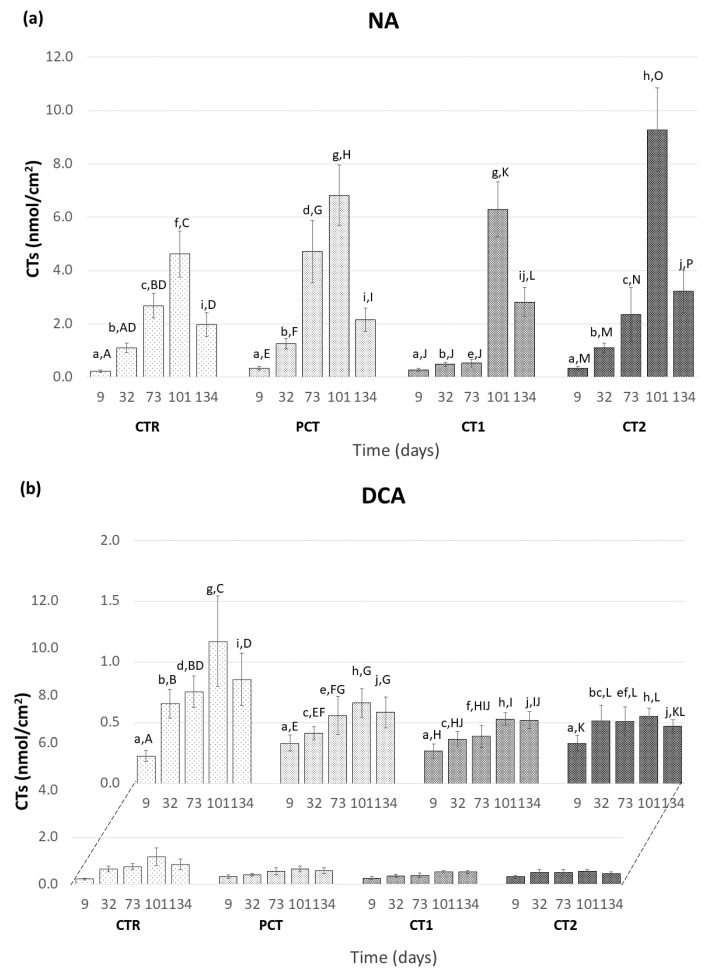
Evolution of conjugated trienols (CTs) content in the peel of “Rocha” pear. (**a**) NA (normal atmosphere); (**b**) DCA (dynamic controlled atmosphere) storage conditions. For each atmospheric storage, four coating conditions are presented as follows: CTR (control), PCT (pectin), CT1 (coating 1), and CT2 (coating 2). Values are mean ± SD of five replicates. The values followed by the same lowercase letter indicate no significant differences at the same sampling time between storage conditions, and by the same uppercase letter indicate no significant differences between sampling times within each coating condition (LSD test at *p* < 0.05).

The evolution of the CTs content in the pear peel was monitored throughout the storage assay and the results obtained for the NA and DCA storage conditions are shown in Figure 6a,b, respectively. These compounds, resulting from the in vivo oxidation of α-farnesene, are generally associated with the appearance of SC in pears, although critical values responsible for causing SC were never defined [47]. As such, and according to other studies, it is expected that their content would increase over storage [5]. As seen in Figure 6, such a trend is observed in both storage conditions, although the maximum CTs content reached in the two atmospheres are considerably different. In NA, the maximum value of CTs verified was 9.27 ± 1.57 nmol/cm^2^ (*t* = 101 days) in the samples treated with the CT2, whereas, in the DCA, the maximum is reached also at the end of 101 days and, in the CTR condition (1.17 ± 0.37 nmol/cm^2^). This difference is in line with what would be expected given the positive effect of the DCA storage conditions already reported in the literature concerning the prevention of the oxidation of α-farnesene into CTs [8].

Within NA conditions (Figure 6a), it is possible to observe that the CTR, PCT, and CT2 conditions behave similarly with an increase of CTs up to 101 days and a subsequent decrease in the last sampling point. CT1 shows a less accentuated growth until day 73, suggesting a delay in the synthesis of CTs in the pear skin and this treatment has a potential positive effect at preventing SC appearance in NA storage conditions. In DCA storage (Figure 6b), the coated conditions have a similar behavior between them but differ from the CTR by presenting a lower CTs content from day 32 until the end of the assay. This observation indicates a positive effect of the presence of the coating on CTs content when stored at DCA.

#### 3.3.3. Volatile Metabolites Analysis

**Figure 7:**
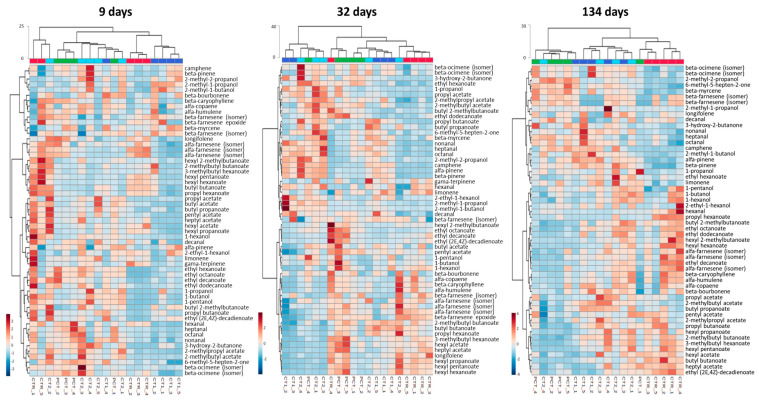
HCA heatmap visualization of the 57 selected compounds at 9, 32, and 134 days in NA (normal atmosphere) storage conditions. Euclidean distances are included on the dendrogram Y-axis. The relative content of each compound, illustrated through a chromatic scale (from low (dark blue) to high chromatographic area (dark red black)), corresponds to its peak area normalized by autoscaling. 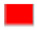 CTR (control); 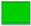 PCT (pectin); 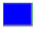 CT1 (coating 1); 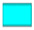 CT2 (coating 2).

**Figure 8:**
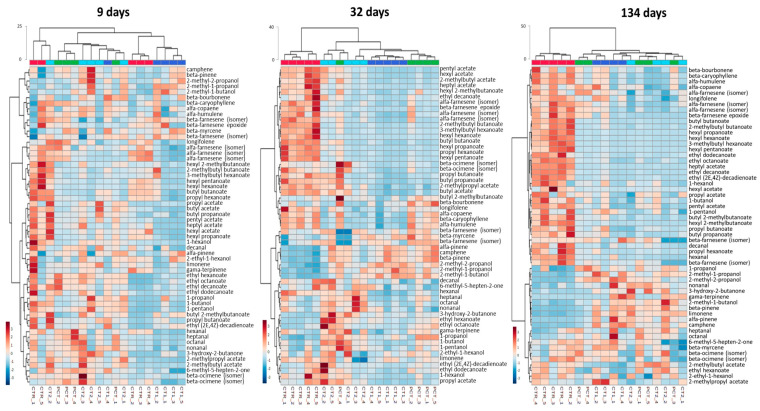
HCA heatmap visualization of the 57 selected compounds at 9, 32, and 134 days in DCA (dynamic controlled atmosphere) storage conditions. Euclidean distances are included on the dendrogram Y-axis. The relative content of each compound, illustrated through a chromatic scale (from low (dark blue) to high chromatographic area (dark red black)), corresponds to its peak area normalized by autoscaling. 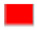 CTR (control); 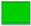 PCT (pectin); 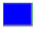 CT1 (coating 1); 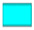 CT2 (coating 2).

In order to make a more comprehensive analysis of the evolution of the volatile compounds profile emitted by coated pears during the assay, a subsequent HCA was performed with the complete dataset comprising the 57 compounds identified (Table 1) for both NA (Figure 7) and DCA (Figure 8). This attempts to evaluate the impact of plant-based coatings on potential aroma and/or ripening status related compounds on “Rocha” pears throughout the storage. Regarding NA (Figure 7), HCA shows that over the storage period, a clear clustering of any coating condition is not observed. Replicates of each condition are dispersed and a clear distinction between them is not observed.

In the DCA storage (Figure 8), clustering formation along storage is more evident. At nine days, no clustering is observed and each condition samples are dispersed. However, at 32 days, two main branches are formed, one corresponding to CTR samples, and the other containing the coated conditions clustered according to the type of coating. The distinction of these two branches is further increased at 134 days through a higher Euclidean distance. Additionally, from day 32 to 134, coated samples became more similar among them from day 32 to day 134, although no clustering according to the type of coating is observed. The relative content of chemical families between CTR and coated conditions has a similar behavior to the NA storage, however, is more evident in this case. Coated samples have a lower content of esters and sesquiterpenes, while monoterpenes contents appear to be increased as compared with CTR.

**Figure 9:**
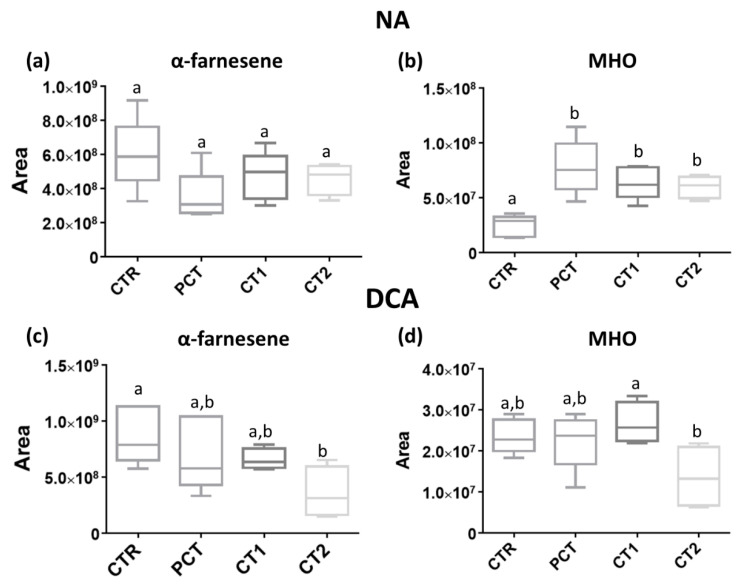
Boxplot representation of absolute areas of α-farnesene and 6-methyl-5-hepten-2-one (MHO) at day 134. (**a**,**b**) NA (normal atmosphere); (**c**,**d**) DCA (dynamic controlled atmosphere) storage conditions. For each storage, four coating conditions are presented as follows: CTR (control), PCT (pectin), CT1 (coating 1), and CT2 (coating 2). Each boxplot represents the areas of five replicates. The same lowercase letter indicates no significant differences between conditions (*p* < 0.05).

The content of specific volatile compounds (α-farnesene and MHO) associated with oxidative processes, namely SC, between coating conditions at day 134, is presented in Figure 9. For NA, although no significant differences are observed in α-farnesene content (Figure 9a) between the four coating conditions, the MHO content (Figure 9b) is significantly increased in all coated conditions as compared with the control. In DCA, the only coating that presents a lower α-farnesene content (Figure 9c) is CT2 as compared with the control. In this kind of storage, MHO content (Figure 9d) only differs between CT1 and CT2 where the latter is inferior.

## 4. Discussion

The chromatographic approach applied in this study was able to successfully contribute to the elucidation of the “Rocha” cultivar volatile profile. Overall, a high chromatographic resolution was achieved while maintaining a good spectral quality for trace peaks. As in most fruits, volatile profiles in pears are mainly constituted of aliphatic esters and it has been shown that their concentrations in different cultivars determined their organoleptic attributes and influence consumers preferences [43]. Most volatile compounds detected in “Rocha” pear headspace were in accordance with other studies of volatiles compounds released by European pears, in which acetate and butanoate esters were the most abundant components of this family [48,49]. However, α-fanesene represented 44% of total released compounds reported here, which was a value considerably higher than the one previously reported by Avelar et al. (1.21%) on ripe “Rocha” pears. Noteably, α-fanesene is a characteristic compound of pome fruits known to accumulate in pear skin across storage [50]. Since the pears analyzed in this study had already been stored for six months, high levels of α-farnesene were expected, and therefore explained the reported results.

On the basis of the several parameters analyzed across the four-month pilot scale storage assay, it is possible to observe a distinct impact of coating application between NA and DCA conditions. Overall, the presence of coatings in NA storage had little or no impact as compared with the uncoated pears. Regarding TSS, previous studies have demonstrated a similar behavior in which coatings had no impact on this quality parameter, although an increase was observed along the initial months of storage due to the conversion of starch into soluble sugars [13,51]. In the present study, once coatings were applied to pears after six months of storage, they had most likely already reached their maximum TSS content, hence, a constant TSS content was observed across the assay. Hue angle was also slightly affected by coatings in NA storage. Maintenance of hue color between control and all coated pears right after coating application is indeed considered to be an advantage of these types of coatings, since it demonstrates their optical clarity, and thus does not have a negative visual impact on treated fruits. However, the slight difference of hue angle in control and coated pears indicates that coatings were not capable of slowing down the ripening process. This absence of a clear preservation increase under NA storage was also visible in the volatile compounds emitted by pears. The HCA analysis of the whole dataset of identified compounds did not exhibit a clear clustering in coating conditions or the control, and there were no clear differences in relative amounts of chemical families among them. CTs evolution also corroborates other parameters’ behavior, since coatings were not able to prevent their accumulation except for CT1, where a slight delay was observed possibly due to a superior antioxidant potential of this extract. Overall, in this kind of storage, although desirable characteristics were maintained using coatings, an increased preservation and prevention of SC was not observed, possibly due to a low antioxidant activity of the extracts used.

Coated pears stored under DCA suffered different effects as compared with NA. Although the TSS content did not suffer an impact due to the coating presence or the type of storage, in DCA storage, tested conditions were able to delay yellowing of the fruit. Since this was also verified in the control condition, this effect was attributed to the type of storage, which has been previously reported to delay the yellowing of pear skin as compared with NA [52]. In DCA, released volatiles and CTs accumulation were found to be affected due to the presence of coatings. However, as in NA storage, no clear effect of the type of coating was observed, which shows that the extracts used do not exert a significant antioxidant effect on pears. Differences observed between coated and uncoated fruits could be due to the additional barrier that coatings represent to fruit respiration [53]. Since DCA storage imposes a low O_2_ atmosphere, coating presence could be further limiting O_2_ availability to the fruit. This limitation on fruit respiration could be responsible for volatile differences on coated fruits as compared with the control. Ultra-low oxygen storage (0.8 kPa O_2_) applied to ”Bartlett’ pears has been shown to suppress synthesis of esters as well as most aroma volatiles [21], which was in agreement with this study. Although several pear cultivars can be susceptible to lower *p*O_2_, “Rocha” pear has shown a very high storage capacity at around 0.5 kPa during 4.5 months of storage [54]. Furthermore, “Rocha” pear was also successfully stored for 8.5 months at static 0.5 kPa O_2_ without occurrence of storage disorders and maintained adequate fruit quality after 7 d of shelf-life [55].

Although levels of α-farnesene and MHO do not show a significant difference between the control and coated conditions at the end of the storage, the CTs content were found to be inferior for coated conditions from day 32 until the end of the assay. At day 134, the CTs content observed was reduced by 31.8%, 38.8%, and 44.7% for PCT, CT1, and CT2, respectively, which indicated a possible protective effect of the coatings against SC. This was in agreement with a previous study that demonstrated that low temperature and low oxygen levels were able to reduce the degradation of α-farnesene [56].

## 5. Conclusions

HS-SPME/GC×GC-ToFMS allowed us to detect hundreds of instrumental features, among which, a set of 57 compounds potentially related with aroma and/or ripening status of “Rocha” pears. Monitorization of such compounds over a four-month storage assay showed that coating application had an impact on their release under DCA storage but not in NA conditions. In the former, coatings decreased the release of “Rocha” pear volatiles potentially related with aroma or ripening status, namely, esters and sesquiterpenes, and showed a lower CTs content as compared with the control. The TSS content and color were not affected by coatings. These results indicate that the combination of pectin coatings with DCA storage can delay fruit ripening and prevent SC development, thus, contributing to the preservation of “Rocha” pears for longer periods. Impacts observed are mainly attributed to the coating as a gas barrier in the surface of the fruit, since coatings enriched with plant extracts had a similar behavior to PCT coating on measured parameters.

## Figures and Tables

**Table 1:** List of the 57 selected compounds of the “Rocha” pear volatile profile, using HS-SPME/GC×GC-ToFMS, including relevant chromatographic data used to assess compounds identification and respective odor descriptors. More details, including chromatographic data, are available in Table S1.

^1^*t*_R_ (s) ^a^	^2^*t*_R_ (s) ^a^	Compound	Formula	CAS Number	RI_Calc._^b^	RI_Lit_^c^	Odor Descriptor	Ref. ^d^
**Alcohols**								
75	0.85	1-propanol	C_3_H_8_O	71-23-8	590	591	oxidized pear, aldehyde	[39]
85	1.04	**2-methyl-1-propanol**	C_4_H_10_O	78-83-1	614	615	wine	[40]
95	1.38	**1-butanol**	C_4_H_10_O	71-36-3	638	637	medicinal, metallic	[6,39]
125	1.80	**2-methyl-1-butanol**	C_5_H_12_O	1565-80-6	710	728	acidic, sharp, spicy	[41]
145	2.26	**1-pentanol**	C_5_H_12_O	71-41-0	758	754	roasted	[6]
230	1.34	2-methyl-2-propanol	C_4_H_10_O	75-65-0	855	-	-	-
250	3.43	**1-hexanol**	C_6_H_14_O	111-27-3	873	877	oxidized, soapy, fresh rose, fresh, grass, engine	[6,39]
560	3.56	**2-ethyl-1-hexanol**	C_8_H_18_O	104-76-7	1036	1036	oily, sweet, floral	[40]
**Aldehydes**								
170	0.91	**Hexanal**	C_6_H_12_O	66-25-1	804	801	fruity, green	[6,40]
295	1.18	heptanal	C_7_H_14_O	111-71-7	905	906	fatty	[40]
490	1.40	octanal	C_8_H_16_O	124-13-0	1006	1005	fruity, orange	[42]
730	1.52	nonanal	C_9_H_18_O	124-19-6	1104	1106	orange, fresh rose, fatty	[40,42]
995	1.54	decanal	C_10_H_20_O	112-31-2	1206	1207	sweet, waxy, floral, citrus, fatty	[40]
**Ketones**								
115	2.84	**3-hydroxy-2-butanone**	C_4_H_8_O_2_	513-86-0	689	697	woody, yogurt	[40]
460	1.89	6-methyl-5-hepten-2-one	C_8_H_14_O	110-93-0	993	990	fatty, green, citrus	[40]
**Esters**								
115	0.63	**propyl acetate**	C_5_H_12_O_2_	109-60-4	683	684	floral, estery	[39]
150	0.71	**2-methylpropyl acetate**	C_6_H_12_O_2_	110-19-0	766	769	pear, apple, fruity, sweet, floral	[39]
185	0.87	**butyl acetate**	C_6_H_12_O_2_	123-86-4	817	819	fruity, pear, floral, sweet	[20,39,43]
265	0.93	2-methylbutyl acetate	C_7_H_14_O_2_	624-41-9	884	879	apple peel, banana	[40]
295	0.90	propyl butanoate	C_7_H_14_O_2_	105-66-8	905	900	pineapple, apricot, rancid, sweaty	[40]
310	0.95	butyl propanoate	C_7_H_14_O_2_	590-01-2	913	912	earthy, sweet	[40]
320	1.08	**pentyl acetate**	C_7_H_14_O_2_	628-63-7	918	919	pear, fruity, estery, sweet, candy, floral	[39]
475	1.06	butyl butanoate	C_8_H_16_O_2_	109-21-7	1000	996	pear, estery	[39]
490	1.10	**ethyl hexanoate**	C_8_H_16_O_2_	123-66-0	1006	1001	pear, floral, fruity, estery, sweet, cooked pear, oxidized, medicine	[39]
520	1.33	**hexyl acetate**	C_8_H_16_O_2_	142-92-7	1018	1024	pear, floral, sweet, fruity, estery	[39]
585	0.95	butyl 2-methylbutanoate	C_9_H_18_O_2_	15706-73-7	1045	1048	fruity, cocoa	[40]
625	1.03	2-methylbutyl butanoate	C_9_H_18_O_2_	51115-64-1	1061	-	-	-
715	1.12	propyl hexanoate	C_9_H_18_O_2_	626-77-7	1098	1098	ether, pineapple, blackberry	[40]
745	1.17	hexyl propanoate	C_9_H_18_O_2_	2445-76-3	1110	1114	earthy	[40]
765	1.32	**heptyl acetate**	C_9_H_18_O_2_	112-06-1	1117	1118	fermented	[6]
980	1.22	ethyl octanoate	C_10_H_20_O_2_	106-32-1	1200	1193	floral, sweet, cooked apple, fruity	[39]
1080	1.03	hexyl 2-methylbutanoate	C_11_H_22_O_2_	10032-15-2	1239	1236	green, fruity	[40]
1120	1.08	3-methylbutyl hexanoate	C_11_H_22_O_2_	2198-61-0	1255	1259	apple, pineapple, fruity, green, sweet	[40]
1215	1.14	hexyl pentanoate	C_11_H_22_O_2_	1117-59-5	1292	1293	-	-
1455	1.15	hexyl hexanoate	C_12_H_24_O_2_	6378-65-0	1388	1392	floral, candy	[39]
1485	1.23	ethyl decanoate	C_12_H_24_O_2_	110-38-3	1400	1391	fermented food	[39]
1660	1.89	ethyl (2E,4Z)-decadienoate	C_12_H_20_O_2_	3025-30-7	1475	-	pear	[39,43]
1945	1.20	ethyl dodecanoate	C_14_H_28_O	106-33-2	1600	1601	floral, fruity	[40]
**Monoterpenes**								
340	0.59	α-pinene	C_10_H_16_	80-56-8	929	932	pine, turpentine	[40]
365	0.66	camphene	C_10_H_16_	79-92-5	942	959	oily, camphor	[44]
420	0.72	β-pinene	C_10_H_16_	127-91-3	971	982	green, turpentine	[40]
460	0.85	β-myrcene	C_10_H_16_	123-35-3	992	994	sweet, balsamic, plastic	[40]
535	0.89	**Limonene**	C_10_H_16_	138-86-3	1024	1027	lemon, camphor, turpentine	[40]
570	0.97	β-ocimene (isomer)	C_10_H_16_	13877-91-3	1039	1042	herbaceous	[40]
595	1.00	β-ocimene (isomer)	C_10_H_16_	13877-91-3	1049	1048	herbaceous	[40]
610	0.96	γ-terpinene	C_10_H_16_	99-85-4	1055	1058	woody, terpene, tropical, lemon	[40]
**Sesquiterpenes**								
1400	0.90	α-copaene	C_15_H_24_	3856-25-5	1366	1370	-	-
1420	0.93	β-bourbonene	C_15_H_24_	5208-59-3	1374	1379	-	-
1460	1.04	longifolene	C_15_H_24_	475-20-7	1390	1395	-	-
1500	1.06	β-caryophyllene	C_15_H_24_	87-44-5	1406	1416	terpene, clove, turpentine	[40]
1560	1.07	β-farnesene (isomer)	C_15_H_24_	18794-84-8	1432	-	citrus, herbaceous	[40]
1585	1.17	α-humulene	C_15_H_24_	6753-98-6	1443	1450	-	-
1620	1.10	β-farnesene (isomer)	C_15_H_24_	18794-84-8	1457	1455	citrus, herbaceous	[40]
1720	1.18	**α-farnesene (isomer)**	C_15_H_24_	502-61-4	1500	-	flowery, balsam	[45]
1740	1.23	**α-farnesene (isomer)**	C_15_H_24_	502-61-4	1509	1505	flowery, balsam	[45]
1785	1.28	**α-farnesene (isomer)**	C_15_H_24_	502-61-4	1529	-	flowery, balsam	[45]
1995	2.00	β-farnesene epoxide	C_15_H_24_O	83637-40-5	1624	-	-	

^a^ Retention times for first (^1^*t*_R_) and second (^2^*t*_R_) dimensions in seconds. ^b^ Retention Index obtained through the modulated chromatogram. ^c^ Retention Index reported in the literature for Equity-5 GC column or equivalent. ^d^ Literature reference of odor descriptor. Volatile compounds previously reported in “Rocha” pear are highlighted in bold.

## Supplementary Materials

The following are available online at https://www.mdpi.com/2304-8158/9/9/1299/s1, Table S1: Full dataset used for statistics processing of the identified “Rocha” pear volatile compounds using HS-SPME/GC×GC-ToFMS, including relevant chromatographic data used to assess compounds identification and respective odor descriptors, Figure S1: Dendrograms of the hierarchical clustering analysis (HCA) for previously reported volatile compounds of “Rocha” pears at 9, 32, and 134 days of the assay in NA (normal atmosphere) storage conditions. Euclidean distances are included on the dendrogram Y-axis. The relative content of each compound, illustrated through a chromatic scale (from low (dark blue) to high chromatographic area (dark red black)), corresponds to its peak area normalized by autoscaling.  CTR (control);  PCT (pectin);  CT1 (coating 1);  CT2 (coating 2). Figure S2: Dendrograms of the hierarchical clustering analysis (HCA) for previously reported volatile compounds of ‘Rocha’ pear at 9, 32 and 134 days of the assay in DCA (dynamic controlled atmosphere) storage conditions. Euclidean distances are included on the dendrogram Y-axis. The relative content of each compound, illustrated through a chromatic scale (from low (dark blue) to high chromatographic area (dark red black)), corresponds to its peak area normalized by autoscaling.  CTR (control);  PCT (pectin);  CT1 (coating 1);  CT2 (coating 2).
