# Supplementary material for: The Impact of Plant-Based Coatings in “ROCHA” Pear Preservation during Cold Storage: A Metabolomic Approach
Source: Foods. 2020 Sep 15;9(9):1299. doi: 10.3390/foods9091299 (PMC7555455; doi:10.3390/foods9091299)
Supplement: Supplementary file 1 [file foods-09-01299-s001.zip › foods-904747.pdf]

*foods*

Article

# The Impact of Plant-Based Coatings in "ROCHA" Pear Preservation during Cold Storage: A Metabolomic Approach

**Alexandre M. A. Fonseca [1,2], Cindy Dias [3], Ana L. Amaro [3], Nélson Isidoro [4], Manuela Pintado [3], Armando J. D. Silvestre [2] and Sílvia M. Rocha [1,*]**

1 LAQV-REQUIMTE, Department of Chemistry, University of Aveiro, 3810-193 Aveiro, Portugal; alexandrefonseca@ua.pt
2 CICECO, Department of Chemistry, University of Aveiro, 3810-193 Aveiro, Portugal; armsil@ua.pt
3 Escola Superior de Biotecnologia, CBQF-Centro de Biotecnologia e Química Fina–Laboratório Associado, Universidade Católica Portuguesa, Rua de Diogo Botelho, 1327, 4169-005 Porto, Portugal; cdias@porto.ucp.pt (C.D.); aamaro@porto.ucp.pt (A.L.A.); mpintado@porto.ucp.pt (M.P.)
4 Cooperativa Agrícola dos Fruticultores do Cadaval, CRL (COOPVAL), EN 115, Km 26 2550-108 Cadaval, Portugal; nelson.isidoro@coopval.com
* Correspondence: smrocha@ua.pt; Tel.: +351-234-401-524

Received: 3 August 2020; Accepted: 9 September 2020; Published: 15 September 2020

**Abstract:** Although new storage technologies have been emerging in recent years, preservation of pear (*Pyrus communis* L.) remains a challenge for suppliers. Maintenance of desired organoleptic properties throughout cold storage using non-chemical strategies has been investigated and the use of edible coatings has shown potential to delay fruit quality deterioration during cold storage. Thus, the objective of this study is to evaluate the impact of pectin coatings including plant extracts, in "Rocha" pear (*Pyrus communis* L. cv. Rocha) preservation. A four-month pilot scale assay was performed in both dynamic controlled atmosphere (DCA) (−0.5 °C, 0.5% $O_2$, and 0.4% $CO_2$) and normal atmospheric (NA) conditions (2 °C). For each storage condition, the following three coatings were tested: pectin (3% *w*/*v*) (PCT), pectin (3% *w*/*v*) + strawberry tree leaves extract (9.5 mg/mL) (CT1), and pectin (3% *w*/*v*) + apple pomace extract (16 mg/mL) (CT2). Volatile compounds, potentially related to aroma or ripening status of "Rocha" pear, were monitored alongside with conjugated trienols (CTs) and maturity parameters. The combination of DCA conditions and the application of pectin coatings were able to reduce the release of Rocha pear volatiles associated with ripening status, (particularly esters and sesquiterpenes), as well as reduce CTs, which could contribute to the preservation of Rocha pear for longer periods.

**Keywords:** "Rocha" pear; cold storage; plant-based coatings; GC×GC-ToFMS; conjugated trienols

## 1. Introduction

"Rocha" pear (*Pyrus communis* L. cv. Rocha), a DOP (denomination of protected origin) cultivar from the western region of Portugal, is the fourth pear cultivar in Europe and the main cultivar in Portugal, with an average production of 173,000 tons/year, contributing to significant revenues for the Portuguese economy (120–130 million euros per year) [1,2]. Although "Rocha" pear is harvested in August, the aim is to have it available to the consumers for as long as possible. To achieve this, postharvest quality of pears must be maintained across storage through the reduction of fruit metabolic processes without compromising some desired changes such as the development of aroma and taste. In addition to traditional cold storage at around 0 °C, the use of 1-methylcyclopropene (1-MCP) and methodologies that modify storage atmospheric conditions by applying static (controlled atmosphere,

CA) or dynamic (dynamic controlled atmosphere, DCA) partial pressures of oxygen ($pO_2$) and carbon dioxide ($pCO_2$) have been used to extend the storage life of pears [3]. However, an affordable, consistently reliable, and easily applied technology that preserves the organoleptic characteristics of pears for a prolonged period has yet to be achieved.

The main disadvantage of prolonged cold exposure is the development of postharvest disorders that are responsible for fruit losses [4]. In "Rocha" pears, scald symptoms usually appear after four months of cold storage without atmosphere control, and scald occurrence is highly dependent on preharvest factors [5]. Compounds such as the sesquiterpene α-farnesene, conjugated trienes, and 6-methyl-5-hepten-2-one (MHO) have been extensively associated with superficial scald (SC) development (a cold-derived disorder) [6]. As ethylene production is reduced, α-farnesene metabolism is reduced, accumulating at high levels in the external wax layer, due to its lipophilic characteristics [7,8]. Further autoxidation of α-farnesene to conjugated trienes and trienols is thought to be the main cause of SC [4,8,9]. Several authors have demonstrated the relationship between α-farnesene and conjugated trienes in pears along with storage in which the accumulation of these oxidation products is associated with the decrease of α-farnesene content [5,7]. Production of MHO, resulting from autoxidation of conjugated trienes and trienols, is thought to be an important aspect of SC development, although its role is not yet clear [8].

In recent years, the use of edible coatings, in particular, polysaccharide-based coatings (starch, chitosan, xanthan gum, pectin, cellulose derivatives, and alginate), has shown great potential to delay fruit quality deterioration during cold storage due to their potential to reduce respiration rates and create a gas barrier on the fruit surface [10–13]. In addition, coatings can be supplemented with food compatible (and ideally natural) antioxidants to delay and reduce oxidative-related disorders [14]. Such natural antioxidants have been researched by several sources [15] and can possibly scavenge oxidation-linked damages by binding free radicals, thus protecting fruits from oxidative process [16]. Sharma and Rao [17] reported that a xanthan gum-based edible coating incorporated with cinnamic acid led to a significant reduction of the oxidative browning process, which ultimately preserved the organoleptic characteristics of fresh-cut pears. Recently, Dias et al. [18] selected, amongst fifteen natural-based extracts with antioxidant properties, strawberry tree (leaves and branches), as well as apple pomace methanolic extracts, as the most promising mitigators of fruit browning in fresh-cut pears. Total phenolic content of strawberry tree extracts (leaves and branches) were 207.9 mg and 104.1 mg GAE/g extract, respectively, and 6.8 mg GAE/g extract for apple pomace. These plant-based extracts promoted the reduction in the $IC_{50}$ against polyphenoloxidase and peroxidase activities. This study highlights the opportunity of by-products and agricultural waste extracts as novel anti-browning agents.

Pear aroma, which is one of the most important attributes to consumer acceptance, has been shown to be influenced by storage conditions and postharvest treatments [19–22]. The volatile organic compounds (VOCs) that contribute to the fruit aroma are generated through metabolic pathways during ripening, harvest, postharvest, and storage and vary according to the cultivar [23]. "Rocha" pear aroma is mainly composed of esters, aldehydes, and alcohols that originate from amino acid and fatty acid metabolism during ripening. During maturation, the enzyme alcohol acyl-CoA transferase activity increases, combining alcohols and CoA derivatives of short to medium chain length fatty acids to form aldehydes, then alcohols and finally esters [23,24]. A previous study that analyzed the volatile composition of ripe Rocha pears identified a total of 25 compounds, belonging mainly to the mentioned families and showed that the flavor-important aliphatic esters, butyl acetate and hexyl acetate, were the main compounds, accounting for more than 60% of the total volatile production [25]. Gomes et al. [26] further showed that the levels of butyl acetate in "Rocha" pears were not affected by storage temperature (0–15 °C), while hexyl acetate was significantly higher at 0 °C, but could be severely affected by hypoxic atmospheres, which led to an increase in the ethanol and ethyl acetate levels.

During fruit postharvest storage, significant compositional changes occur that are species and cultivar dependent. Some of those alterations reflect the reconfiguration of fruit metabolism as a consequence of the abiotic and biotic stresses encountered during storage conditions. Metabolomic

platforms, which are able to provide a profile of small molecules in a biological system that reflects its biological status, offer the possibility to improve our knowledge about the molecular mechanisms underlying fruit senescence which are affected under commercial storage conditions and to optimize molecular mechanisms according to a specific species or cultivar [27].

Gas chromatography-mass spectrometry (GC-MS) has been used to study the composition of fresh fruits VOCs, and to study physiological aspects of fruit maturation [23]. However, with this technique, only a small part of VOCs has been identified [28]. Comprehensive two-dimensional gas chromatography (GC×GC) is one of the most powerful separation techniques for VOCs, for the simultaneous determination of both major and trace components. This technique includes an interface, i.e., modulator, that physically connects a primary and secondary column and operates by preserving the separation obtained in the first dimension (first column), while achieving additional separation in the second dimension [29]. Such configuration provides significant signal enhancement, achieves highly effective fingerprinting, and has the potential to provide information encrypted in complex patterns of volatiles regarding sample origin, technological signature, and aroma [5,28,30]. Hence, with a single analysis, this analytical method provides different molecular information, allowing a comprehensive investigation of the impact of several factors (cultivar, environment, cold storage, postharvest strategies applied to prolong fruit quality, among others), as well as quality control in fruits.

Accordingly, this work was undertaken as a pilot study to evaluate the impact and potential of pectin coatings in the preservation of "Rocha" pears during cold storage. Since physiological disorders and fruit senescence of pears during cold storage are more prevalent after four months of storage [5], this work focused only on the storage period when pears were more susceptible to be affected. The coatings tested consisted of pectin alone or pectin enriched with either strawberry tree leaves or apple pomace extracts and were applied after six months of storage. A pilot scale assay was performed in the subsequent four months of storage in both DCA (−0.5 °C, 0.5% $O_2$, and 0.4% $CO_2$) and normal atmosphere (NA) conditions (2 °C) and a metabolomic based approach was performed using an advance gas chromatographic technique (GC×GC-ToFMS, comprehensive two-dimensional gas chromatography combined with time of flight mass spectrometry). Effects on the accumulation of conjugated trienols (CTs) and maturity parameters were also evaluated.

## 2. Materials and Methods

### 2.1. Samples, Materials, and Reagents

Pear fruit (*Pyrus communis* L. cv. Rocha) were harvested at an optimal maturation stage (firmness = 4.56 ± 0.56 N and total soluble solids (TSS) = 12.97 ± 0.27) from a commercial orchard in Cadaval (N 39° 25′, W 8° 54′, 120 m), Portugal. Pears were transported immediately after harvest to a commercial packinghouse and hand-sorted to select undamaged fruit of uniform size.

Then, the fruit were stored in DCA conditions (−0.5 °C, 0.5% $O_2$, and 0.4% $CO_2$) until use. Fresh strawberry tree (*Arbutus unedo* L.) leaves were separated from branches and stored at −20 °C. Before extraction, leaves were freeze-dried and ground into powder (IKA A10 analytical grinder). Apple pomace was stored at −80 °C upon arrival and used directly for extraction after thawing.

HPLC-grade ethanol and *n*-hexane (99%) were supplied by Carlo Erba Reagents S.A.S (Val-de-Reuil Cedex, France). Food grade pectin from citrus peel was purchased from Sigma-Aldrich. Quartz glass microplate with 96 wells (300 µL) was purchased from Hëllma Analytics. The retention index probe (a series of C8 to C20 straight-chain alkanes, in *n*-hexane) was supplied by Fluka (Buchs, Switzerland). The solid-phase microextraction (SPME) holder for manual sampling and the fiber coating used were purchased from Supelco (Bellefonte, PA). The SPME device included a 1 cm StableFlex™ fused silica fiber, coated with partially cross-linked 50/30 µm divinylbenzene/Carboxen™/poly(dimethylsiloxane) (DVB/CAR/PDMS). The fiber presents a wide range capacity for adsorbing and absorbing compounds with different physicochemical properties, with molecular weights ranging from 40 to 275. According

to the manufacturer's recommendations, the SPME fiber was initially conditioned once at 270 °C for 60 min in the GC injector, and daily for 10 min at 250 °C.

### *2.2. Plant-Based Coating*

#### 2.2.1. Extracts Preparation

Extraction procedure was performed according to Dias et al. [18] with modifications. Each plant material (strawberry tree leaves or apple pomace) was consecutively extracted (*m*/*v*, 1:20) three times (with renewal of solvent between extractions) with an ethanol/water mixture, 70/30 (*v*/*v*) for 1 h, at 25 °C, under constant stirring. Then, the suspensions were vacuum filtered, and the EtOH removed in a rotary evaporator (Büchi rotavapor R-114). Finally, extracts were freeze-dried. Extraction yields obtained for strawberry tree leaves and apple pomace were 13.7 and 4.7 g extract/100 g of fresh weight, respectively.

#### 2.2.2. Coating Formulation

After extraction, the optimal concentration, considering solubility and color, was determined for each extract. For further analysis, concentration of 9.53 and 16 mg/mL were used for strawberry tree leaves and apple pomace, respectively. Pectin coating was prepared according to a method previously described, with modifications [31]. Coatings were prepared by dissolving pectin (3% *w*/*v*) in distilled water and heated at 60 °C while stirring until the solution became clear. After cooling down to room temperature, plant extracts were dissolved at the concentration previously optimized. The coatings prepared were the following: PCT (pectin at 3% *w*/*v*), CT1 (pectin at 3% *w/v* + strawberry tree (*Arbutus unedo* L.) leaves extract at 9.53 mg/mL), and CT2 (pectin at 3% *w/v* + apple pomace extract at 16 mg/mL).

### *2.3. Pilot-Scale Storage Assay*

The impact of the application of the developed plant-based coatings was evaluated in a pilot scale storage assay with a duration of 134 days, according to the experimental procedure shown in Figure 1. A total of 750 pears, which had been submitted to a prior six-month storage period, was randomly selected and used as sample for the pilot scale storage assay. Adding to a control condition of uncoated pears (CTR), samples were coated with PCT, CT1, and CT2 by submersion in coating solutions for 1 min. Afterwards, fruits were suspended to drain the excess solution on the their surface and left to dry at room temperature (23 °C), for 2 h. Pears of the 4 conditions were stored in both DCA (−0.5 °C, 0.5% $O_2$, and 0.4% $CO_2$), and NA (2 °C) conditions during a 4-month period. Randomly selected pears of the 4 conditions of each kind of storage were sampled at 5 different times (9, 32, 73, 101, and 134 days). The analyzed parameters were comprised of pear headspace volatile compounds (at 9, 32, and 134 days), ripening indicators (total soluble solids (TSS) and hue angle), and CTs quantification for all sampling times.

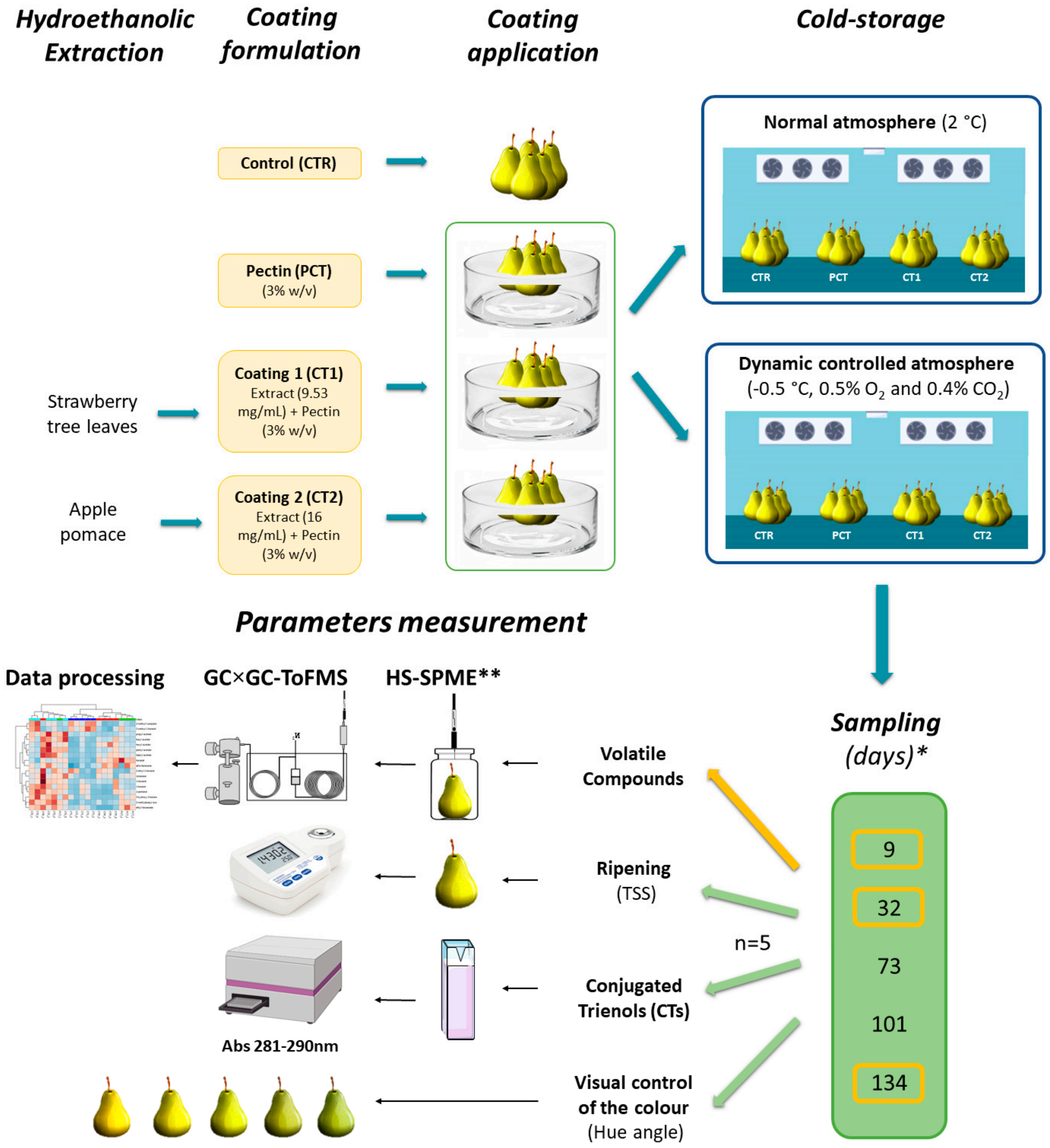


**Figure 1.** Schematic representation of the experimental design of the 4-month pilot-scale assay. Volatile compound analysis was performed for 9, 32, and 134 days, while conjugated trienols (CTs) determination, hue angle and total soluble solids (TSS) were performed for all available sampling times. * Days after coating application; ** after overnight pre-equilibrium.

### *2.4. Conjugated Trienols Analysis*

CTs' extraction and quantification were adapted from a previous publication [5]. For each pear, the peel was removed and immediately stored at −80 °C and only thawed before analysis. Peel disks (20) with a 5 mm diameter were excised from each sample and placed in 2 mL centrifuge tubes. Hexane (1.3 mL) was added to the peel disks and incubated at room temperature for 10 min. After incubation, the solvent was filtered through a sterile 0.20 µm cellulose acetate filter and transferred in triplicate into the quartz microplate. Absorbance at 291 and 280 nm was recorded using a BioTek Eon$^{TM}$ Microplate reader. CTs' concentrations were calculated using the molar extinction coefficients $\varepsilon_{281-290nm}$ = 25,000 and expressed as nmol/cm$^2$ [32]. Two-way analysis of variance (ANOVA) was performed to assess differences of CTs' contents using condition and time of storage as factors. Fisher's least significant

difference (LSD) was conducted for mean comparisons. Differences with a probability value of <0.05 were considered to be significant and all data were reported as mean ± SD. ANOVA analyses were done using STATISTICA software (StatSoft, v.8.0, Tulsa, OK, USA).

### 2.5. Assessment of Fruit Maturity

TSS, expressed as °Brix, were measured in the fruit juice by a digital refractometer PR1 ATAGO Co., Ltd. (Tokyo, Japan). Two-way ANOVA was performed to assess differences in the content of TSS using condition and time of storage as factors. Fisher's LSD was conducted for mean comparisons. Differences with a probability value of <0.05 were considered to be significant and all data were reported as mean ± SD (standard deviation). ANOVA analyses were done using STATISTICA software (StatSoft, v.8.0, Tulsa, OK, USA).

### 2.6. Surface Color Determination

Fruit surface color was measured with a CR-400 colorimeter (Konica Minolta, Osaka, Japan) using the D65 illuminant and the CIE (Commission Internationale de l'Eclairage) parameters (L*, a*, b*). Hue was calculated as hue angle (h° = arctan(b*/a*)). Two measurements were performed on opposite sides of the widest part of each fruit. Two-way ANOVA was performed to assess differences of the pilot-scale assay using treatment and days of storage as factors. Fisher's LSD was conducted for mean comparisons. Differences with a probability value of <0.05 were considered significant and all data were reported as mean ± SD. ANOVA analyses were done using STATISTICA software (StatSoft, v.8.0, Tulsa, OK, USA).

### 2.7. Volatile Profile Determination

#### 2.7.1. HS-SPME/GC×GC-ToFMS

GC×GC-ToFMS was employed to study in-depth the "Rocha" pear volatile profile variation across cold storage. Sampling, reporting of chemical analysis to data preprocessing, pretreatment, processing, and interpretation were performed according to the metabolomics standards initiative (MSI), as detailed below [33].

HS-SPME/GC×GC-ToFMS methodology was adapted from other studies [34,35] with optimization of the following parameters: time of pre-equilibrium and HS-SPME extraction, stationary phase of the columns, temperature ramp; modulation time, transfer line temperature, and drag gas flow. The effects of these parameters were evaluated based on visual analysis of the chromatograms through the chromatographic resolution, relative abundance of the principal peaks, and chromatogram overall structure.

Each analyzed pear was randomly selected and sealed in a 1 L airtight jar fitted with a rubber septum in the lid. Jars were closed and kept overnight at room temperature to reach equilibrium. Then, each jar was immersed in a water bath adjusted to 25.0 ± 0.1 °C and the DVB/CAR/PDMS SPME fiber was inserted in the headspace trough the septum, for 1 h. The volatiles adsorbed and absorbed on the SPME fiber coating were determined using a LECO Pegasus 4D GC×GC-ToFMS system (LECO, St. Joseph, MI, USA) consisting of an Agilent GC 7890A gas chromatograph (Agilent Technologies, Inc., Wilmington, DE, USA), with a dual stage jet cryogenic modulator (licensed from Zoex) and a secondary oven, and a mass spectrometer equipped with a ToF analyzer. After the extraction/concentration step, the SPME fiber was manually introduced into the port at 250 °C, for 3 min, for analytes desorption. The injection port was lined with a 0.75 mm I.D. glass liner. Splitless conditions (30 s) were used. An Equity-5 column (30 m × 0.32 mm I.D., 0.25 µm film thickness, Supelco, Bellefonte, PA, USA) was used as the first-dimension column ($^1$D) and a DB-FFAP column (0.79 m × 0.25 mm I.D., 0.25 µm film thickness, J&W Scientific Inc., Folsom, CA, USA) was used as the second-dimension column ($^2$D). The carrier gas was helium at a constant flow rate of 2.50 mL/min. The primary oven temperature was programmed from 40 °C (1 min) to 160 °C, at 3 °C/min, followed by a second ramp from 160 °C to

225 °C (2 min), at 15 °C/min. Secondary oven program was 5 °C offset above the primary one. The MS transfer line and MS source temperatures were both set at 250 °C. The modulation period was 5 s, keeping the modulator at 20 °C offset above primary oven, with hot and cold pulses of 0.90 and 1.60 s, respectively. The mass spectrometer ran in EI mode at 70 eV, using an *m*/*z* range of 35-300.

Total ion chromatograms were processed using the automated data processing software ChromaTOF® (LECO, St Joseph, MI, USA) at a signal-to-noise threshold of 100. Spectral deconvolution was computationally processed, being intended to reconstruct clean mass spectra for each component; whereas the GC×GC peak area was obtained by transforming the series of side-by-side second-dimension chromatograms into a two-dimensional chromatogram, the GC peak area being proportional to the generated signal intensity [36]. Contour plots were used to evaluate the general separation quality and for manual peak identification. For identification purposes, the mass spectrum of each detected metabolite was compared with mass spectral libraries, namely an in-house library of standards and two commercial databases (Wiley 275 and U.S. National Institute of Science and Technology (NIST) V. 2.0, Mainlib and Replib). A mass spectral match factor, similarity >700/1000, was used to decide whether a peak was correctly identified. Moreover, a manual analysis of mass spectra was performed, combining additional information such as the retention index (RI) value which was experimentally determined according to the van den Dool and Kratz equation [37]. A C8-C20 *n*-alkanes series was used for RI determination, and these values were compared with those reported in the literature for chromatographic columns similar to the above mentioned $^{1}$D column. The calculated retention index ($RI_{calc}$) only differed 0–5% as compared with the literature data ($RI_{lit}$). Five independent samples of each condition were analyzed.

2.7.2. Data Processing

A full data matrix consisting of 57 variables (metabolites) and 96 observations was constructed (Table S1, in Supplementary Materials). The 96 observations correspond to the 4 coating conditions of each type of storage at 3 times (9, 32, and 134 days), each one with 5 independent replicates. Using the MetaboAnalyst 3.0 (web interface) software (McGill University, Montreal, QC, Canada), autoscaling normalization of the data was applied and heatmap visualization was obtained on this matrix, using absolute GC peak area. Additionally, hierarchical clusters analysis (HCA) was also performed using the same software, to further examine the differences and similarities between each condition metabolite profiles. Ward's minimum variance algorithm method and squared Euclidean distances were employed.

## 3. Results

### *3.1. Pear Chromatogram Contour Plot Analysis*

A representative three-dimensional (3D) GC×G-ToFMS total ion chromatogram contour plot of "Rocha" pear is illustrated in Figure 2a. Here, it shows the pear headspace metabolite separation according to physicochemical characteristics, namely, through volatility (first dimension) and polarity (second dimension), when a non-polar and polar set of columns is used. In this way, a structured chromatogram is obtained, in which structurally related analytes occupy similar two-dimensional (2D) spaces. The GC×GC configuration also results in the resolution of many peaks that otherwise would be overlapped in a one-dimensional (1D) chromatogram.

An example of such advantage can be observed in Figure 2b. Analytes with similar volatility ($^{1}t_R$ – 460 s) such as MHO and β-myrcene can be effectively separated by the second column according to their polarity ($^{2}t_R$ of 1.894 and 0.850 s, respectively).

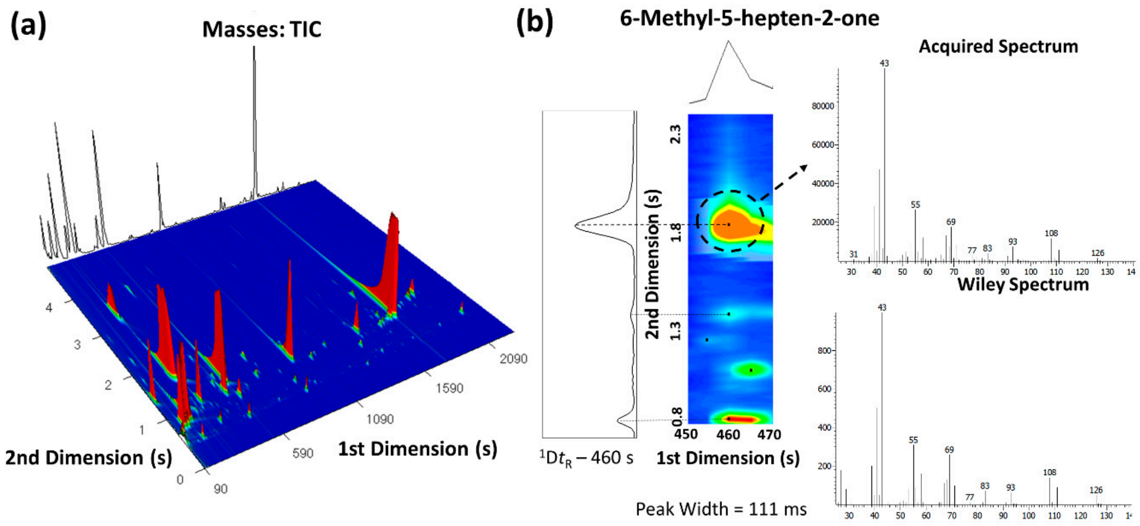


**Figure 2.** (**a**) Three-dimensional GC×GC total ion chromatogram plot of an uncoated pear at 9 days, used to illustrate the "Rocha" pear volatile profile; (**b**) Enlargement of a part of GC×GC chromatogram contour plot of the pear showing the separation of metabolites with the same retention time. The 111 milliseconds wide 6-methyl-5-hepten-2-one (MHO) GC×GC peak is easily defined and identified at a mass spectral acquisition of 125 spectra/s. Spectral quality at this high acquisition rate is maintained due to the ToFMS with continuous full-range mass spectral acquisition rate. As observed, the MHO mass spectrum is very similar (similarity value of 912/1000) as compared with the Wiley database.

From each pear analyzed, a chromatogram, with approximate 550 instrumental features, was obtained. These features were constituted mostly by minor peaks and a few major ones. Major peaks detected on uncoated pears at nine days were 1-butanol, 1-hexanol, butyl acetate, pentyl acetate, hexyl acetate, butyl butanoate. and α- and β-farnesene (Table S1). From the total instrumental features obtained, a total of 57 compounds were identified and selected for further analysis. Selected metabolites are listed in Table 1 along with the respective odor descriptors obtained from literature. Selected compounds were comprised of metabolites belonging to several chemical families commonly identified in pear volatile profiles, namely, alcohols, aldehydes, esters, and mono- and sesquiterpenes [6]. Such chemical families are known to be the major volatiles emitted by pears and the main contributors to the pear aroma [6]. Among these, straight-chain esters, alcohols, and aldehydes, which are generally regarded to be a result of fatty acid metabolism, are produced in pears during fruit development and maturation [38]. From the set of compounds selected, esters were qualitatively the most representative chemical family. Other compounds associated with SC development such as α-farnesene and MHO were also identified [6].

For the selected compounds, at the first sampling time of the pilot scale assay ($t$ = 9 days), uncoated pears' headspace volatiles were mainly composed of sesquiterpenes (71.5%), followed by esters (23.0%), alcohols (3.7%), monoterpenes (0.8%), aldehydes (0.6%), and ketones (0.3%). A previous study on the headspace volatiles of ripe "Rocha" pears identified a total of 25 compounds with butyl acetate and hexyl acetate accounting for 66.5% of total volatiles peak area [25]. A total of 17 out of these 25 reported volatile compounds were successfully identified in the present study and included in the metabolites to be analyzed (Table 1).

**Table 1.** List of the 57 selected compounds of the "Rocha" pear volatile profile, using HS-SPME/GC×GC-ToFMS, including relevant chromatographic data used to assess compounds identification and respective odor descriptors. More details, including chromatographic data, are available in Table S1.

| $^{1}t_R$ (s) [a] | $^{2}t_R$ (s) [a] | Compound | Formula | CAS Number | $RI_{Calc.}$ [b] | $RI_{Lit}$ [c] | Odor Descriptor | Ref. [d] |
|---|---|---|---|---|---|---|---|---|
| **Alcohols** | | | | | | | | |
| 75 | 0.85 | 1-propanol | $C_3H_8O$ | 71-23-8 | 590 | 591 | oxidized pear, aldehyde | [39] |
| 85 | 1.04 | **2-methyl-1-propanol** | $C_4H_{10}O$ | 78-83-1 | 614 | 615 | wine | [40] |
| 95 | 1.38 | **1-butanol** | $C_4H_{10}O$ | 71-36-3 | 638 | 637 | medicinal, metallic | [6,39] |
| 125 | 1.80 | **2-methyl-1-butanol** | $C_5H_{12}O$ | 1565-80-6 | 710 | 728 | acidic, sharp, spicy | [41] |
| 145 | 2.26 | **1-pentanol** | $C_5H_{12}O$ | 71-41-0 | 758 | 754 | roasted | [6] |
| 230 | 1.34 | 2-methyl-2-propanol | $C_4H_{10}O$ | 75-65-0 | 855 | - | - | - |
| 250 | 3.43 | **1-hexanol** | $C_6H_{14}O$ | 111-27-3 | 873 | 877 | oxidized, soapy, fresh rose, fresh, grass, engine | [6,39] |
| 560 | 3.56 | **2-ethyl-1-hexanol** | $C_8H_{18}O$ | 104-76-7 | 1036 | 1036 | oily, sweet, floral | [40] |
| **Aldehydes** | | | | | | | | |
| 170 | 0.91 | **Hexanal** | $C_6H_{12}O$ | 66-25-1 | 804 | 801 | fruity, green | [6,40] |
| 295 | 1.18 | heptanal | $C_7H_{14}O$ | 111-71-7 | 905 | 906 | fatty | [40] |
| 490 | 1.40 | octanal | $C_8H_{16}O$ | 124-13-0 | 1006 | 1005 | fruity, orange | [42] |
| 730 | 1.52 | nonanal | $C_9H_{18}O$ | 124-19-6 | 1104 | 1106 | orange, fresh rose, fatty | [40,42] |
| 995 | 1.54 | decanal | $C_{10}H_{20}O$ | 112-31-2 | 1206 | 1207 | sweet, waxy, floral, citrus, fatty | [40] |
| **Ketones** | | | | | | | | |
| 115 | 2.84 | **3-hydroxy-2-butanone** | $C_4H_8O_2$ | 513-86-0 | 689 | 697 | woody, yogurt | [40] |
| 460 | 1.89 | 6-methyl-5-hepten-2-one | $C_8H_{14}O$ | 110-93-0 | 993 | 990 | fatty, green, citrus | [40] |
| **Esters** | | | | | | | | |
| 115 | 0.63 | **propyl acetate** | $C_5H_{12}O_2$ | 109-60-4 | 683 | 684 | floral, estery | [39] |
| 150 | 0.71 | **2-methylpropyl acetate** | $C_6H_{12}O_2$ | 110-19-0 | 766 | 769 | pear, apple, fruity, sweet, floral | [39] |
| 185 | 0.87 | **butyl acetate** | $C_6H_{12}O_2$ | 123-86-4 | 817 | 819 | fruity, pear, floral, sweet | [20,39,43] |
| 265 | 0.93 | 2-methylbutyl acetate | $C_7H_{14}O_2$ | 624-41-9 | 884 | 879 | apple peel, banana | [40] |
| 295 | 0.90 | propyl butanoate | $C_7H_{14}O_2$ | 105-66-8 | 905 | 900 | pineapple, apricot, rancid, sweaty | [40] |
| 310 | 0.95 | butyl propanoate | $C_7H_{14}O_2$ | 590-01-2 | 913 | 912 | earthy, sweet | [40] |
| 320 | 1.08 | **pentyl acetate** | $C_7H_{14}O_2$ | 628-63-7 | 918 | 919 | pear, fruity, estery, sweet, candy, floral | [39] |
| 475 | 1.06 | butyl butanoate | $C_8H_{16}O_2$ | 109-21-7 | 1000 | 996 | pear, estery | [39] |
| 490 | 1.10 | **ethyl hexanoate** | $C_8H_{16}O_2$ | 123-66-0 | 1006 | 1001 | pear, floral, fruity, estery, sweet, cooked pear, oxidized, medicine | [39] |
| 520 | 1.33 | **hexyl acetate** | $C_8H_{16}O_2$ | 142-92-7 | 1018 | 1024 | pear, floral, sweet, fruity, estery | [39] |
| 585 | 0.95 | butyl 2-methylbutanoate | $C_9H_{18}O_2$ | 15706-73-7 | 1045 | 1048 | fruity, cocoa | [40] |
| 625 | 1.03 | 2-methylbutyl butanoate | $C_9H_{18}O_2$ | 51115-64-1 | 1061 | - | - | - |
| 715 | 1.12 | propyl hexanoate | $C_9H_{18}O_2$ | 626-77-7 | 1098 | 1098 | ether, pineapple, blackberry | [40] |
| 745 | 1.17 | hexyl propanoate | $C_9H_{18}O_2$ | 2445-76-3 | 1110 | 1114 | earthy | [40] |

**Table 1.** *Cont.*

| $^1t_R$ (s) [a] | $^2t_R$ (s) [a] | Compound | Formula | CAS Number | $RI_{Calc.}$ [b] | $RI_{Lit}$ [c] | Odor Descriptor | Ref. [d] |
|---|---|---|---|---|---|---|---|---|
| 765 | 1.32 | **heptyl acetate** | $C_9H_{18}O_2$ | 112-06-1 | 1117 | 1118 | fermented | [6] |
| 980 | 1.22 | ethyl octanoate | $C_{10}H_{20}O_2$ | 106-32-1 | 1200 | 1193 | floral, sweet, cooked apple, fruity | [39] |
| 1080 | 1.03 | hexyl 2-methylbutanoate | $C_{11}H_{22}O_2$ | 10032-15-2 | 1239 | 1236 | green, fruity | [40] |
| 1120 | 1.08 | 3-methylbutyl hexanoate | $C_{11}H_{22}O_2$ | 2198-61-0 | 1255 | 1259 | apple, pineapple, fruity, green, sweet | [40] |
| 1215 | 1.14 | hexyl pentanoate | $C_{11}H_{22}O_2$ | 1117-59-5 | 1292 | 1293 | - | - |
| 1455 | 1.15 | hexyl hexanoate | $C_{12}H_{24}O_2$ | 6378-65-0 | 1388 | 1392 | floral, candy | [39] |
| 1485 | 1.23 | ethyl decanoate | $C_{12}H_{24}O_2$ | 110-38-3 | 1400 | 1391 | fermented food | [39] |
| 1660 | 1.89 | ethyl (2E,4Z)-decadienoate | $C_{12}H_{20}O_2$ | 3025-30-7 | 1475 | - | pear | [39,43] |
| 1945 | 1.20 | ethyl dodecanoate | $C_{14}H_{28}O$ | 106-33-2 | 1600 | 1601 | floral, fruity | [40] |
| **Monoterpenes** | | | | | | | | |
| 340 | 0.59 | α-pinene | $C_{10}H_{16}$ | 80-56-8 | 929 | 932 | pine, turpentine | [40] |
| 365 | 0.66 | camphene | $C_{10}H_{16}$ | 79-92-5 | 942 | 959 | oily, camphor | [44] |
| 420 | 0.72 | β-pinene | $C_{10}H_{16}$ | 127-91-3 | 971 | 982 | green, turpentine | [40] |
| 460 | 0.85 | β-myrcene | $C_{10}H_{16}$ | 123-35-3 | 992 | 994 | sweet, balsamic, plastic | [40] |
| 535 | 0.89 | **Limonene** | $C_{10}H_{16}$ | 138-86-3 | 1024 | 1027 | lemon, camphor, turpentine | [40] |
| 570 | 0.97 | β-ocimene (isomer) | $C_{10}H_{16}$ | 13877-91-3 | 1039 | 1042 | herbaceous | [40] |
| 595 | 1.00 | β-ocimene (isomer) | $C_{10}H_{16}$ | 13877-91-3 | 1049 | 1048 | herbaceous | [40] |
| 610 | 0.96 | γ-terpinene | $C_{10}H_{16}$ | 99-85-4 | 1055 | 1058 | woody, terpene, tropical, lemon | [40] |
| **Sesquiterpenes** | | | | | | | | |
| 1400 | 0.90 | α-copaene | $C_{15}H_{24}$ | 3856-25-5 | 1366 | 1370 | - | - |
| 1420 | 0.93 | β-bourbonene | $C_{15}H_{24}$ | 5208-59-3 | 1374 | 1379 | - | - |
| 1460 | 1.04 | longifolene | $C_{15}H_{24}$ | 475-20-7 | 1390 | 1395 | - | - |
| 1500 | 1.06 | β-caryophyllene | $C_{15}H_{24}$ | 87-44-5 | 1406 | 1416 | terpene, clove, turpentine | [40] |
| 1560 | 1.07 | β-farnesene (isomer) | $C_{15}H_{24}$ | 18794-84-8 | 1432 | - | citrus, herbaceous | [40] |
| 1585 | 1.17 | α-humulene | $C_{15}H_{24}$ | 6753-98-6 | 1443 | 1450 | - | - |
| 1620 | 1.10 | β-farnesene (isomer) | $C_{15}H_{24}$ | 18794-84-8 | 1457 | 1455 | citrus, herbaceous | [40] |
| 1720 | 1.18 | **α-farnesene (isomer)** | $C_{15}H_{24}$ | 502-61-4 | 1500 | - | flowery, balsam | [45] |
| 1740 | 1.23 | **α-farnesene (isomer)** | $C_{15}H_{24}$ | 502-61-4 | 1509 | 1505 | flowery, balsam | [45] |
| 1785 | 1.28 | **α-farnesene (isomer)** | $C_{15}H_{24}$ | 502-61-4 | 1529 | - | flowery, balsam | [45] |
| 1995 | 2.00 | β-farnesene epoxide | $C_{15}H_{24}O$ | 83637-40-5 | 1624 | - | - | |

[a] Retention times for first ($^1t_R$) and second ($^2t_R$) dimensions in seconds. [b] Retention Index obtained through the modulated chromatogram. [c] Retention Index reported in the literature for Equity-5 GC column or equivalent. [d] Literature reference of odor descriptor. Volatile compounds previously reported in "Rocha" pear are highlighted in bold.

### 3.2. Impact of Plant-Based Coatings on Previously Reported Volatile Compounds of "Rocha" Pear

In a first approach, the impact of the coatings on headspace volatile compounds previously identified in "Rocha" pear (Table 1) was assessed by cluster analysis. This analysis was performed at three sampling times (9, 32, and 134 days) for both storage conditions. The results obtained from the HCA analysis are presented in Figure 3 with a heatmap visualization of relative amounts of the analytes for the last sampling point (134 days). Heatmaps for all sampling points are provided in hte Supplementary Materials for NA (Figure S1) and DCA (Figure S2) conditions.

As seen in Figure 3, at nine days after coating application, when storage conditions have yet exerted little influence on pear physiology, an almost complete clustering of replicates according to coating condition is visible in the dendrogram. CT2 was shown to be the most distinctive condition because it was placed in a different branch with a higher Euclidean distance. The branch containing the remaining coating conditions was divided among CT1 samples and the two remaining conditions (CTR and PCT). CTR and PCT were found to be the most similar, which is probably because no plant extract was incorporated in them.

In subsequent times of analysis, a different clustering behavior was observed between the two storage conditions. In the case of NA, dendrograms obtained do not exhibit a sample clustering that can be associated with the coating conditions. In this case, the data suggest that the coating application and the kind of plant extract incorporated in the coating do not have a relevant impact on the release of the selected analytes after 32 days of storage. Differences observed in this case are only attributed to sample variability.

Regarding storage in DCA conditions, a different behavior is observed. In this case, coated samples become increasingly distinct over time relative to the control. This is particularly evident at the end of 134 days, when all control samples are clustered in one of the two main branches of the dendrogram. On the one hand, the other main branch contains samples of coated conditions (PCT, CT1, and CT2) without any clustering according to the type of coating applied. These samples exhibit a low Euclidean distance, which indicates a high similarity between them and suggests that plant extract incorporation on coatings does not have a relevant impact on the release of the analyzed compounds to the pear headspace. On the other hand, coating application by itself seems to have an impact on the release of such compounds since CTR samples are clustered at a higher distance from the remaining samples. Through the heatmap visualization, it is possible to verify that this difference is explained mainly by a reduction of the release of esters, such as butyl acetate and hexyl acetate, which are the most abundant compounds released by "Rocha" pears. In addition, 2-methyl-1-propanol, 2-methyl-1-butanol, 3-hydroxy-2-butanone, and limonene release are increased in coated pears.

### 3.3. Impact of Plant-Based Coatings on "Rocha" Pear Physiology during Storage

Adding to the evaluation of the variations on the release of the known "Rocha" pear volatile compounds caused by plant-based coatings, other parameters were monitored to assess possible impacts on fruit physiology of such coatings during fruit storage. Fruit maturity was evaluated by measuring the TSS content on pear juice and hue angle of pear surface, while the development of SC was assessed through CTs determination in fruit peel. To conclude, a broader metabolomic approach using the complete dataset of volatile compounds identified (Table 1), comprising both aroma and ripening related compounds, was used to evaluate metabolic alterations on fruits.

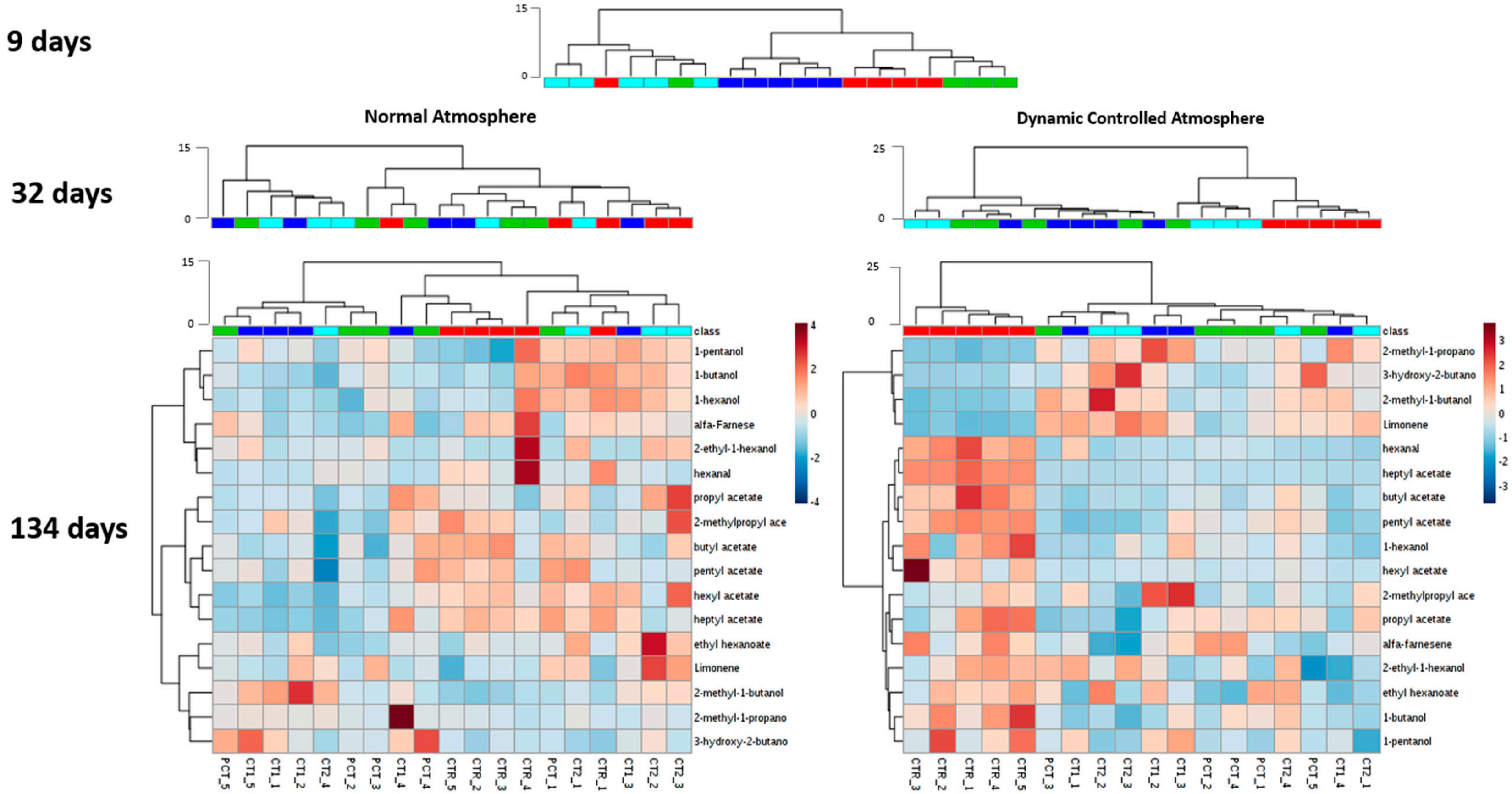


**Figure 3.** Dendrograms of the hierarchical clustering analysis (HCA) performed for previously reported volatile compounds of "Rocha" pear at 9, 32, and 134 days of the assay in both normal atmosphere (NA) and dynamic controlled atmosphere (DCA) storage conditions. Euclidean distances are included on the dendrogram *Y*-axis. Heatmap visualization at day 134 shows the relative content of each compound, illustrated through a chromatic scale (from low (dark blue) to high chromatographic area (dark red black)), which corresponds to its peak area normalized by autoscaling. CTR (control); PCT (pectin); CT1 (coating 1); CT2 (coating 2).

3.3.1. Fruit Maturity

TSS are a maturity indicator commonly used in fruits and are measured to evaluate an eventual impact exerted by coatings on fruit ripening. The TSS content in each sampling point of each coating condition is presented for both types of storage, NA (Figure 4a) and DCA (Figure 4b). TSS content observed between storage conditions behaved similarly, which suggests no impact caused by this factor. Within each type of storage, the type of coating (PCT, CT1, or CT2) also did not have an impact on fruit TSS content, because no significant differences between them were observed. In these conditions, is also possible to observe that TSS remained stable along the assay period ranging between 14.45 and 16.02 °Brix. CTR was the only condition where significant differences were observed, particularly in the beginning of the assay, with a lower TSS content as compared with coated conditions.

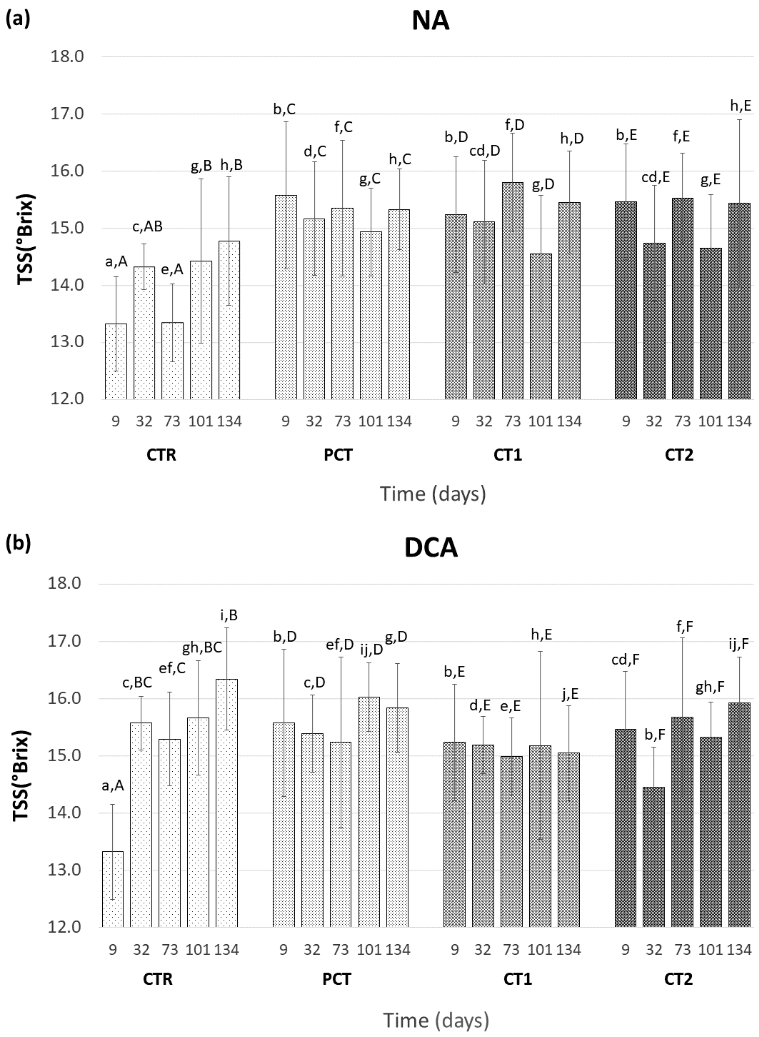


**Figure 4.** Evolution of total soluble solids (TSS) of "Rocha" pears at 9, 32, 73, 101, and 134 days. (**a**) NA (normal atmosphere); (**b**) DCA (dynamic controlled atmosphere) storage conditions. For each type of storage, four coating conditions are presented as follows: CTR (control), PCT (pectin), CT1 (coating 1), and CT2 (coating 2). Values are mean ± SD of ten replicates. The values followed by the same lowercase letter indicate no significant differences at the same sampling time between storage conditions, and by the same uppercase letter indicate no significant differences between sampling times within each coating condition (LSD test at $p < 0.05$).

Hue angle is considered to be a reliable measure of color changes in "Rocha" pears, since a decrease of this color coordinate reflects ripening-related yellowing of pear skin [46]. A hue of pure

green is 180° and pure yellow corresponds to 90°. This parameter was measured throughout the assay for both NA (Figure 5a) and DCA (Figure 5b) storage conditions. In NA storage, the hue angle values ranged between 106.6° and 77.5° and a significant decrease was observed in all conditions indicating an increased yellowing of the fruits during storage. However, no significant differences between coating conditions were observed until 101 days of storage, which suggests little influence of the coatings in the yellowing of pear skin. A lower variation of hue angle was observed in DCA storage (maximum and minimum value of 106.6° and 89.9°, respectively), which indicates an increased retention of the green coloration on the pear skin. In fact, a significant decrease of hue angle was mainly observed after 134 days except for CT1 that remained stable throughout the entire assay.

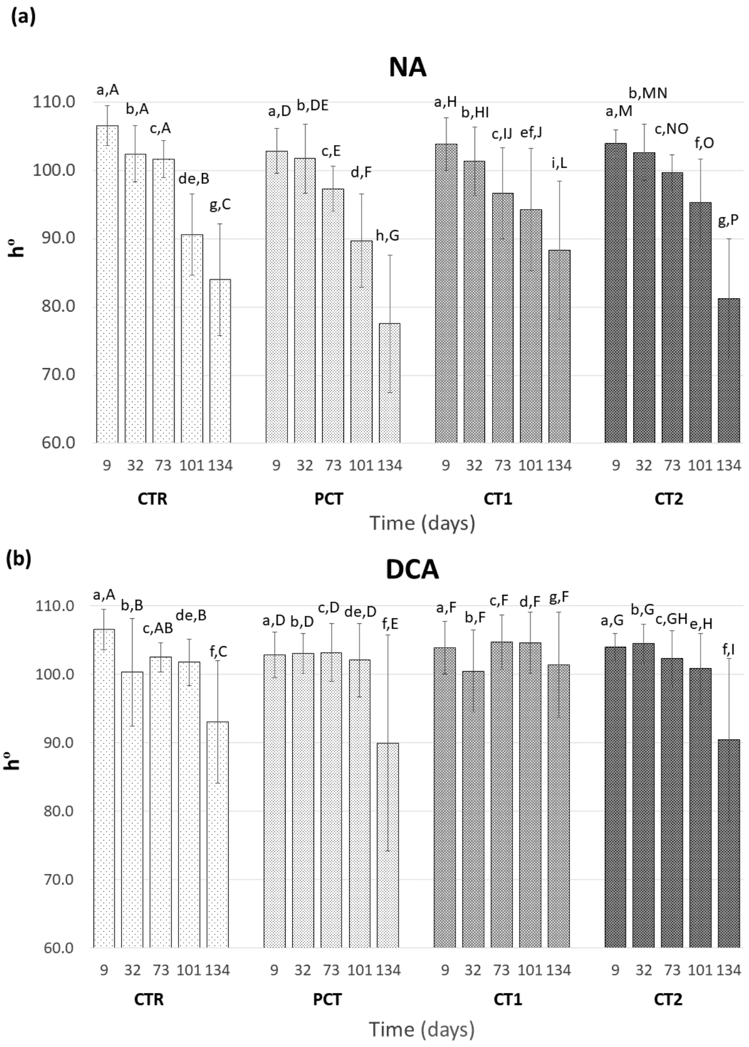


**Figure 5.** Evolution of the hue angle (h°) of "Rocha" pear at 9, 32, 73, 101, and 134 days. (**a**) NA (normal atmosphere); (**b**) DCA (dynamic controlled atmosphere) storage conditions. For each type of storage, four coating conditions are presented as follows: CTR (control), PCT (pectin), CT1 (coating 1), and CT2 (coating 2). Values are mean ± SD of ten replicates. The values followed by the same lowercase letter indicate no significant differences at the same sampling time between storage conditions, and by the same uppercase letter indicate no significant differences between sampling times within each coating condition (LSD test at $p < 0.05$).

### 3.3.2. Accumulation of Conjugated Trienols (CTs)

The evolution of the CTs content in the pear peel was monitored throughout the storage assay and the results obtained for the NA and DCA storage conditions are shown in Figure 6a,b, respectively.

These compounds, resulting from the in vivo oxidation of α-farnesene, are generally associated with the appearance of SC in pears, although critical values responsible for causing SC were never defined [47]. As such, and according to other studies, it is expected that their content would increase over storage [5]. As seen in Figure 6, such a trend is observed in both storage conditions, although the maximum CTs content reached in the two atmospheres are considerably different. In NA, the maximum value of CTs verified was 9.27 ± 1.57 nmol/cm$^2$ ($t$ = 101 days) in the samples treated with the CT2, whereas, in the DCA, the maximum is reached also at the end of 101 days and, in the CTR condition (1.17 ± 0.37 nmol/cm$^2$). This difference is in line with what would be expected given the positive effect of the DCA storage conditions already reported in the literature concerning the prevention of the oxidation of α-farnesene into CTs [8].

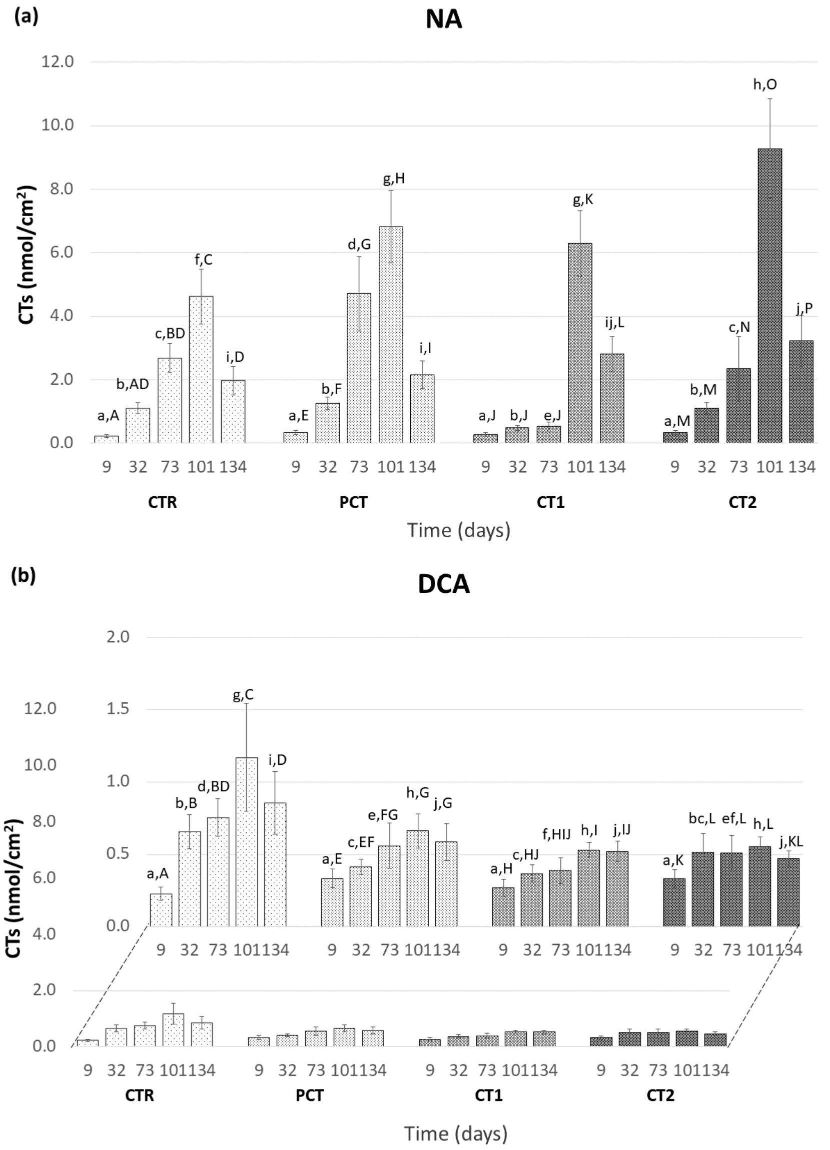


**Figure 6.** Evolution of conjugated trienols (CTs) content in the peel of "Rocha" pear. (**a**) NA (normal atmosphere); (**b**) DCA (dynamic controlled atmosphere) storage conditions. For each atmospheric storage, four coating conditions are presented as follows: CTR (control), PCT (pectin), CT1 (coating 1), and CT2 (coating 2). Values are mean ± SD of five replicates. The values followed by the same lowercase letter indicate no significant differences at the same sampling time between storage conditions, and by the same uppercase letter indicate no significant differences between sampling times within each coating condition (LSD test at $p < 0.05$).

Within NA conditions (Figure 6a), it is possible to observe that the CTR, PCT, and CT2 conditions behave similarly with an increase of CTs up to 101 days and a subsequent decrease in the last sampling point. CT1 shows a less accentuated growth until day 73, suggesting a delay in the synthesis of CTs in the pear skin and this treatment has a potential positive effect at preventing SC appearance in NA storage conditions. In DCA storage (Figure 6b), the coated conditions have a similar behavior between them but differ from the CTR by presenting a lower CTs content from day 32 until the end of the assay. This observation indicates a positive effect of the presence of the coating on CTs content when stored at DCA.

### 3.3.3. Volatile Metabolites Analysis

In order to make a more comprehensive analysis of the evolution of the volatile compounds profile emitted by coated pears during the assay, a subsequent HCA was performed with the complete dataset comprising the 57 compounds identified (Table 1) for both NA (Figure 7) and DCA (Figure 8). This attempts to evaluate the impact of plant-based coatings on potential aroma and/or ripening status related compounds on "Rocha" pears throughout the storage. Regarding NA (Figure 7), HCA shows that over the storage period, a clear clustering of any coating condition is not observed. Replicates of each condition are dispersed and a clear distinction between them is not observed.

In the DCA storage (Figure 8), clustering formation along storage is more evident. At nine days, no clustering is observed and each condition samples are dispersed. However, at 32 days, two main branches are formed, one corresponding to CTR samples, and the other containing the coated conditions clustered according to the type of coating. The distinction of these two branches is further increased at 134 days through a higher Euclidean distance. Additionally, from day 32 to 134, coated samples became more similar among them from day 32 to day 134, although no clustering according to the type of coating is observed. The relative content of chemical families between CTR and coated conditions has a similar behavior to the NA storage, however, is more evident in this case. Coated samples have a lower content of esters and sesquiterpenes, while monoterpenes contents appear to be increased as compared with CTR.

The content of specific volatile compounds ($\alpha$-farnesene and MHO) associated with oxidative processes, namely SC, between coating conditions at day 134, is presented in Figure 9. For NA, although no significant differences are observed in $\alpha$-farnesene content (Figure 9a) between the four coating conditions, the MHO content (Figure 9b) is significantly increased in all coated conditions as compared with the control. In DCA, the only coating that presents a lower $\alpha$-farnesene content (Figure 9c) is CT2 as compared with the control. In this kind of storage, MHO content (Figure 9d) only differs between CT1 and CT2 where the latter is inferior.

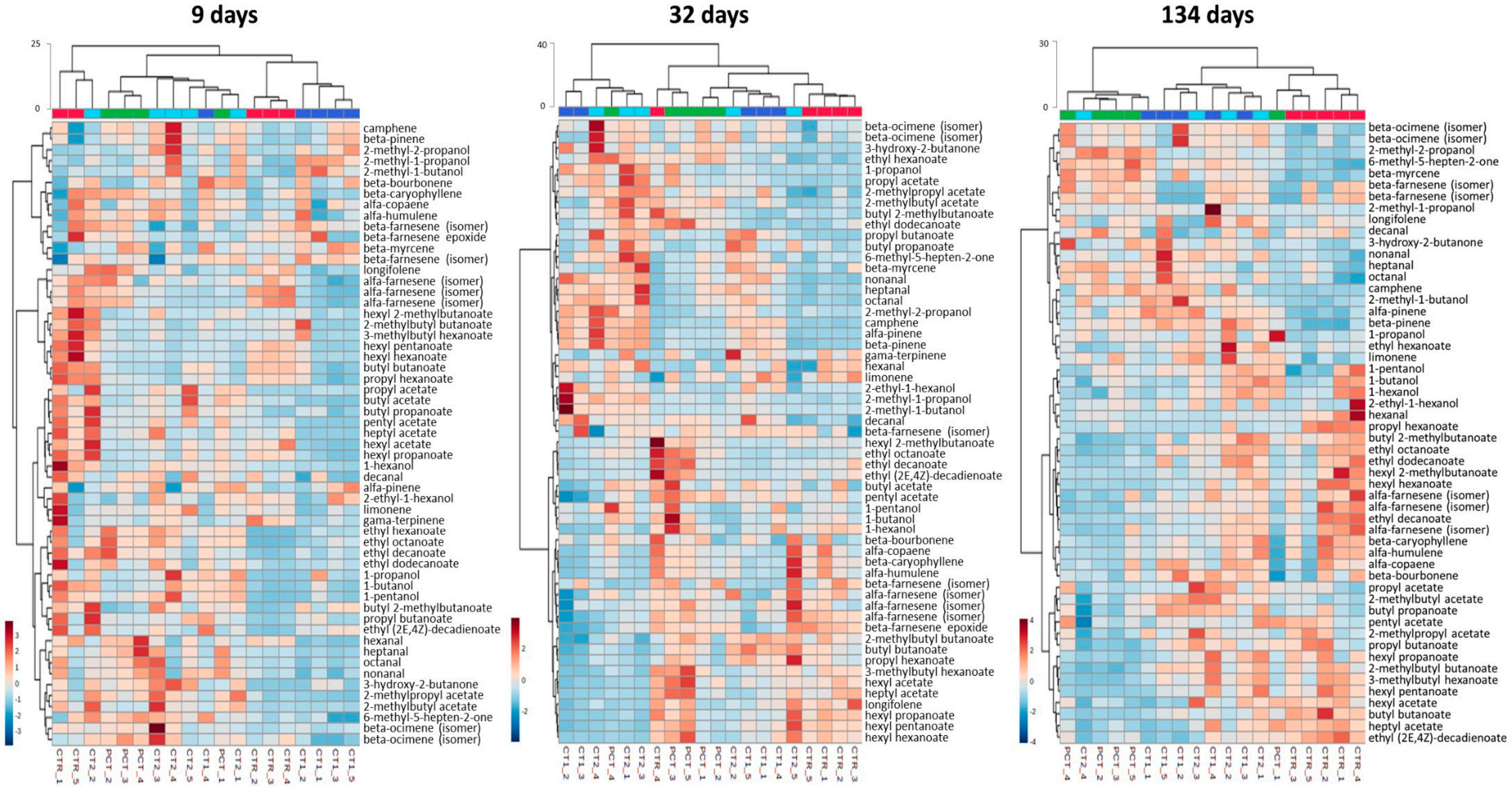


**Figure 7.** HCA heatmap visualization of the 57 selected compounds at 9, 32, and 134 days in NA (normal atmosphere) storage conditions. Euclidean distances are included on the dendrogram Y-axis. The relative content of each compound, illustrated through a chromatic scale (from low (dark blue) to high chromatographic area (dark red black)), corresponds to its peak area normalized by autoscaling. ■ CTR (control); ■ PCT (pectin); ■ CT1 (coating 1); ■ CT2 (coating 2).

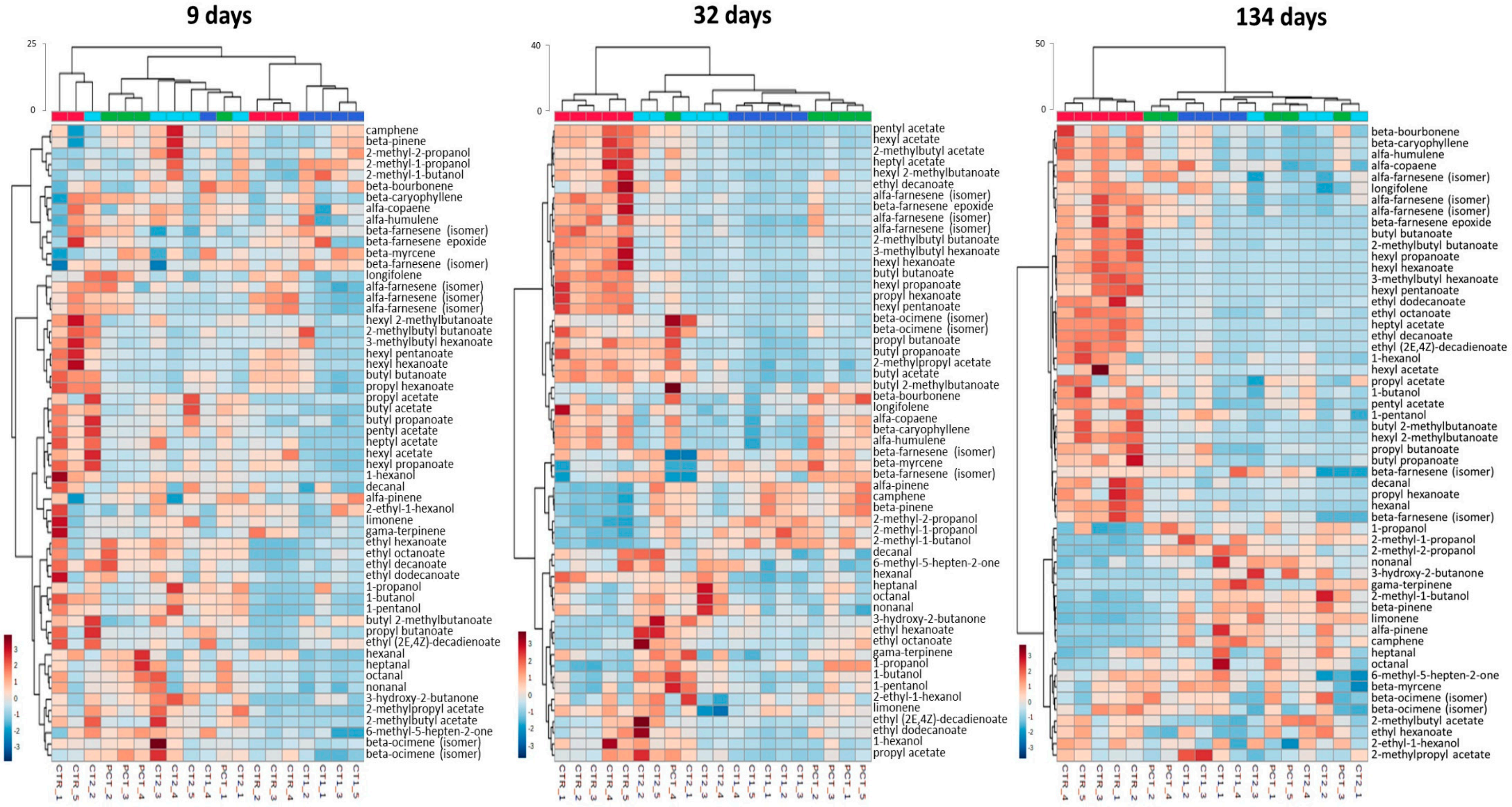


**Figure 8.** HCA heatmap visualization of the 57 selected compounds at 9, 32, and 134 days in DCA (dynamic controlled atmosphere) storage conditions. Euclidean distances are included on the dendrogram Y-axis. The relative content of each compound, illustrated through a chromatic scale (from low (dark blue) to high chromatographic area (dark red black)), corresponds to its peak area normalized by autoscaling. ■ CTR (control); ■ PCT (pectin); ■ CT1 (coating 1); ■ CT2 (coating 2).

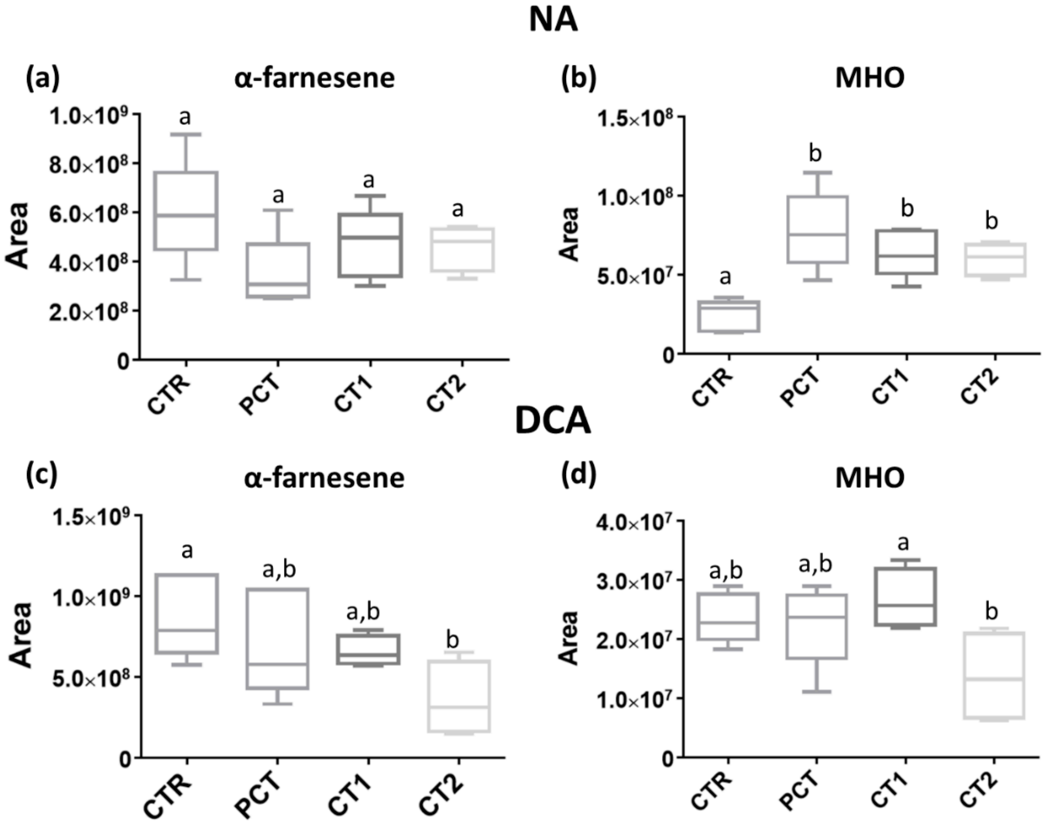


**Figure 9.** Boxplot representation of absolute areas of α-farnesene and 6-methyl-5-hepten-2-one (MHO) at day 134. (**a**,**b**) NA (normal atmosphere); (**c**,**d**) DCA (dynamic controlled atmosphere) storage conditions. For each storage, four coating conditions are presented as follows: CTR (control), PCT (pectin), CT1 (coating 1), and CT2 (coating 2). Each boxplot represents the areas of five replicates. The same lowercase letter indicates no significant differences between conditions ($p < 0.05$).

## 4. Discussion

The chromatographic approach applied in this study was able to successfully contribute to the elucidation of the "Rocha" cultivar volatile profile. Overall, a high chromatographic resolution was achieved while maintaining a good spectral quality for trace peaks. As in most fruits, volatile profiles in pears are mainly constituted of aliphatic esters and it has been shown that their concentrations in different cultivars determined their organoleptic attributes and influence consumers preferences [43]. Most volatile compounds detected in "Rocha" pear headspace were in accordance with other studies of volatiles compounds released by European pears, in which acetate and butanoate esters were the most abundant components of this family [48,49]. However, α-fanesene represented 44% of total released compounds reported here, which was a value considerably higher than the one previously reported by Avelar et al. (1.21%) on ripe "Rocha" pears. Noteably, α-fanesene is a characteristic compound of pome fruits known to accumulate in pear skin across storage [50]. Since the pears analyzed in this study had already been stored for six months, high levels of α-farnesene were expected, and therefore explained the reported results.

On the basis of the several parameters analyzed across the four-month pilot scale storage assay, it is possible to observe a distinct impact of coating application between NA and DCA conditions. Overall, the presence of coatings in NA storage had little or no impact as compared with the uncoated pears. Regarding TSS, previous studies have demonstrated a similar behavior in which coatings had no impact on this quality parameter, although an increase was observed along the initial months of storage due to the conversion of starch into soluble sugars [13,51]. In the present study, once coatings were applied to pears after six months of storage, they had most likely already reached their maximum

TSS content, hence, a constant TSS content was observed across the assay. Hue angle was also slightly affected by coatings in NA storage. Maintenance of hue color between control and all coated pears right after coating application is indeed considered to be an advantage of these types of coatings, since it demonstrates their optical clarity, and thus does not have a negative visual impact on treated fruits. However, the slight difference of hue angle in control and coated pears indicates that coatings were not capable of slowing down the ripening process. This absence of a clear preservation increase under NA storage was also visible in the volatile compounds emitted by pears. The HCA analysis of the whole dataset of identified compounds did not exhibit a clear clustering in coating conditions or the control, and there were no clear differences in relative amounts of chemical families among them. CTs evolution also corroborates other parameters' behavior, since coatings were not able to prevent their accumulation except for CT1, where a slight delay was observed possibly due to a superior antioxidant potential of this extract. Overall, in this kind of storage, although desirable characteristics were maintained using coatings, an increased preservation and prevention of SC was not observed, possibly due to a low antioxidant activity of the extracts used.

Coated pears stored under DCA suffered different effects as compared with NA. Although the TSS content did not suffer an impact due to the coating presence or the type of storage, in DCA storage, tested conditions were able to delay yellowing of the fruit. Since this was also verified in the control condition, this effect was attributed to the type of storage, which has been previously reported to delay the yellowing of pear skin as compared with NA [52]. In DCA, released volatiles and CTs accumulation were found to be affected due to the presence of coatings. However, as in NA storage, no clear effect of the type of coating was observed, which shows that the extracts used do not exert a significant antioxidant effect on pears. Differences observed between coated and uncoated fruits could be due to the additional barrier that coatings represent to fruit respiration [53]. Since DCA storage imposes a low $O_2$ atmosphere, coating presence could be further limiting $O_2$ availability to the fruit. This limitation on fruit respiration could be responsible for volatile differences on coated fruits as compared with the control. Ultra-low oxygen storage (0.8 kPa $O_2$) applied to "Bartlett' pears has been shown to suppress synthesis of esters as well as most aroma volatiles [21], which was in agreement with this study. Although several pear cultivars can be susceptible to lower $pO_2$, "Rocha" pear has shown a very high storage capacity at around 0.5 kPa during 4.5 months of storage [54]. Furthermore, "Rocha" pear was also successfully stored for 8.5 months at static 0.5 kPa $O_2$ without occurrence of storage disorders and maintained adequate fruit quality after 7 d of shelf-life [55].

Although levels of α-farnesene and MHO do not show a significant difference between the control and coated conditions at the end of the storage, the CTs content were found to be inferior for coated conditions from day 32 until the end of the assay. At day 134, the CTs content observed was reduced by 31.8%, 38.8%, and 44.7% for PCT, CT1, and CT2, respectively, which indicated a possible protective effect of the coatings against SC. This was in agreement with a previous study that demonstrated that low temperature and low oxygen levels were able to reduce the degradation of α-farnesene [56].

## 5. Conclusions

HS-SPME/GC×GC-ToFMS allowed us to detect hundreds of instrumental features, among which, a set of 57 compounds potentially related with aroma and/or ripening status of "Rocha" pears. Monitorization of such compounds over a four-month storage assay showed that coating application had an impact on their release under DCA storage but not in NA conditions. In the former, coatings decreased the release of "Rocha" pear volatiles potentially related with aroma or ripening status, namely, esters and sesquiterpenes, and showed a lower CTs content as compared with the control. The TSS content and color were not affected by coatings. These results indicate that the combination of pectin coatings with DCA storage can delay fruit ripening and prevent SC development, thus, contributing to the preservation of "Rocha" pears for longer periods. Impacts observed are mainly attributed to the coating as a gas barrier in the surface of the fruit, since coatings enriched with plant extracts had a similar behavior to PCT coating on measured parameters.

**Supplementary Materials:** The following are available online at http://www.mdpi.com/2304-8158/9/9/1299/s1, Table S1: Full dataset used for statistics processing of the identified "Rocha" pear volatile compounds using HS-SPME/GC×GC-ToFMS, including relevant chromatographic data used to assess compounds identification and respective odor descriptors, Figure S1: Dendrograms of the hierarchical clustering analysis (HCA) for previously reported volatile compounds of "Rocha" pears at 9, 32, and 134 days of the assay in NA (normal atmosphere) storage conditions. Euclidean distances are included on the dendrogram Y-axis. The relative content of each compound, illustrated through a chromatic scale (from low (dark blue) to high chromatographic area (dark red black)), corresponds to its peak area normalized by autoscaling. ■ CTR (control); ■ PCT (pectin); ■ CT1 (coating 1); ■ CT2 (coating 2). Figure S2: Dendrograms of the hierarchical clustering analysis (HCA) for previously reported volatile compounds of 'Rocha' pear at 9, 32 and 134 days of the assay in DCA (dynamic controlled atmosphere) storage conditions. Euclidean distances are included on the dendrogram Y-axis. The relative content of each compound, illustrated through a chromatic scale (from low (dark blue) to high chromatographic area (dark red black)), corresponds to its peak area normalized by autoscaling. ■ CTR (control); ■ PCT (pectin); ■ CT1 (coating 1); ■ CT2 (coating 2).

**Author Contributions:** Conceptualization A.L.A., A.J.D.S., M.P., N.I., and S.M.R.; investigation, methodology, validation A.M.A.F., C.D., and A.L.A.; formal analysis A.M.A.F. and C.D.; writing—original draft preparation A.M.A.F. and C.D.; writing—review and editing, A.L.A., A.J.D.S., A.M.A.F., C.D., M.P., N.I., and S.M.R.; supervision A.J.D.S. and S.M.R. All authors have read and agreed to the published version of the manuscript.

**Funding:** This work received external funding of the project "ReStoragePear" grant number 17777, supported by the COMPETE Operational Programme (COMPETE 2020) under the PORTUGAL 2020 Partnership Agreement through the European Regional Development Fund (ERDF).

**Acknowledgments:** The authors thank the scientific collaboration of CBQF under the FCT project UID/Multi/50016/2013, and the financial support of QOPNA (UID/QUI/00062/2019), LAQV-REQUIMTE (UIDB/50006/2020), and CICECO (UIDB/50011/2020 & UIDP/50011/2020), through national funds and FEDER, within the PT2020 Partnership Agreement. We also thank Cooperativa Agrícola dos Fruticultores do Cadaval (COOPVAL) for providing the fruit and the storage facilities, as well as Medronhalva. LDA. and INDUMAPE - Industrialização de Fruta, S.A. for providing *A. unedo* materials and apple pomace.

**Conflicts of Interest:** The authors declare that they have no conflict of interest.

## References

1. Instituto Nacional de Estatística. *Estatísticas Agrícolas: 2018*; INE: Lisboa, Portugal, 2019; ISBN 978-989-25-0495-7.
2. Pêra Rocha—A Pera Rocha Distingue-se Pelas suas Características Únicas, que Fazem dela um Sabor de Portugal. Available online: http://perarocha.pt/ (accessed on 5 June 2020).
3. Saquet, A.A. Storage of pears. *Sci. Hortic.* **2019**, *246*, 1009–1016. [CrossRef]
4. Saquet, A.A.; Almeida, D.P.F. Ripening physiology and biochemistry of 'Rocha' pear as affected by ethylene inhibition. *Postharvest Biol. Technol.* **2017**, *125*, 161–167. [CrossRef]
5. Isidoro, N.; Almeida, D.P.F. α-Farnesene, conjugated trienols, and superficial scald in "Rocha" pear as affected by 1-methylcyclopropene and diphenylamine. *Postharvest Biol. Technol.* **2006**, *42*, 49–56. [CrossRef]
6. Rapparini, F.; Predieri, S. Pear fruit volatiles. In *Horticultural Reviews*; Janick, J., Ed.; John Wiley & Sons: Hoboken, NJ, USA, 2003; Volume 28, pp. 237–324. ISBN 9780471215424.
7. Zhou, S.; Cheng, Y.; Guan, J. The molecular basis of superficial scald development related to ethylene perception and α-farnesene metabolism in 'Wujiuxiang' pear. *Sci. Hortic.* **2017**, *216*, 76–82. [CrossRef]
8. Lurie, S.; Watkins, C.B. Superficial scald, its etiology and control. *Postharvest Biol. Technol.* **2012**, *65*, 44–60. [CrossRef]
9. Dias, C.; Amaro, A.L.; Salvador, Â.C.; Silvestre, A.J.D.; Rocha, S.M.; Isidoro, N.; Pintado, M. Strategies to Preserve Postharvest Quality of Horticultural Crops and Superficial Scald Control: From Diphenylamine Antioxidant Usage to More Recent Approaches. *Antioxidants* **2020**, *9*, 356. [CrossRef]
10. Lin, D.; Zhao, Y. Innovations in the development and application of edible coatings for fresh and minimally processed fruits and vegetables. *Compr. Rev. Food Sci. Food Saf.* **2007**, *6*, 60–75. [CrossRef]
11. Baraiya, N.S.; Rao, T.V.R.; Thakkar, V.R. Improvement of Postharvest quality and storability of jamun fruit (*Syzygium cumini* L. var. Paras) by zein coating enriched with antioxidants. *Food Bioprocess Technol.* **2015**, *8*, 2225–2234. [CrossRef]

12. Poverenov, E.; Rutenberg, R.; Danino, S.; Horev, B.; Rodov, V. Gelatin-chitosan composite films and edible coatings to enhance the quality of food products: Layer-by-layer vs. Blended formulations. *Food Bioprocess Technol.* **2014**, *7*, 3319–3327. [CrossRef]
13. Gago, C.; Antão, R.; Dores, C.; Guerreiro, A.; Miguel, M.G.; Faleiro, M.L.; Figueiredo, A.C.; Antunes, M.D. The effect of nanocoatings enriched with essential oils on "Rocha" pear long storage. *Foods* **2020**, *9*, 240. [CrossRef]
14. Deng, Z.; Jung, J.; Simonsen, J.; Wang, Y.; Zhao, Y. Cellulose nanocrystal reinforced chitosan coatings for improving the storability of postharvest pears under both ambient and cold storages. *J. Food Sci.* **2017**, *82*, 453–462. [CrossRef] [PubMed]
15. Kähkönen, M.P.; Hopia, A.I.; Vuorela, H.J.; Rauha, J.P.; Pihlaja, K.; Kujala, T.S.; Heinonen, M. Antioxidant activity of plant extracts containing phenolic compounds. *J. Agric. Food Chem.* **1999**, *47*, 3954–3962. [CrossRef] [PubMed]
16. Sarkar, D.; Ankolekar, C.; Greene, D.; Shetty, K. Natural preservatives for superficial scald reduction and enhancement of protective phenolic-linked antioxidant responses in apple during post-harvest storage. *J. Food Sci. Technol.* **2018**, *55*, 1767–1780. [CrossRef]
17. Sharma, S.; Rao, T.V.R. Xanthan gum based edible coating enriched with cinnamic acid prevents browning and extends the shelf-life of fresh-cut pears. *LWT Food Sci. Technol.* **2015**, *62*, 791–800. [CrossRef]
18. Dias, C.; Fonseca, A.M.A.; Amaro, A.L.; Vilas-Boas, A.A.; Oliveira, A.; Santos, S.A.O.; Silvestre, A.J.D.; Rocha, S.M.; Isidoro, N.; Pintado, M. Natural-based antioxidant extracts as potential mitigators of fruit browning. *Antioxidants* **2020**, *9*, 715. [CrossRef] [PubMed]
19. Chen, J.L.; Yan, S.; Feng, Z.; Xiao, L.; Hu, X.S. Changes in the volatile compounds and chemical and physical properties of Yali pear (*Pyrus bertschneideri* Reld) during storage. *Food Chem.* **2006**, *97*, 248–255. [CrossRef]
20. Ji, L.C.; Ji, H.W.; Wang, Q.; Deng, H.; Xiao, S.H. Changes in the volatile compounds and chemical and physical properties of Kuerle fragrant pear (*Pyrus serotina* Reld) during storage. *J. Agric. Food Chem.* **2006**, *54*, 8842–8847. [CrossRef]
21. Zlatić, E.; Zadnik, V.; Fellman, J.; Demšar, L.; Hribar, J.; Čejić, Ž.; Vidrih, R. Comparative analysis of aroma compounds in "Bartlett" pear in relation to harvest date, storage conditions, and shelf-life. *Postharvest Biol. Technol.* **2016**, *117*, 71–80. [CrossRef]
22. Hendges, M.V.; Neuwald, D.A.; Steffens, C.A.; Vidrih, R.; Zlatić, E.; do Amarante, C.V.T. 1-MCP and storage conditions on the ripening and production of aromatic compounds in Conference and Alexander Lucas pears harvested at different maturity stages. *Postharvest Biol. Technol.* **2018**, *146*, 18–25. [CrossRef]
23. Rizzolo, A.; Polesello, A. Use of headspace capillary GC to study the development of volatile compounds in fresh fruit. *J. High Resolut. Chromatogr.* **1992**, *15*, 472–477. [CrossRef]
24. Jayanty, S.; Song, J.; Rubinstein, N.M.; Chong, A.; Beaudry, R.M. Temporal relationship between ester biosynthesis and ripening events in bananas. *J. Am. Soc. Hortic. Sci.* **2002**, *127*, 998–1005. [CrossRef]
25. Avelar, M.L.; Rizzolo, A.; Lombardi, P.; Eccher Zerbini, P. Physiological and quality responses of "Rocha" pear to controlled atmospher storage. In Proceedings of the Sixth International Symposium of the European Concerted Action Program COST 94, Oosterbeek, The Netherlands, 19–22 October 1994; Woltering, E.J., Gorris, L.G., Jongen, W.M.F., McKcnna, B., Höhn, E., Bertolini, P., Woolfe, M.L., de Jager, A., Ahvenainen, R., Calero, F.A., Eds.; European Communities: Oosterbeek, The Netherlands, 1994; pp. 15–22.
26. Gomes, M.H.; Beaudry, R.M.; Almeida, D.P.F. Volatile profile of fresh-cut 'Rocha' pear under various temperatures and oxygen levels. *Acta Hortic.* **2018**, 247–250. [CrossRef]
27. Pott, D.M.; Vallarino, J.G.; Osorio, S. Metabolite changes during postharvest storage: Effects on fruit quality traits. *Metabolites* **2020**, *10*, 187. [CrossRef] [PubMed]
28. Wang, C.; Zhang, W.; Li, H.; Mao, J.; Guo, C.; Ding, R.; Wang, Y.; Fang, L.; Chen, Z.; Yang, G. Analysis of volatile compounds in pears by HS-SPME-GC×GC-TOFMS. *Molecules* **2019**, *24*, 1795. [CrossRef]
29. Górecki, T.; Harynuk, J.; Panić, O. The evolution of comprehensive two-dimensional gas chromatography (GC×GC). *J. Sep. Sci.* **2004**, *27*, 359–379. [CrossRef]
30. Martins, C. Yeasts and Beers Volatile Metabolomic Profiling: Advances In Lager Beer Aroma Comprehension. Ph.D. Thesis, Universidade de Aveiro, Aveiro, Portugal, 2018.
31. Oms-Oliu, G.; Soliva-Fortuny, R.; Martín-Belloso, O. Edible coatings with antibrowning agents to maintain sensory quality and antioxidant properties of fresh-cut pears. *Postharvest Biol. Technol.* **2008**, *50*, 87–94. [CrossRef]

32. Anet, E.F.L.J. Superficial scald, a functional disorder of stored apples. IX. Effect of maturity and ventilation. *J. Sci. Food Agric.* **1972**, *23*, 763–769. [CrossRef]
33. Sumner, L.W.; Amberg, A.; Barrett, D.; Beale, M.H.; Beger, R.; Daykin, C.A.; Fan, T.W.-M.; Fiehn, O.; Goodacre, R.; Griffin, J.L.; et al. Proposed minimum reporting standards for chemical analysis Chemical Analysis Working Group (CAWG) Metabolomics Standards Initiative (MSI). *Metabolomics* **2007**, *3*, 211–221. [CrossRef]
34. Salvador, Â.C.; Rudnitskaya, A.; Silvestre, A.J.D.; Rocha, S.M. Metabolomic-based strategy for fingerprinting of *Sambucus nigra* L. berry volatile terpenoids and norisoprenoids: Influence of ripening and cultivar. *J. Agric. Food Chem.* **2016**, *64*, 5428–5438. [CrossRef]
35. Martins, C.; Brandão, T.; Almeida, A.; Rocha, S.M. Unveiling the lager beer volatile terpenic compounds. *Food Res. Int.* **2018**, *114*, 199–207. [CrossRef]
36. Dallüge, J.; Beens, J.; Brinkman, U.A.T. Comprehensive two-dimensional gas chromatography: A powerful and versatile analytical tool. *J. Chromatogr. A* **2003**, *1000*, 69–108. [CrossRef]
37. van Den Dool, H.; Kratz, P.D. A generalization of the retention index system including linear temperature programmed gas—Liquid partition chromatography. *J. Chromatogr. A* **1963**, *11*, 463–471. [CrossRef]
38. Song, J.; Bangerth, F. Fatty acids as precursors for aroma volatile biosynthesis in pre-climacteric and climacteric apple fruit. *Postharvest Biol. Technol.* **2003**, *30*, 113–121. [CrossRef]
39. Suwanagul, A. Ripening Pear Flavor Volatiles: Identification, Biosynthesis And Sensory Perception. Ph.D. Thesis, Horticulture, Oregon State University, Corvallis, OR, USA, 1996.
40. Burdock, G. *Fenaroli's Handbook of Flavor Ingredients*, 5th ed.; CRC Press: Boca Raton, FL, USA, 2005.
41. Yan, D.; Shi, J.; Ren, X.; Tao, Y.; Ma, F.; Li, R.; Liu, X.; Liu, C. Insights into the aroma profiles and characteristic aroma of 'Honeycrisp' apple (Malus × domestica). *Food Chem.* **2020**, *327*, 127074. [CrossRef]
42. Tian, H.; Zhan, P.; Deng, Z.; Yan, H.; Zhu, X. Development of a flavour fingerprint by GC-MS and GC-O combined with chemometric methods for the quality control of Korla pear ( *Pyrus serotina* Reld). *Int. J. Food Sci. Technol.* **2014**, *49*, 2546–2552. [CrossRef]
43. Li, G.; Jia, H.; Wu, R.; Hussain, S.; Teng, Y. Characterization of aromatic volatile constituents in 11 Asian pear cultivars belonging to different species. *Afr. J. Agric. Res.* **2012**, *7*, 4761–4770. [CrossRef]
44. Hui, Y.H.; Chen, F.; Nollet, L.M.L.; Guiné, R.P.F.; Le Quéré, J.L.; Martín-Belloso, O.; Mínguez-Mosquera, M.I.; Paliyath, G.; Pessoa, F.L.P.; Sidhu, J.S.; et al. *Handbook of Fruit and Vegetable Flavors*; Hui, Y.H., Ed.; John Wiley & Sons, Inc.: Hoboken, NJ, USA, 2010; ISBN 9780470622834.
45. Yi, X.K.; Liu, G.F.; Rana, M.M.; Zhu, L.W.; Jiang, S.L.; Huang, Y.F.; Lu, W.M.; Wei, S. Volatile profiling of two pear genotypes with different potential for white pear aroma improvement. *Sci. Hortic.* **2016**, *209*, 221–228. [CrossRef]
46. Almeida, D.P.; Carvalho, R.; Dupille, E. Efficacy of 1-methylcyclopropene on the mitigation of storage disorders of "Rocha" pear under normal refrigerated and controlled atmospheres. *Food Sci. Technol. Int.* **2016**, *22*, 399–409. [CrossRef]
47. Giné Bordonaba, J.; Matthieu-Hurtiger, V.; Westercamp, P.; Coureau, C.; Dupille, E.; Larrigaudière, C. Dynamic changes in conjugated trienols during storage may be employed to predict superficial scald in "Granny Smith" apples. *LWT Food Sci. Technol.* **2013**, *54*, 535–541. [CrossRef]
48. Shiota, H. Changes in the volatile composition of La France pear during maturation. *J. Sci. Food Agric.* **1990**, *52*, 421–429. [CrossRef]
49. Rapparini, F.; Predieri, S. Volatile constituents of "Harrow" sweet pears by dynamic headspace technique. *Acta Hortic.* **2002**, 811–816. [CrossRef]
50. Paillard, N.M.M. The flavour of apples, pears and quinces. *Dev. Food Sci.* **1990**, *3*, 1–41.
51. Medeiros, B.G.d.S.; Pinheiro, A.C.; Teixeira, J.A.; Vicente, A.A.; Carneiro-da-Cunha, M.G. Polysaccharide/Protein nanomultilayer coatings: Construction, characterization and evaluation of their effect on "Rocha" pear (*Pyrus communis* L.) shelf-life. *Food Bioprocess Technol.* **2012**, *5*, 2435–2445. [CrossRef]
52. Saquet, A.A. Storability of 'conference' pear under various controlled atmospheres. *Erwerbs Obstbau* **2018**, *60*, 275–280. [CrossRef]
53. Lima, Á.M.; Cerqueira, M.A.; Souza, B.W.S.; Santos, E.C.M.; Teixeira, J.A.; Moreira, R.A.; Vicente, A.A. New edible coatings composed of galactomannans and collagen blends to improve the postharvest quality of fruits—Influence on fruits gas transfer rate. *J. Food Eng.* **2010**, *97*, 101–109. [CrossRef]

54. Saquet, A.; Almeida, D. Internal disorders of 'Rocha' pear affected by oxygen partial pressure and inhibition of ethylene action. *Postharvest Biol. Technol.* **2017**, *128*, 54–62. [CrossRef]
55. Saquet, A.A.; Streif, J.; Almeida, D.P.F. Responses of 'Rocha' pear to delayed controlled atmosphere storage depend on oxygen partial pressure. *Sci. Hortic.* **2017**, *222*, 17–21. [CrossRef]
56. Chen, P.M.; Varga, R.J.; Xiao, Y.Q. Inhibition of α-farnesene biosynthesis and its oxidation in the peel tissue of "d'Anjou" pears by low-$O_2$/elevated $CO_2$ atmospheres. *Postharvest Biol. Technol.* **1993**, *3*, 215–223. [CrossRef]

© 2020 by the authors. Licensee MDPI, Basel, Switzerland. This article is an open access article distributed under the terms and conditions of the Creative Commons Attribution (CC BY) license (http://creativecommons.org/licenses/by/4.0/).
