# Supplementary material for: The Impact of Plant-Based Coatings in “ROCHA” Pear Preservation during Cold Storage: A Metabolomic Approach
Source: Foods. 2020 Sep 15;9(9):1299. doi: 10.3390/foods9091299 (PMC7555455; doi:10.3390/foods9091299)
Supplement: Supplementary file 1 [file foods-09-01299-s001.zip › foods-904747-tex.zip!/Definitions/foods-904747-g001.pdf]

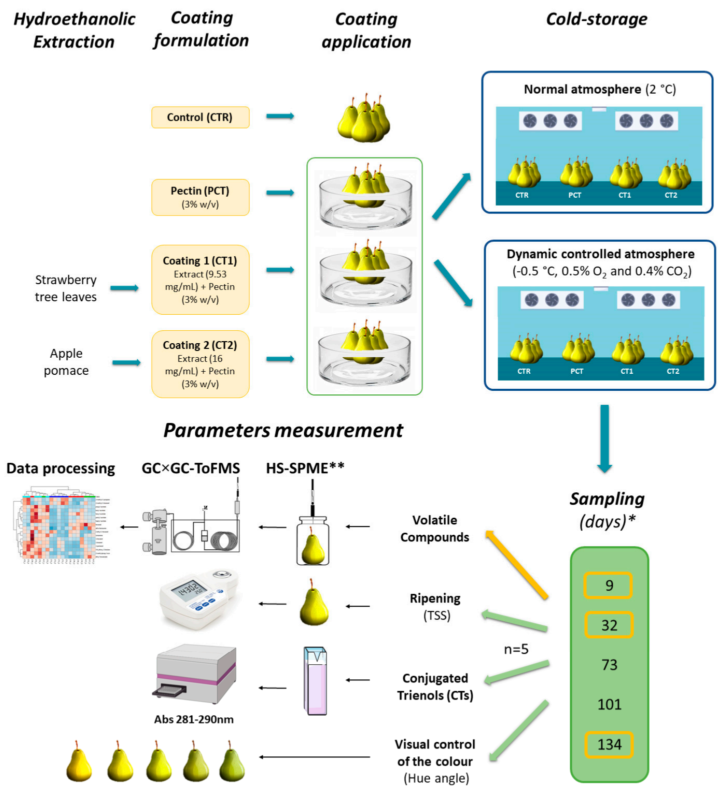

Hydroethanolic Extraction
Coating formulation
Coating application
Cold-storage
Control (CTR)
Pectin (PCT)
(3% w/v)
Strawberry tree leaves
Coating 1 (CT1)
Extract (9.53 mg/mL) + Pectin (3% w/v)
Apple pomace
Coating 2 (CT2)
Extract (16 mg/mL) + Pectin (3% w/v)
Normal atmosphere (2 °C)
CTR
PCT
CT1
CT2
Dynamic controlled atmosphere
(-0.5 °C, 0.5% O_2 and 0.4% CO_2)
CTR
PCT
CT1
CT2
Parameters measurement
Data processing
GC×GC-ToFMS
HS-SPME**
Sampling (days)*
Volatile Compounds
Ripening (TSS)
n=5
Conjugated Trienols (CTs)
Abs 281-290nm
Visual control of the colour (Hue angle)
9
32
73
101
134
