# Supplementary material for: The Impact of Plant-Based Coatings in “ROCHA” Pear Preservation during Cold Storage: A Metabolomic Approach
Source: Foods. 2020 Sep 15;9(9):1299. doi: 10.3390/foods9091299 (PMC7555455; doi:10.3390/foods9091299)
Supplement: Supplementary file 1 [file foods-09-01299-s001.zip › foods-904747-tex.zip!/Definitions/foods-904747-g002.pdf]

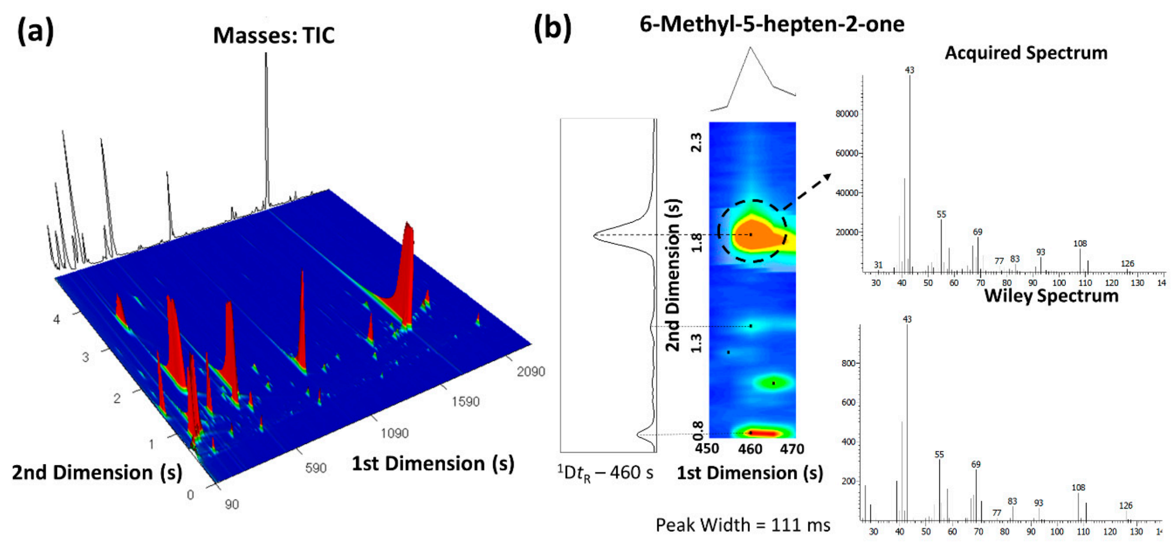

(a)
Masses: TIC
2nd Dimension (s)
1st Dimension (s)
0
1
2
3
4
90
590
1090
1590
2090
(b)
6-Methyl-5-hepten-2-one
2nd Dimension (s)
2.3
1.8
1.3
0.8
450
460
470
1st Dimension (s)
$^{1}Dt_R$ – 460 s
Peak Width = 111 ms
Acquired Spectrum
Wiley Spectrum
43
55
69
77
83
93
108
126
31
