# Supplementary material for: The Impact of Plant-Based Coatings in “ROCHA” Pear Preservation during Cold Storage: A Metabolomic Approach
Source: Foods. 2020 Sep 15;9(9):1299. doi: 10.3390/foods9091299 (PMC7555455; doi:10.3390/foods9091299)
Supplement: Supplementary file 1 [file foods-09-01299-s001.zip › foods-904747-tex.zip!/Definitions/foods-904747-g003.pdf]

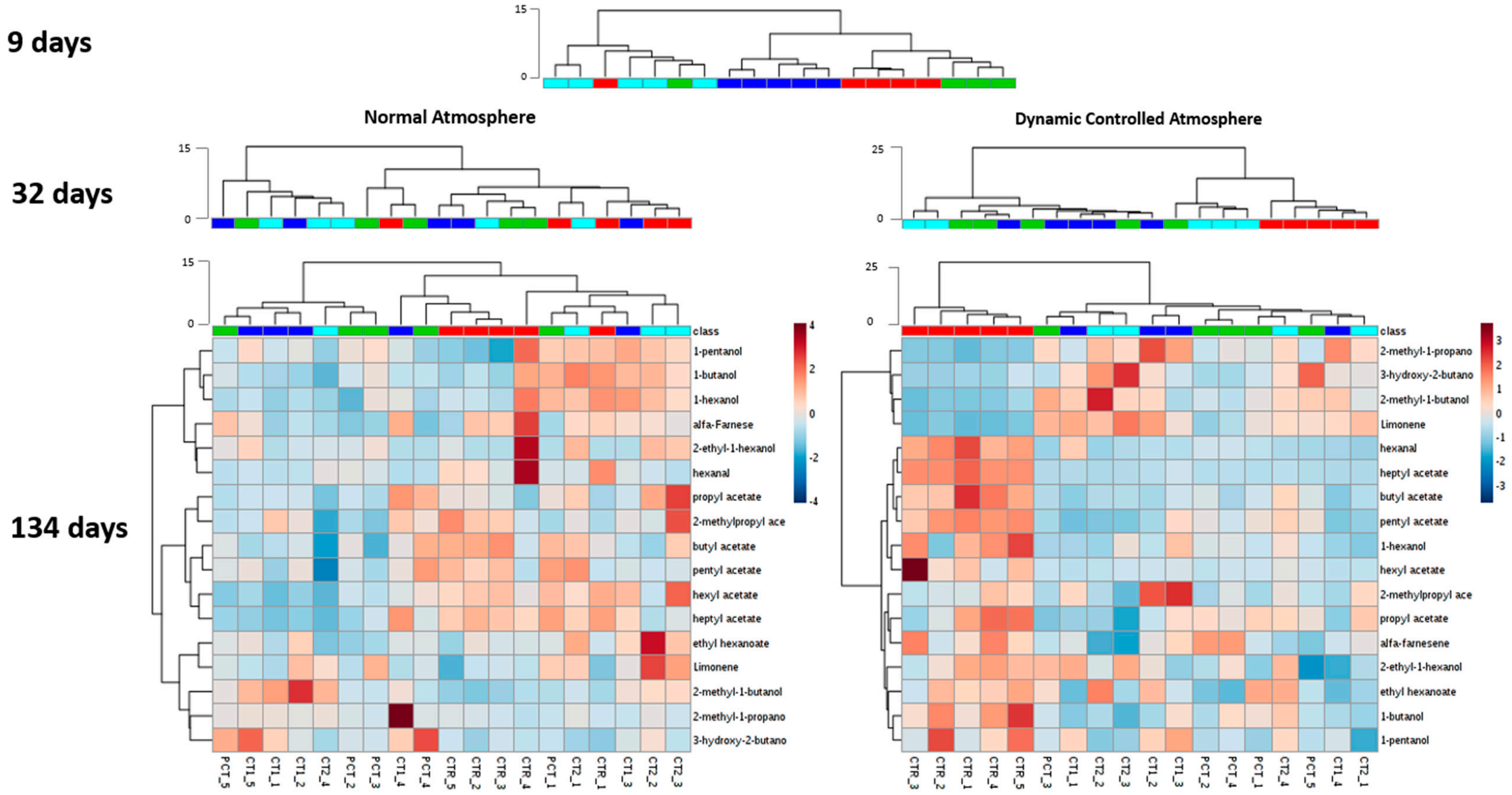
9 days
32 days
134 days
Normal Atmosphere
Dynamic Controlled Atmosphere
15
0
25
class
1-pentanol
1-butanol
1-hexanol
alfa-Farnese
2-ethyl-1-hexanol
hexanal
propyl acetate
2-methylpropyl ace
butyl acetate
pentyl acetate
hexyl acetate
heptyl acetate
ethyl hexanoate
Limonene
2-methyl-1-butanol
2-methyl-1-propano
3-hydroxy-2-butano
PCT_5
CT1_5
CT1_1
CT1_2
CT2_4
PCT_2
PCT_3
CT1_4
PCT_4
CTR_5
CTR_2
CTR_3
CTR_4
PCT_1
CT2_1
CTR_1
CT1_3
CT2_2
CT2_3
4
2
0
-2
-4
2-methyl-1-propano
3-hydroxy-2-butano
2-methyl-1-butanol
Limonene
hexanal
heptyl acetate
butyl acetate
pentyl acetate
1-hexanol
hexyl acetate
2-methylpropyl ace
propyl acetate
alfa-farnesene
2-ethyl-1-hexanol
ethyl hexanoate
1-butanol
1-pentanol
CTR_3
CTR_2
CTR_1
CTR_4
CTR_5
PCT_3
CT1_1
CT2_2
CT2_3
CT1_2
CT1_3
PCT_2
PCT_4
PCT_1
CT2_4
PCT_5
CT1_4
CT2_1
3
2
1
0
-1
-2
-3
