# Supplementary material for: The Impact of Plant-Based Coatings in “ROCHA” Pear Preservation during Cold Storage: A Metabolomic Approach
Source: Foods. 2020 Sep 15;9(9):1299. doi: 10.3390/foods9091299 (PMC7555455; doi:10.3390/foods9091299)
Supplement: Supplementary file 1 [file foods-09-01299-s001.zip › foods-904747-tex.zip!/Definitions/foods-904747-g004.pdf]

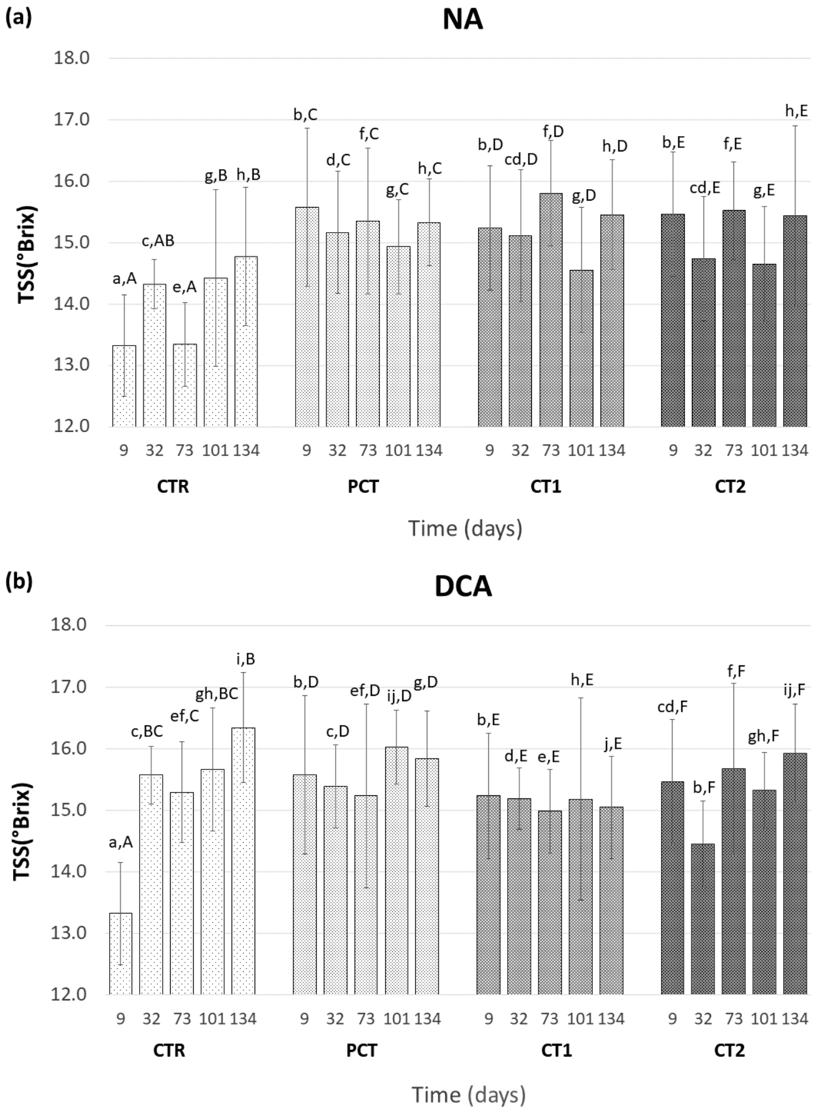
(a)
NA
18.0
17.0
16.0
15.0
14.0
13.0
12.0
TSS(°Brix)
a,A
c,AB
e,A
g,B
h,B
b,C
d,C
f,C
g,C
h,C
b,D
cd,D
f,D
g,D
h,D
b,E
cd,E
f,E
g,E
h,E
9 32 73 101 134
9 32 73 101 134
9 32 73 101 134
9 32 73 101 134
CTR
PCT
CT1
CT2
Time (days)
(b)
DCA
18.0
17.0
16.0
15.0
14.0
13.0
12.0
TSS(°Brix)
a,A
c,BC
ef,C
gh,BC
i,B
b,D
c,D
ef,D
ij,D
g,D
b,E
d,E
e,E
h,E
j,E
cd,F
b,F
f,F
gh,F
ij,F
9 32 73 101 134
9 32 73 101 134
9 32 73 101 134
9 32 73 101 134
CTR
PCT
CT1
CT2
Time (days)
