# Supplementary material for: The Impact of Plant-Based Coatings in “ROCHA” Pear Preservation during Cold Storage: A Metabolomic Approach
Source: Foods. 2020 Sep 15;9(9):1299. doi: 10.3390/foods9091299 (PMC7555455; doi:10.3390/foods9091299)
Supplement: Supplementary file 1 [file foods-09-01299-s001.zip › foods-904747-tex.zip!/Definitions/foods-904747-g005.pdf]

(a)

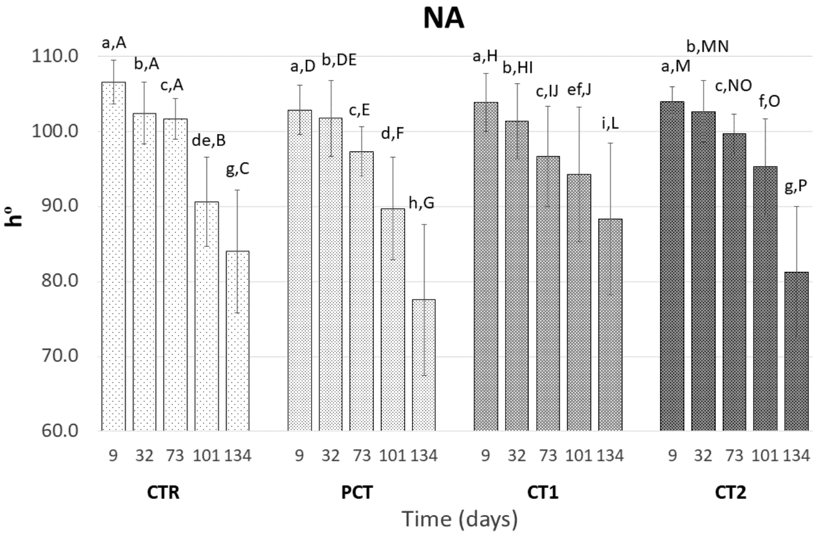

NA
110.0
100.0
90.0
80.0
70.0
60.0
h°
a,A
b,A
c,A
de,B
g,C
a,D
b,DE
c,E
d,F
h,G
a,H
b,HI
c,IJ
ef,J
i,L
a,M
b,MN
c,NO
f,O
g,P
9 32 73 101 134
CTR
PCT
CT1
CT2
Time (days)


(b)

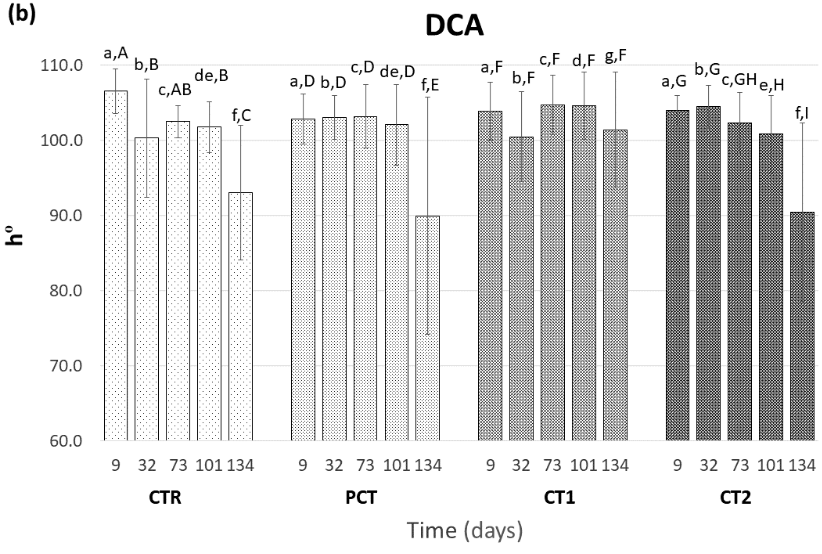

DCA
110.0
100.0
90.0
80.0
70.0
60.0
h°
a,A
b,B
c,AB
de,B
f,C
a,D
b,D
c,D
de,D
f,E
a,F
b,F
c,F
d,F
g,F
a,G
b,G
c,GH
e,H
f,I
9 32 73 101 134
CTR
PCT
CT1
CT2
Time (days)
