# Supplementary material for: The Impact of Plant-Based Coatings in “ROCHA” Pear Preservation during Cold Storage: A Metabolomic Approach
Source: Foods. 2020 Sep 15;9(9):1299. doi: 10.3390/foods9091299 (PMC7555455; doi:10.3390/foods9091299)
Supplement: Supplementary file 1 [file foods-09-01299-s001.zip › foods-904747-tex.zip!/Definitions/foods-904747-g006.pdf]

(a)

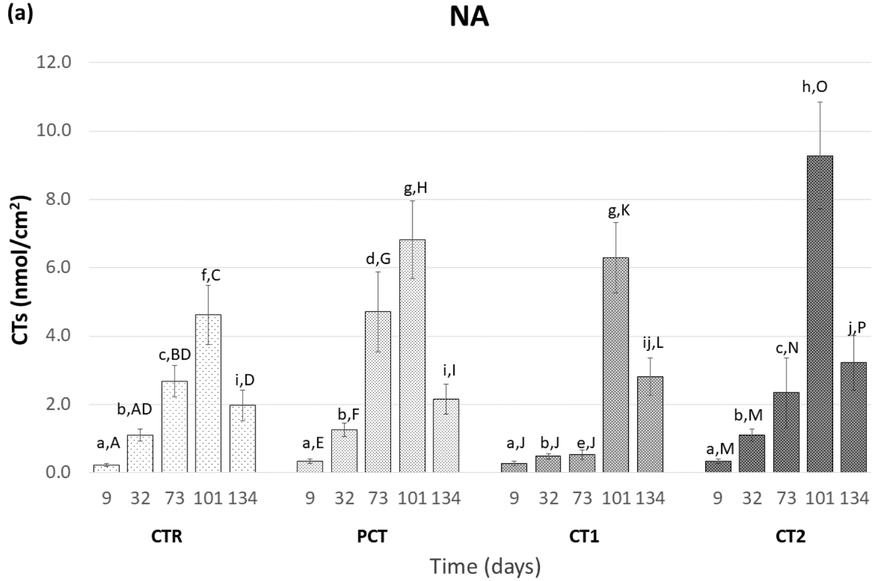
NA
CTs (nmol/cm²)
12.0
10.0
8.0
6.0
4.0
2.0
0.0
a,A
b,AD
c,BD
f,C
i,D
a,E
b,F
d,G
g,H
i,I
a,J
b,J
e,J
g,K
ij,L
a,M
b,M
c,N
h,O
j,P
9 32 73 101 134
CTR
PCT
CT1
CT2
Time (days)

(b)

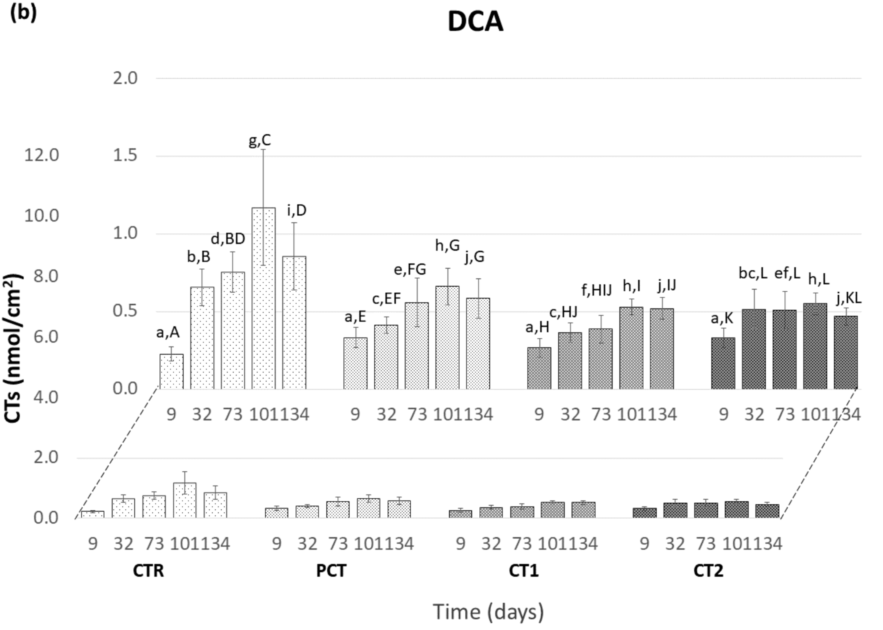
DCA
CTs (nmol/cm²)
2.0
1.5
1.0
0.5
0.0
12.0
10.0
8.0
6.0
4.0
2.0
0.0
a,A
b,B
d,BD
g,C
i,D
a,E
c,EF
e,FG
h,G
j,G
a,H
c,HJ
f,HIJ
h,I
j,IJ
a,K
bc,L
ef,L
h,L
j,KL
9 32 73 101134
CTR
PCT
CT1
CT2
Time (days)
