# Supplementary material for: The Impact of Plant-Based Coatings in “ROCHA” Pear Preservation during Cold Storage: A Metabolomic Approach
Source: Foods. 2020 Sep 15;9(9):1299. doi: 10.3390/foods9091299 (PMC7555455; doi:10.3390/foods9091299)
Supplement: Supplementary file 1 [file foods-09-01299-s001.zip › foods-904747-tex.zip!/Definitions/foods-904747-g007.pdf]

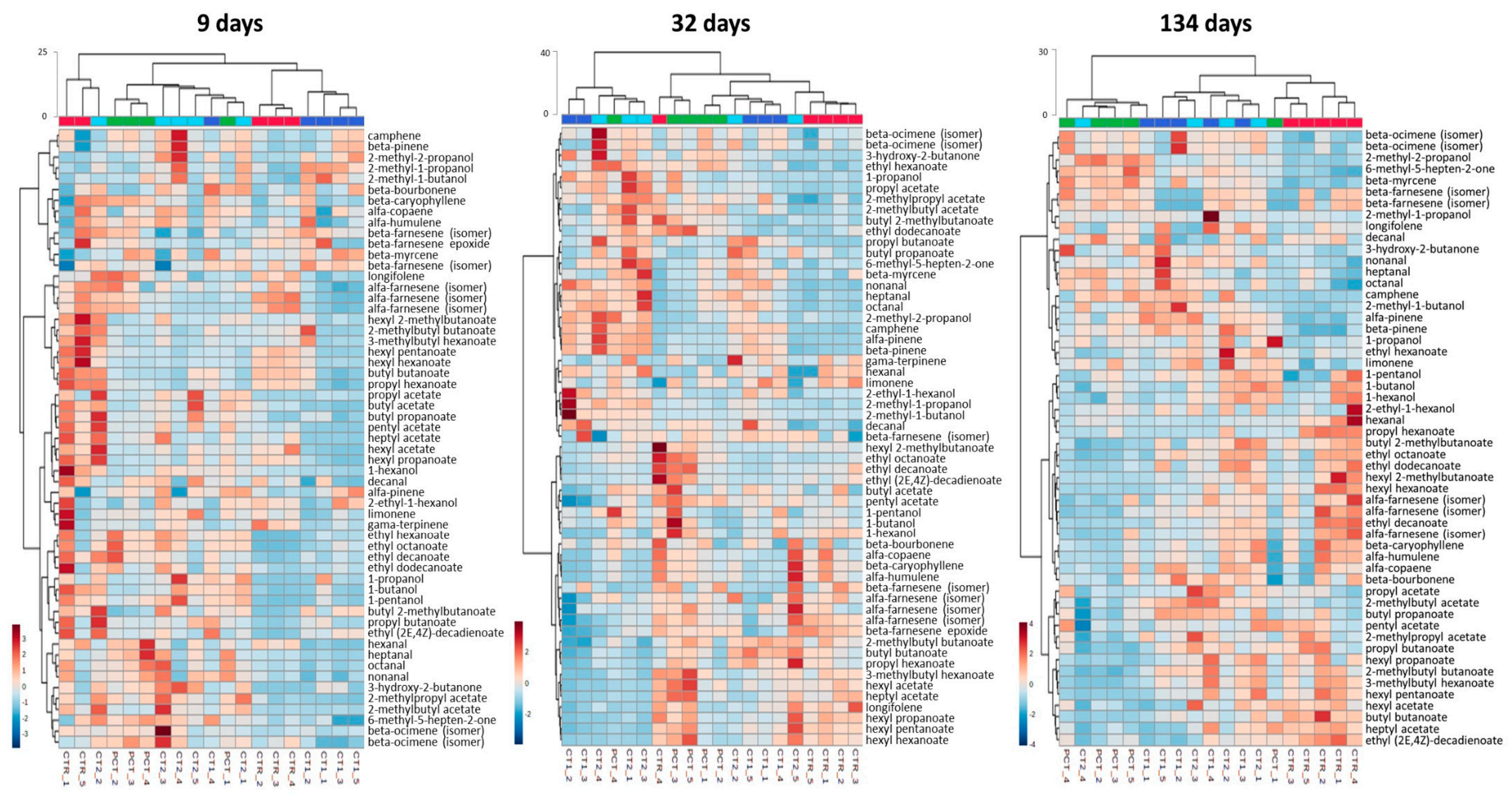
9 days
25
0
camphene
beta-pinene
2-methyl-2-propanol
2-methyl-1-propanol
2-methyl-1-butanol
beta-bourbonene
beta-caryophyllene
alfa-copaene
alfa-humulene
beta-farnesene (isomer)
beta-farnesene epoxide
beta-myrcene
beta-farnesene (isomer)
longifolene
alfa-farnesene (isomer)
alfa-farnesene (isomer)
alfa-farnesene (isomer)
hexyl 2-methylbutanoate
2-methylbutyl butanoate
3-methylbutyl hexanoate
hexyl pentanoate
hexyl hexanoate
butyl butanoate
propyl hexanoate
propyl acetate
butyl acetate
butyl propanoate
pentyl acetate
heptyl acetate
hexyl acetate
hexyl propanoate
1-hexanol
decanal
alfa-pinene
2-ethyl-1-hexanol
limonene
gama-terpinene
ethyl hexanoate
ethyl octanoate
ethyl decanoate
ethyl dodecanoate
1-propanol
1-butanol
1-pentanol
butyl 2-methylbutanoate
propyl butanoate
ethyl (2E,4Z)-decadienoate
hexanal
heptanal
octanal
nonanal
3-hydroxy-2-butanone
2-methylpropyl acetate
2-methylbutyl acetate
6-methyl-5-hepten-2-one
beta-ocimene (isomer)
beta-ocimene (isomer)
3
2
1
0
-1
-2
-3
CTR_1
CTR_5
CT2_2
PCT_2
PCT_3
PCT_4
CT2_3
CT2_4
CT2_5
CT1_4
PCT_1
CT2_1
CTR_2
CTR_3
CTR_4
CT1_2
CT1_1
CT1_3
CT1_5
32 days
40
0
beta-ocimene (isomer)
beta-ocimene (isomer)
3-hydroxy-2-butanone
ethyl hexanoate
1-propanol
propyl acetate
2-methylpropyl acetate
2-methylbutyl acetate
butyl 2-methylbutanoate
ethyl dodecanoate
propyl butanoate
butyl propanoate
6-methyl-5-hepten-2-one
beta-myrcene
nonanal
heptanal
octanal
2-methyl-2-propanol
camphene
alfa-pinene
beta-pinene
gama-terpinene
hexanal
limonene
2-ethyl-1-hexanol
2-methyl-1-propanol
2-methyl-1-butanol
decanal
beta-farnesene (isomer)
hexyl 2-methylbutanoate
ethyl octanoate
ethyl decanoate
ethyl (2E,4Z)-decadienoate
butyl acetate
pentyl acetate
1-pentanol
1-butanol
1-hexanol
beta-bourbonene
alfa-copaene
beta-caryophyllene
alfa-humulene
beta-farnesene (isomer)
alfa-farnesene (isomer)
alfa-farnesene (isomer)
alfa-farnesene (isomer)
beta-farnesene epoxide
2-methylbutyl butanoate
butyl butanoate
propyl hexanoate
3-methylbutyl hexanoate
hexyl acetate
heptyl acetate
longifolene
hexyl propanoate
hexyl pentanoate
hexyl hexanoate
2
0
-2
CT1_2
CT1_3
CT2_4
PCT_4
CT2_1
CT2_3
CTR_4
PCT_3
PCT_5
PCT_1
PCT_2
CT2_2
CT1_5
CT1_1
CT1_4
CT2_5
CTR_5
CTR_1
CTR_2
CTR_3
134 days
30
0
beta-ocimene (isomer)
beta-ocimene (isomer)
2-methyl-2-propanol
6-methyl-5-hepten-2-one
beta-myrcene
beta-farnesene (isomer)
beta-farnesene (isomer)
2-methyl-1-propanol
longifolene
decanal
3-hydroxy-2-butanone
nonanal
heptanal
octanal
camphene
2-methyl-1-butanol
alfa-pinene
beta-pinene
1-propanol
ethyl hexanoate
limonene
1-pentanol
1-butanol
1-hexanol
2-ethyl-1-hexanol
hexanal
propyl hexanoate
butyl 2-methylbutanoate
ethyl octanoate
ethyl dodecanoate
hexyl 2-methylbutanoate
hexyl hexanoate
alfa-farnesene (isomer)
alfa-farnesene (isomer)
ethyl decanoate
alfa-farnesene (isomer)
beta-caryophyllene
alfa-humulene
alfa-copaene
beta-bourbonene
propyl acetate
2-methylbutyl acetate
butyl propanoate
pentyl acetate
2-methylpropyl acetate
propyl butanoate
hexyl propanoate
2-methylbutyl butanoate
3-methylbutyl hexanoate
hexyl pentanoate
hexyl acetate
butyl butanoate
heptyl acetate
ethyl (2E,4Z)-decadienoate
4
2
0
-2
-4
PCT_4
CT2_4
PCT_2
PCT_3
PCT_5
CT1_1
CT1_5
CT1_2
CT2_3
CT1_4
CT2_2
CT1_3
CT2_1
PCT_1
CTR_3
CTR_5
CTR_2
CTR_1
CTR_4
