# Supplementary material for: The Impact of Plant-Based Coatings in “ROCHA” Pear Preservation during Cold Storage: A Metabolomic Approach
Source: Foods. 2020 Sep 15;9(9):1299. doi: 10.3390/foods9091299 (PMC7555455; doi:10.3390/foods9091299)
Supplement: Supplementary file 1 [file foods-09-01299-s001.zip › foods-904747-tex.zip!/Definitions/foods-904747-g008.pdf]

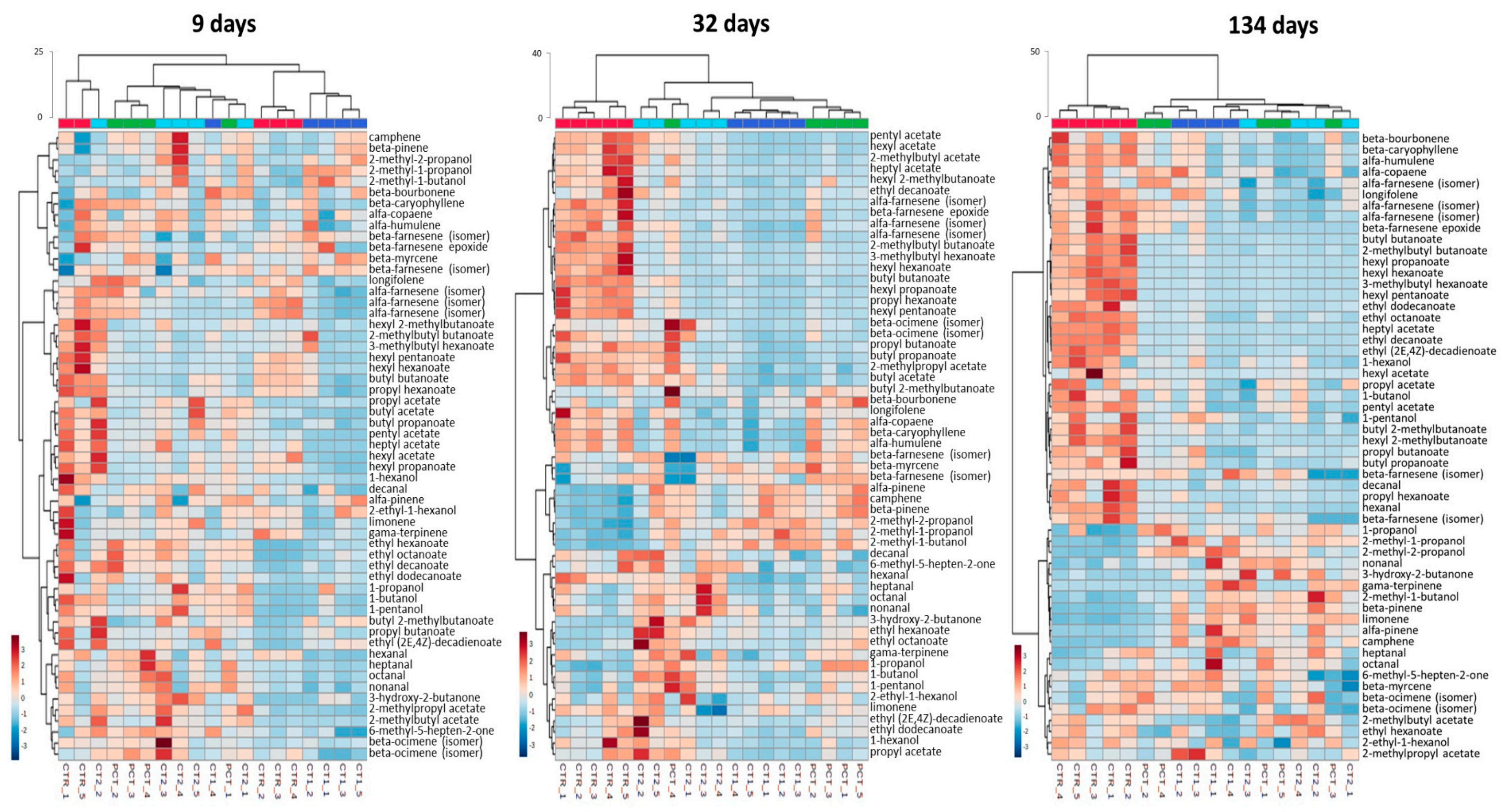

9 days
25
0
camphene
beta-pinene
2-methyl-2-propanol
2-methyl-1-propanol
2-methyl-1-butanol
beta-bourbonene
beta-caryophyllene
alfa-copaene
alfa-humulene
beta-farnesene (isomer)
beta-farnesene epoxide
beta-myrcene
beta-farnesene (isomer)
longifolene
alfa-farnesene (isomer)
alfa-farnesene (isomer)
alfa-farnesene (isomer)
hexyl 2-methylbutanoate
2-methylbutyl butanoate
3-methylbutyl hexanoate
hexyl pentanoate
hexyl hexanoate
butyl butanoate
propyl hexanoate
propyl acetate
butyl acetate
butyl propanoate
pentyl acetate
heptyl acetate
hexyl acetate
hexyl propanoate
1-hexanol
decanal
alfa-pinene
2-ethyl-1-hexanol
limonene
gama-terpinene
ethyl hexanoate
ethyl octanoate
ethyl decanoate
ethyl dodecanoate
1-propanol
1-butanol
1-pentanol
butyl 2-methylbutanoate
propyl butanoate
ethyl (2E,4Z)-decadienoate
hexanal
heptanal
octanal
nonanal
3-hydroxy-2-butanone
2-methylpropyl acetate
2-methylbutyl acetate
6-methyl-5-hepten-2-one
beta-ocimene (isomer)
beta-ocimene (isomer)
3
2
1
0
-1
-2
-3
CTR_1
CTR_5
CT2_2
PCT_2
PCT_3
PCT_4
CT2_3
CT2_4
CT2_5
CT1_4
PCT_1
CT2_1
CTR_2
CTR_3
CTR_4
CT1_2
CT1_1
CT1_3
CT1_5
32 days
40
0
pentyl acetate
hexyl acetate
2-methylbutyl acetate
heptyl acetate
hexyl 2-methylbutanoate
ethyl decanoate
alfa-farnesene (isomer)
beta-farnesene epoxide
alfa-farnesene (isomer)
alfa-farnesene (isomer)
2-methylbutyl butanoate
3-methylbutyl hexanoate
hexyl hexanoate
butyl butanoate
hexyl propanoate
propyl hexanoate
hexyl pentanoate
beta-ocimene (isomer)
beta-ocimene (isomer)
propyl butanoate
butyl propanoate
2-methylpropyl acetate
butyl acetate
butyl 2-methylbutanoate
beta-bourbonene
longifolene
alfa-copaene
beta-caryophyllene
alfa-humulene
beta-farnesene (isomer)
beta-myrcene
beta-farnesene (isomer)
alfa-pinene
camphene
beta-pinene
2-methyl-2-propanol
2-methyl-1-propanol
2-methyl-1-butanol
decanal
6-methyl-5-hepten-2-one
hexanal
heptanal
octanal
nonanal
3-hydroxy-2-butanone
ethyl hexanoate
ethyl octanoate
gama-terpinene
1-propanol
1-butanol
1-pentanol
2-ethyl-1-hexanol
limonene
ethyl (2E,4Z)-decadienoate
ethyl dodecanoate
1-hexanol
propyl acetate
3
2
1
0
-1
-2
-3
CTR_1
CTR_2
CTR_3
CTR_4
CTR_5
CT2_2
CT2_5
PCT_4
CT2_1
CT2_3
CT2_4
CT1_4
CT1_5
CT1_2
CT1_3
PCT_2
PCT_3
PCT_1
PCT_5
134 days
50
0
beta-bourbonene
beta-caryophyllene
alfa-humulene
alfa-copaene
alfa-farnesene (isomer)
longifolene
alfa-farnesene (isomer)
alfa-farnesene (isomer)
beta-farnesene epoxide
butyl butanoate
2-methylbutyl butanoate
hexyl propanoate
hexyl hexanoate
3-methylbutyl hexanoate
hexyl pentanoate
ethyl dodecanoate
ethyl octanoate
heptyl acetate
ethyl decanoate
ethyl (2E,4Z)-decadienoate
1-hexanol
hexyl acetate
propyl acetate
1-butanol
pentyl acetate
1-pentanol
butyl 2-methylbutanoate
hexyl 2-methylbutanoate
propyl butanoate
butyl propanoate
beta-farnesene (isomer)
decanal
propyl hexanoate
hexanal
beta-farnesene (isomer)
1-propanol
2-methyl-1-propanol
2-methyl-2-propanol
nonanal
3-hydroxy-2-butanone
gama-terpinene
2-methyl-1-butanol
beta-pinene
limonene
alfa-pinene
camphene
heptanal
octanal
6-methyl-5-hepten-2-one
beta-myrcene
beta-ocimene (isomer)
beta-ocimene (isomer)
2-methylbutyl acetate
ethyl hexanoate
2-ethyl-1-hexanol
2-methylpropyl acetate
3
2
1
0
-1
-2
-3
CTR_4
CTR_5
CTR_3
CTR_1
CTR_2
PCT_2
PCT_4
CT1_2
CT1_3
CT1_1
CT1_4
CT2_3
PCT_1
PCT_5
CT2_4
CT2_2
PCT_3
CT2_1
