# Supplementary material for: The Impact of Plant-Based Coatings in “ROCHA” Pear Preservation during Cold Storage: A Metabolomic Approach
Source: Foods. 2020 Sep 15;9(9):1299. doi: 10.3390/foods9091299 (PMC7555455; doi:10.3390/foods9091299)
Supplement: Supplementary file 1 [file foods-09-01299-s001.zip › foods-904747-tex.zip!/Definitions/foods-904747-g009.pdf]

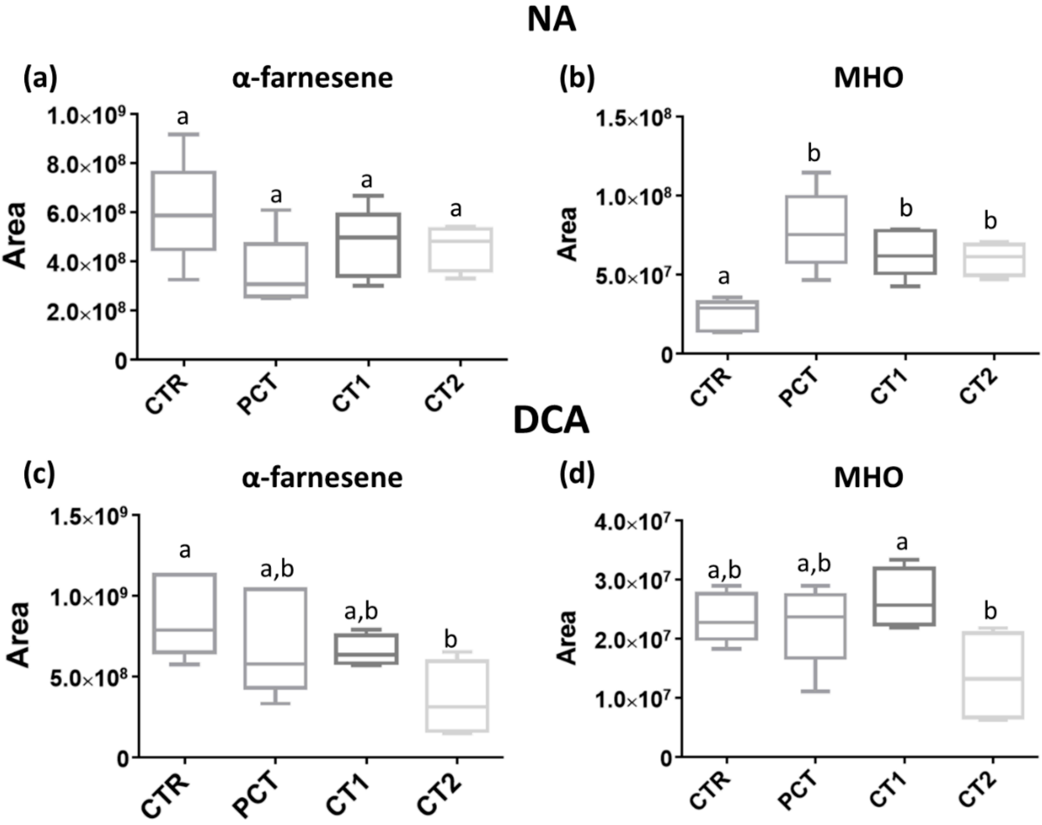
NA
(a)
α-farnesene
Area
1.0×10^9
8.0×10^8
6.0×10^8
4.0×10^8
2.0×10^8
0
a
a
a
a
CTR
PCT
CT1
CT2
(b)
MHO
Area
1.5×10^8
1.0×10^8
5.0×10^7
0
a
b
b
b
CTR
PCT
CT1
CT2
DCA
(c)
α-farnesene
Area
1.5×10^9
1.0×10^9
5.0×10^8
0
a
a,b
a,b
b
CTR
PCT
CT1
CT2
(d)
MHO
Area
4.0×10^7
3.0×10^7
2.0×10^7
1.0×10^7
0
a,b
a,b
a
b
CTR
PCT
CT1
CT2
