# Supplementary material for: The Impact of Plant-Based Coatings in “ROCHA” Pear Preservation during Cold Storage: A Metabolomic Approach
Source: Foods. 2020 Sep 15;9(9):1299. doi: 10.3390/foods9091299 (PMC7555455; doi:10.3390/foods9091299)
Supplement: Supplementary file 1 [file foods-09-01299-s001.zip › foods-904747-tex.zip!/Definitions/logo-ccby-eps-converted-to.pdf]

CC BY
